# Window[1]resorcin[3]arenes: A Novel Macrocycle Able to Self-Assemble to a Catalytically Active Hexameric Cage

**DOI:** 10.1021/jacsau.4c00097

**Published:** 2024-05-03

**Authors:** Tian-Ren Li, Chintu Das, Ivan Cornu, Alessandro Prescimone, GiovanniMaria Piccini, Konrad Tiefenbacher

**Affiliations:** † Department of Chemistry, University of Basel, Mattenstrasse 24a, 4058 Basel, Switzerland; ‡ Department of Biosystems Science and Engineering, ETH Zurich, Mattenstrasse 26, 4058 Basel, Switzerland; § Institute of Technical and Macromolecular Chemistry RWTH Aachen University, Worringerweg 2, 52074 Aachen, Germany

**Keywords:** host−guest systems, macrocycles, supramolecular chemistry, molecular cage, catalysis

## Abstract

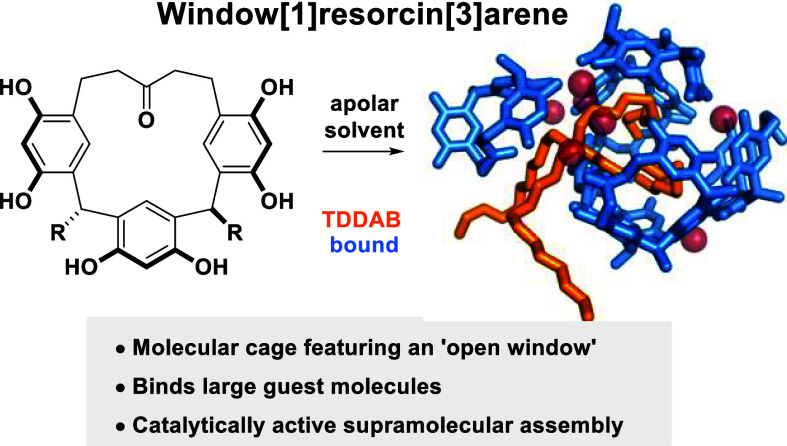

The hexameric resorcin[4]arene capsule has been utilized as one of the most versatile supramolecular capsule catalysts. Enlarging its size would enable expansion of the substrate size scope. However, no larger catalytically active versions have been reported. Herein, we introduce a novel class of macrocycles, named window[1]resorcin[3]arene (wRS), that assemble to a cage-like hexameric host. The new host was studied by NMR, encapsulation experiments, and molecular dynamics simulations. The cage is able to bind tetraalkylammonium ions that are too large for encapsulation inside the hexameric resorcin[4]arene capsule. Most importantly, it retained its catalytic activity, and the accelerated conversion of a large substrate that does not fit the closed hexameric resorcin[4]arene capsule was observed. Thus, it will help to expand the limited substrate size scope of the closed hexameric resorcin[4]arene capsule.

## Introduction

**Figure 1:**
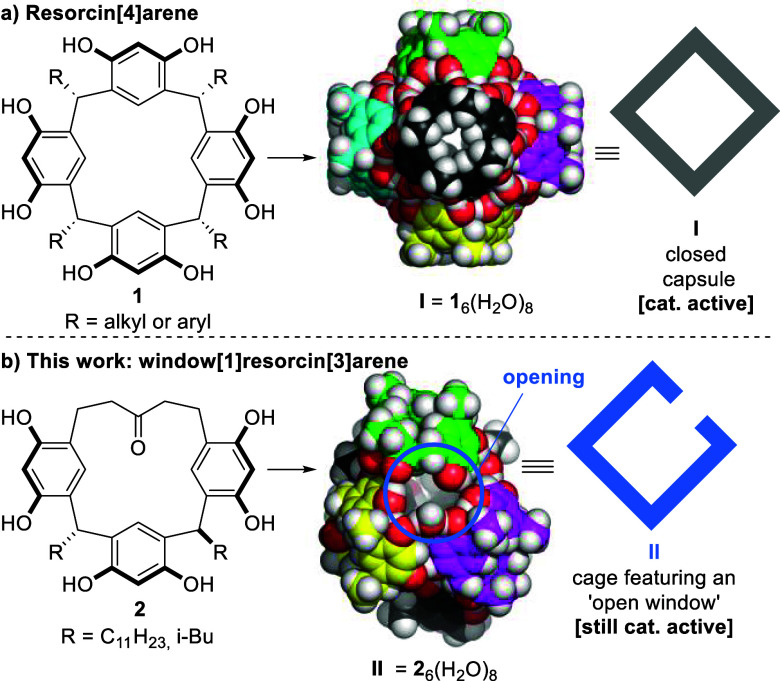
(a) Capsule **I**, possessing a closed structure, self-assembles from six resorcin[4]arene units **1** (displayed in different colors) and eight water molecules via 60 hydrogen bonds; (b) the new window[1]resorcin[3]arene macrocycle **2** is still able to self-assemble to an open cage-like assembly **II** that retains catalytic activity. On average eight water molecules are incorporated into the hydrogen bond network of **II**.

Resorcin[4]arene (**1**, Figure 1a) is a true workhorse in supramolecular chemistry.^1^ It is readily available on a large scale by a simple condensation of resorcinol with an aldehyde, typically under acid catalysis.^2−4^ Due to its bowl-shaped structure and the diverse modification options on its upper rim, it has been utilized as a versatile platform for the construction of supramolecular containers ranging from (hemi)carcerands^5−7^ to cavitands,^8−14^ molecular cages, and capsules.^15−21^ A true surprise was the finding that resorcin[4]arene (**1**) self-assembles to a large hexameric capsule **I** (Figure 1a), incorporating eight water molecules into the complex hydrogen bond network.^22−25^ With an inner volume of approximately 1400 Å^3^,^22^ it is one of the largest molecular capsules based on hydrogen bonds.^26−29^ Furthermore, it is, together with the Ga(III)–catecholate-based tetrahedral host^30^ investigated by the groups of Bergman, Raymond, and Toste,^31^ one of the most successful molecular capsule catalysts.^32−49^ However, due to the closed cavity of the molecular capsule, the reaction volume is finite. Although a slight size increase of the capsule with an excess of water was postulated,^50^ to our knowledge there are no reports about the catalyzed conversion of substrates that do not fit the cavity of capsule **I**. To expand the substrate size scope, three options seem in principle feasible: (1) the enlargement of the capsule. (2) The construction of a catalytically active cavitand that is open to one end. (3) The incorporation of openings in the closed capsule, to form a cage-like catalyst. Concerning (1), the enlargement of the capsule, attempts were made to form larger resorcin[4]arene-like building blocks based on naphthalene. However, no bowl-shaped macrocycles suitable for self-assembly were accessible.^51−54^ Expanded resorcin[n]arenes, containing five and even seven resorcin subunits have been described.^55^ However, to the best of our knowledge, there are no reports about their self-assembly to capsules related to **I**. Furthermore, there are reports about fascinating, very large anion-sealed capsules formed from resorcin[n]arenes.^56,57^ In these cases, however, reports about guest uptake and catalytic behavior are unknown to the best of our knowledge. While many new phenol-based macrocycles have been reported recently,^58−69^ only very few have structural similarity to resorcin[4]arene with a conformationally rigid bowl shape and unprotected phenol moieties.^29,70^ However, no enlarged catalytically active capsule has been reported from these efforts as of yet. (2) While cavitands have been successfully utilized as reaction containers,^9,13,46,71−73^ we considered it difficult to transfer the hydrogen bond network of capsule **I**, which seems essential for many reactions,^74−78^ to a covalent cavitand structure. Thus, (3), the opening of the closed capsule to a cage-like structure seemed to be the best option. This approach may facilitate the binding of guest molecules that are excluded from binding to the resorcin[4]arene capsule, as parts of the guest could protrude through an opening at the cage surface. While there have been many spectacular reports about the reaction acceleration inside molecular cages, for instance by the groups of Raymond/Bergman/Toste^31,42^ and Fujita,^32,49^ the expansion of capsule **I** into a cage-like catalyst would be of interest as capsule **I** displays a different catalytic behavior as the known cages. However, to preserve the catalytic activity of the original capsule, changes should be kept to a minimum. Water has been identified to play a crucial role in catalysis inside capsule **I**. It is responsible for the acidity of capsule **I**,^74,76^ it can function as a proton shuttle,^77^ or is even involved in a proton-wire mechanism,^78^ and thus would be highly likely required in catalytically active cage-like derivatives of capsule **I**. However, the question remains whether such closely related open structures are accessible and whether they would retain the unusual catalytic activity of the resorcin[4]arene hexamer. Here, we answer this question and present the synthesis of a new macrocyclic compound, which we propose to name window[1]resorcin[3]arene (wRS). Formally, one of the four resorcinol units of the parent resorcin[4]arene is replaced by an alkylidene chain (Figure 1b). The modification is tolerated in the self-assembly process, delivering a cage-like host **II**, featuring an “open window” (see the space-filling model in Figure 1b) in contrast to the closed capsule **I**. Interestingly, cage **II** is still catalytically active and is able to convert a substrate too large for conversion inside capsule **I**.

**Figure 2:**
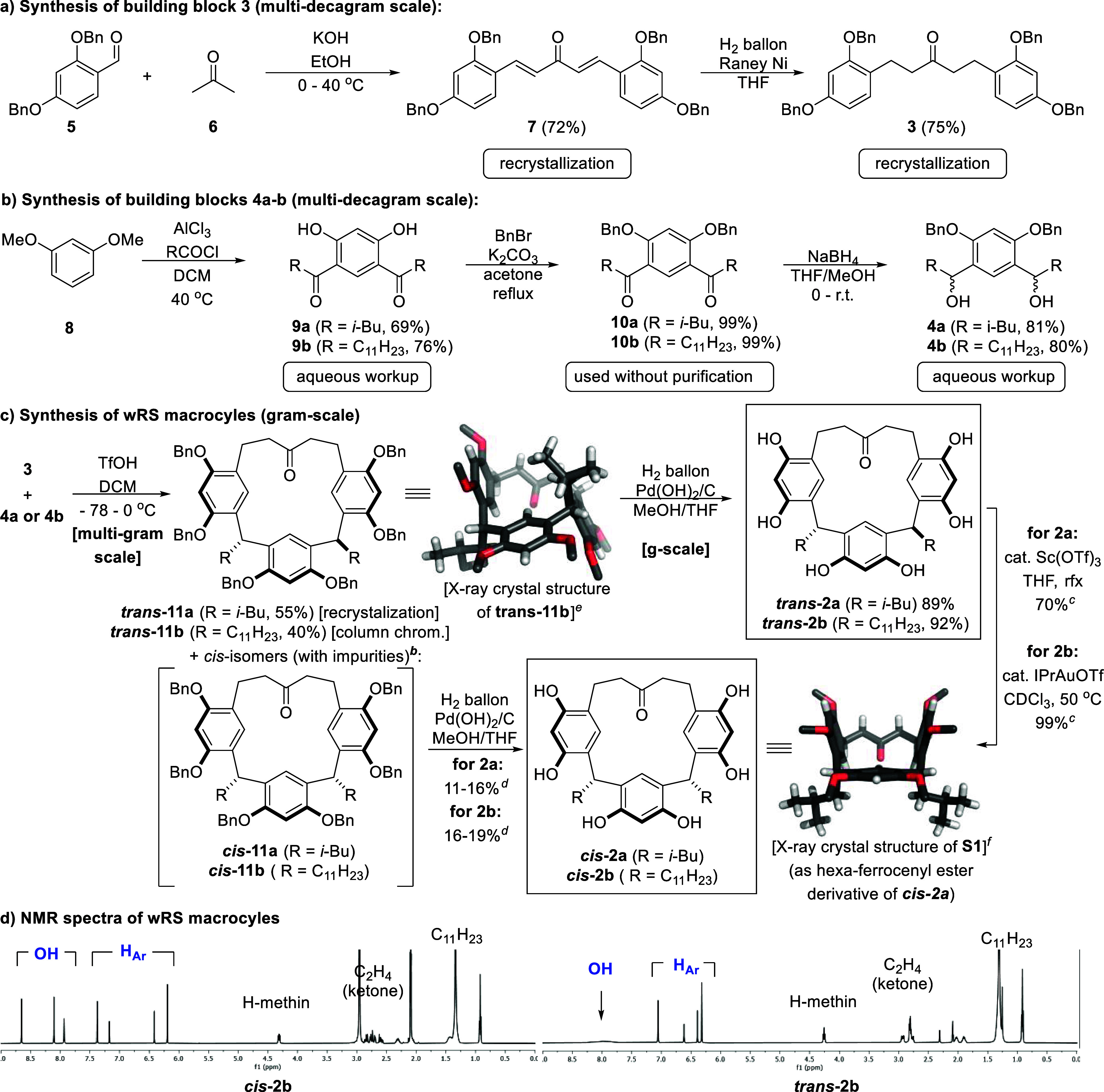
(a–c) Gram-scale synthesis of window[1]resorcin[3]arene **2**^*a*^ (d) ^1^H NMR spectra of *trans*- and *cis*-**2b** recorded in acetone-d_6_. ^*a*^See the Supporting Information section 2 for the detailed condition optimization. ^*b*^The purification of *cis***-11a** and *cis***-11b** was unsuccessful after cyclization; the crude *cis***-11a** and *cis***-11b** (mixed with some trans-isomers and unknown impurities) were used in the preparation of *cis***-2a** and *cis***-2b**. ^*c*^The yields described here represent the isolated yields of the epimerizations from *trans***-2a/2b** to *cis***-2a/2b**. ^*d*^The yields described here represent the overall yields in 2 steps, which were calculated based on compound **3**. The isolated yields vary slightly from batch to batch, presumably due to the inseparable impurities in *cis-***11a** and *cis-***11b**. *^e^*Bn-protecting groups are omitted for clarity. ^*f*^Ferrocenyl-esters are omitted for clarity; for the synthesis of S1, see SI, Chapter 3.2.

The synthesis of hetero-oligomeric macrocycles like **2** (Figure 1b) is a challenge as direct cyclization approaches that are successful for homo-oligomeric macrocycles like **1** are not feasible. At the outset of the project, we envisioned two synthetic strategies: (i) the cyclization of resorcinol-dimer **3** (Figure 2a) with resorcinol and an aldehyde in analogy to the synthesis of resorcin[4]arene. (ii) Alternatively, the merger of the key compounds **3** and **4** (Figure 2b) was envisioned. Resorcinol-dimer **3** functions as a nucleophilic reaction partner, while resorcinol derivative **4** functions exclusively as an electrophilic component, thus avoiding potential homo-oligomerization. After extensive optimization work, a reliable and scalable synthetic route to window[1]resorcin[3]arene **2** was established: Resorcinol-dimer **3** was synthesized from the known benzaldehyde derivative **5**,^79^ which was condensed with acetone to deliver dibenzylideneacetone **7**. The alkene hydrogenation turned out to be challenging, as undesired benzyl cleavage was observed under most reaction conditions. It was found that the use of THF as a solvent and the thorough washing of Raney-Ni with THF to completely remove residual water were essential for a clean conversion (see SI, Section 2.1 for more details). Under these optimized conditions compound **3** was obtained in a good overall yield of 54% over two steps. Importantly, no purification via column chromatography was required in the synthetic sequence leading to key compound **3**, enabling the facile synthesis of multidecagram quantities of material. With this material in hand, a direct three-component cyclization of **3**, resorcinol (unprotected or protected), and dodecanal was attempted. However, the formation of the homo-oligomeric resorcin[4]arene product **1** dominated, and no traces of the desired hetero-oligomeric product **2** were detected. To avoid homo-oligomerization, an alternative strategy was explored. The synthesis of key compounds **4a**–**b**, carrying different aliphatic R-residues, commenced with the double Friedel–Crafts acylation of resorcinol dimethyl ether (**8**) and the corresponding acyl chlorides. The methyl-protecting groups were concomitantly removed under the reaction conditions to deliver compounds **9a**–**b** in good yields. The crude material was directly benzyl-protected, followed by the reduction of the ketones, to deliver **4a**–**b** in high yields. Similarly to compound **3**, no purification via column chromatography was required during the whole sequence, enabling the facile synthesis of multidecagram quantities of material.

With the key building blocks **3** and **4a**–**b** in hand, acid-promoted macrocyclization was explored. After an extensive screening (see SI Chapter 2.2.1 for a summary), suitable conditions (1.0 equiv **3**, 1.2 equiv **4**, 0.1 equiv of triflic acid in dichloromethane) were identified that delivered the desired macrocycles in good yields. Interestingly two isomers were formed under all reaction conditions screened. They were identified via X-ray crystallography (Figure 2c, see SI Chapter 3.1 for larger depictions) as the *cis*- and *trans*-isomers regarding the two alkyl feet ‘R’. While the reaction temperature had some influence on the selectivity, the *trans*-isomer dominated under all of the conditions explored. Interestingly, the favored *trans*-isomer turned out to be the more useful isomer for the self-assembly process (see the discussion below). It was isolated in pure form, depending on the foot installed, via either recrystallization (***trans***-**11a**, 55%) or column chromatography (*trans***-11b**, 40%). After a final debenzylation, free-phenol *trans*-isomers **2a** and **2b** were obtained in high yields. The reactions were also scaled up to gram quantities without a reduction in isolated yields. ***Cis***-**2a**–**b** adopts a highly symmetric crown-like conformation in solution (see ^1^H NMR spectrum in Figure 2d). Although the benzyl-protected derivative *trans***-11a** crystallized in a chairlike conformation, the deprotected compounds ***trans***-**2a**–**b** display a very high symmetry in solution (Figure 2d). As only one methine signal is observed, this symmetry most likely stems from a rapid interconversion of the crown conformations. Metadynamics simulations also indicated that the crown conformation of ***trans***-**2** is preferred by ca. 30 kJ/mol over alternatives (see SI Chapter 5).

The respective *cis*-isomers ***cis***-**2a** and *cis***-2b** were obtained via deprotection of impure ***cis***-**11a** and *cis-***11b** in 11–16% and 16–19% yields, respectively, over two steps. Alternatively, the *cis*-isomers are also available via a Lewis-acid catalyzed isomerization of the respective *trans*-isomers, indicating that the *cis*-isomers are thermodynamic products (see SI Chapter 2.4 for details). The structure of the *cis*-isomers was confirmed by X-ray crystallography of the hexa-ferrocenyl ester of *cis***-2a** that displayed a crown conformation in the solid state (Figure 2c, see SI Chapter 5 for details). Also, the solution analysis via ^1^H NMR indicated a crown conformation (Figure 2d). The preference for the crown conformation for the *trans***-2b** isomer was further supported by classical metadynamics free energy simulations (see SI Chapter 5).

**Figure 3:**
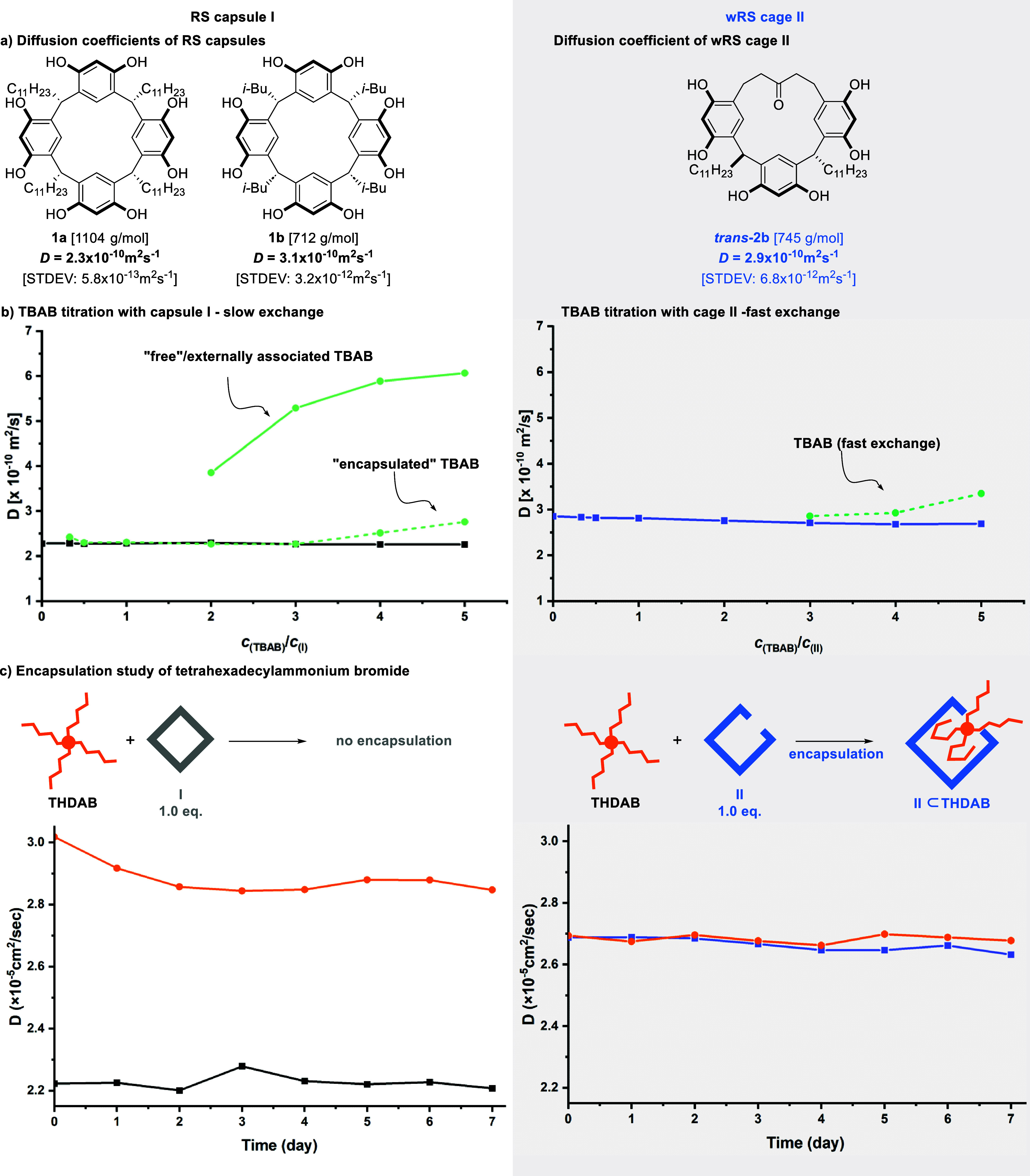
(a) *C11*-feet *trans*-**2b** forms a hexameric cage in CDCl_3_; its diffusion coefficient is in good agreement with the related resorcin[4]arene capsules; [wRS] = 3.33 mM. (b) Binding of tetrabutylammonium bromide (TBAB). Diffusion coefficients of capsule **I** (black square), cage **II** (blue square), and TBAB (green circle) are shown with increasing equiv of TBAB (0–5 equiv). Capsule **I** binds 1 equiv of TBAB in slow exchange. Cage **II** binds to TBAB in fast exchange. (c) Binding studies with 1 equiv of tetrahexadecylammonium bromide (THDAB). These experiments were performed in flame-sealed NMR tubes for 7 days; [wRS] = 3.33 mM. Diffusion coefficients of capsule **I** (black square), cage **II** (blue square), and THDAB (orange circle) are shown. No binding was observed for capsule **I**; Cage **II**, however, displayed binding, again in fast exchange. The experimental details are shown in SI.

After having identified the window[1]resorcin[3]arene macrocyclic products formed, self-assembly studies were performed with the C_11_-feet derivatives *cis/trans***-2b** due to their higher solubility in apolar solvents required for the self-assembly process. Surprisingly, the *cis*-isomer turned out to be barely soluble in CDCl_3_ (<0.1 mM in water-saturated chloroform), while *trans***-2b** displayed good solubility (>10 mM in water-saturated chloroform). Gratifyingly, *trans***-2b** is self-assembled in a chloroform solution. The assembly was studied via (1) diffusion-ordered spectroscopy (DOSY), and (2) guest encapsulation experiments. Additional characterization by mass spectrometry, reported for the resorcin[4]arene capsule **I**,^80^ was attempted but failed. The estimation of the assembly size via DOSY NMR indicated a hexameric assembly (*D* = 2.9 × 10^–10^ m^2^ s^–1^). The length and number of solubilizing feet have a significant influence on the overall size of the assembly and, thus, its diffusion coefficient. In contrast to the parent tetra-C_11_ resorcin[4]arene **1** (Figure 1a), macrocycle **2** only features two alkyl feet. Thus, as expected, the diffusion coefficient of the wRS assembly **II** lies between the values observed for the C_11_-feet hexamer **I** (*D* = 2.3 × 10^–10^ m^2^ s^–1^),^81^ and its *i*Bu-feet derivative (*D* = 3.4 × 10^–10^ m^2^ s^–1^, Figure 3a). Furthermore, the experimentally determined hydrodynamic radius is in very good agreement with the size of a hexameric molecular model (see SI Chapter 4.2). It is to be expected that the reduced number of solubilizing alkyl feet on *cis/trans***-2b** leads to a generally reduced solubility in apolar solvents compared to **1**. However, the large solubility difference between the two isomers (*cis/trans***-2b**) was initially puzzling. Due to the fewer solubilizing feet, large portions of the less soluble core part of the assembly are exposed to the solvent. We assume that the *trans*-orientation of the feet enables better coverage of these exposed surface areas, enabling a much higher solubility of the *trans*-isomer as compared to the *cis*-isomer (see also the model in the SI Figure S21).

After the size of the assembly was established, its properties for guest uptake were investigated. First, the uptake of tetrabutylammonium bromide (TBAB) was studied via ^1^H and DOSY NMR titrations, and compared to the binding properties inside capsule **I**. Capsule **I** binds 1 equiv of TBAB in slow exchange at the ^1^H NMR chemical shift time scale. The addition of a second equivalent leads to the appearance of “free”/externally associated^82^ TBAB signals that diffuse significantly faster than the encapsulated one (Figure 3b). In contrast to capsule **I**, TBAB was encapsulated inside cage **II** in fast exchange on the ^1^H NMR chemical shift time scale (see SI Chapter 4.3 for details). Due to signal overlap, the diffusion coefficient for TBAB was only determined for ≥3 equiv (Figure 3b).

Even more obvious differences between cage **II** and capsule **I** were observed with very large ammonium salts. The addition of 1 equiv of tetrahexadecylammoinum bromide (THDAB) to capsule **I** did not lead to binding, even after 7 days (Figure 3c). This is not surprising as it is known that **I** encapsulates ammonium salts up to tetraoctylammonium bromide (TOAB) but fails to bind larger ones due to the size limitations of the closed cavity.^82^ However, as also observed by Cohen and co-workers, large ammonium salts interact with the capsule from the outside, resulting in slightly reduced diffusion coefficients for the ammonium molecules due to this fast exchange,^82^ similar to what is observed with THDAB (Figure 3c). In contrast, cage **II**, can bind the large THDAB guest, as indicated by the similar diffusion values (Figure 3c, right side). This value (*D* approximately 2.7 × 10^–10^ m^2^ s^–1^), is slightly lower than the one for cage **II** (*D* = 2.9 × 10^–10^ m^2^ s^–1^), and substantially lower than for free THDAB (*D* approximately 4.5 × 10^–10^ m^2^ s^–1^). This may indicate the formation of a slightly larger assembly in fast exchange on the NMR time scale, in agreement with the postulated penetration of some alkyl chains through the window of cage **II**. Furthermore, tetradodecylammonium bromide (TDDAB) and tetraoctylammonium bromide (TOAB) were investigated under similar conditions. This was of interest as they display much higher diffusion coefficients (5.4 and 6.5 × 10^–10^ m^2^ s^–1^, respectively) than the larger THDAB. Thus, their interactions with capsule **I** and cage **II** should lead to even more significant differences in the diffusion coefficients observed. Indeed, in both cases, the complex diffused at similar low values (approximately 2.8–2.9 × 10^–10^ m^2^ s^–1^, see Table 1), supporting the conclusion that these large ammonium guests bind inside cage **II**, while they do not fit the closed cavity of the capsule **I**. As mentioned above, the reduced diffusion coefficients of the salts in the presence of capsule **I** are likely caused by interactions with the capsule from the outside.^82^ However, only if guest uptake is observed are the diffusion values of the ammonium salts and capsule identical, clearly differentiating the behavior of capsule **I** and cage **II** (Table 1).

**Table 1:** Binding Studies of Ammonium Salts with Capsule **I** and Cage **II**^a^

*D* (×10^–10^ m^2^ s^–1^)	TBAB (C4)	TOAB (C8)	TDDAB (C12)	THDAB (C16)
free guest	7.7	6.5	5.4	4.5
**I**/guest (difference)	2.3/2.3^b^(0)	2.3/3.4^c^(1.1)	2.3/3.2^c^(0.9)	2.2/2.9^c^(0.7)
**II**/guest (difference)	2.9/2.9^c^^,^^d^(0)	2.9/2.9^c^(0)	2.8/2.8^c^(0)	2.7/2.7^c^(0)

a *c*(host) = *c*(guest) = 3.33 mM in CDCl_3_; all samples were incubated at 50 °C for 24 h before measurement.

b Slow exchange on the ^1^H NMR time scale.

c Fast exchange on the ^1^H NMR time scale.

d Due to the signals overlapping between wRS and TBAB, TBABF_4_ was used as the guest molecule in this case.

**Figure 4:**
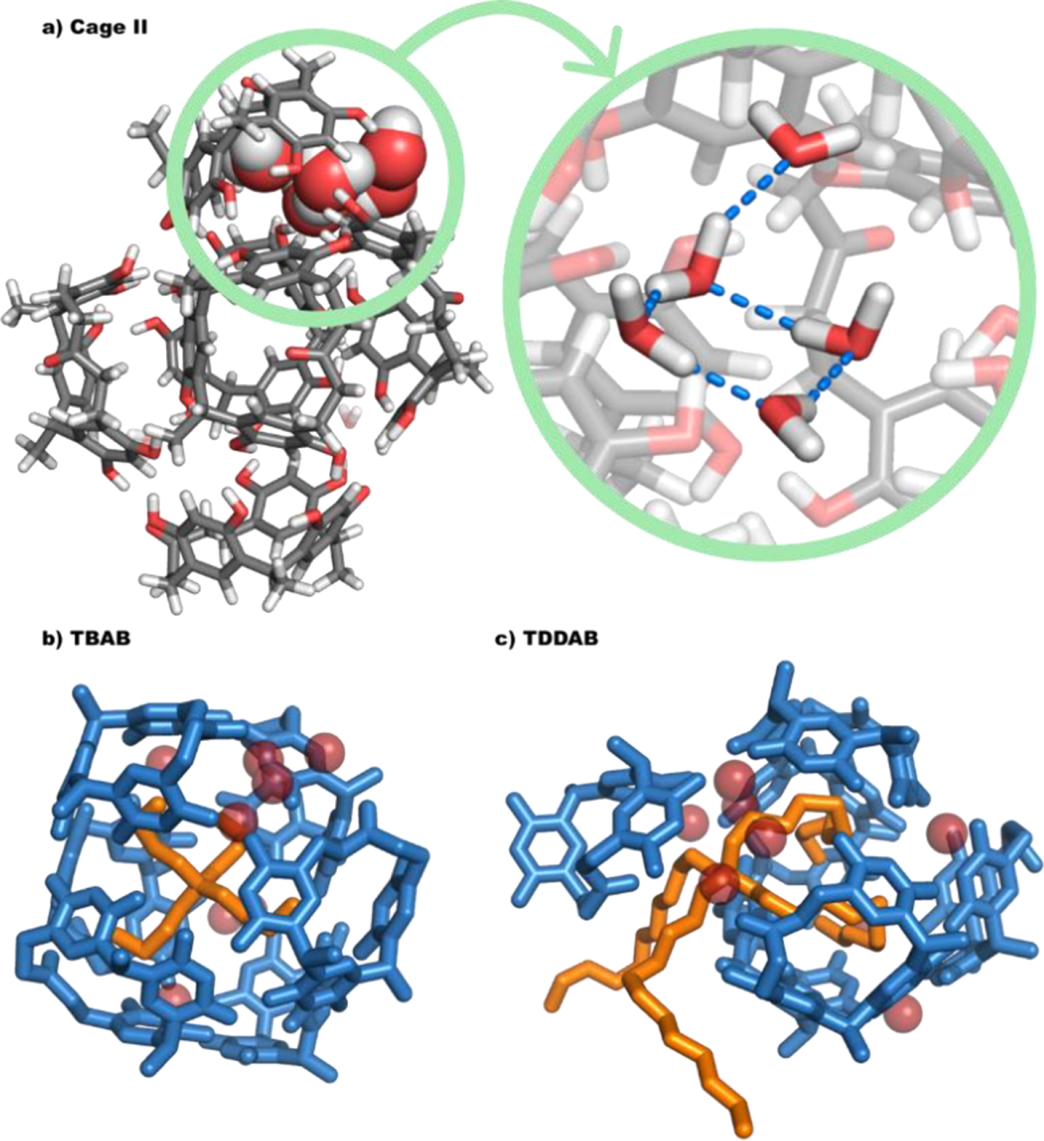
(a) Snapshot extracted from the equilibrium simulation of cage **II**. The system is a highly dynamical, distorted hexamer in which the water molecules are less regularly organized than in resorcin[4]arene capsule **I**, and tend to form resilient and organized cluster structures at corner sites like the one highlighted in the lime-green circle. (b, c) Host–guest structures for TBAB and TDDAB and cage **II**. For simplicity, the ammonium guests are depicted in orange, the window[1]resorcin[3]arenes in blue, and the water molecules as red semitransparent spheres, whereas hydrogen atoms have been removed for clarity.

To unravel the assembly dynamics and encapsulation properties of cage **II** and learn about its structural features, we utilized classical molecular dynamics simulations. These simulations reveal that the structural stability of solvated cage **II** without any additional guest molecule is largely maintained by an average of 20 intermolecular hydrogen bonds (see SI Figure S19 for hydrogen bond analysis plot). A significant structural attribute is a dynamic opening that contains on average a cluster of water molecules (see Figure 4a), with other additional water molecules located at separate corners within cage **II**. This opening is critical, as it serves as the structural defect allowing for the encapsulation of larger guest molecules, such as TDDAB.

Subsequently, we analyzed the dynamics of cage **II** with the encapsulation of TBAB. As expected, cage **II** successfully encloses TBAB, primarily due to the ample internal cavity that can accommodate the fully relaxed TBAB molecule, as previously observed in the closed capsule **I**.^23^ Concurrently, we observed a reduction in the size of the cage’s opening, with water molecules rearranging to effectively encapsulate the TBAB molecule. Remarkably, this configuration also maintained, on average, around 15 intermolecular hydrogen bonds, indicative of the structural resilience of cage **II** (see also SI Figure S19).

We also examined the assembly’s mean squared displacement (MSD) which is proportional to the diffusion coefficient (*D*). It is found that the diffusion coefficient of the free TBAB molecule (, see Table 2) is higher than that of the encapsulated one (, see Table 2). Figure S18b illustrates how the MSD values of cage **II** and encapsulated TBAB are similar to one another, demonstrating the stability of encapsulation. A further indication of a successful encapsulation is shown by the alignment of the MSD of cage **II** and TBAB as shown in Figure S18b resulting in a comparable diffusion coefficient of cage **II** (, see Table 2) to that of TBAB. Subsequently, we focused on the encapsulation of the larger TDDAB substrate. As TDDAB is much bulkier than that of TBAB, two of the hydrocarbon chains in this instance are seen to have protruded from the opening to make room for the large TDDAB inside cage **II**. This accommodation implies a distortion of the hexameric structure with a large displacement of one calixarene unit. Similar to the previous case, the water cluster again reconstructs on the surface of the host–guest complex, participating in intermolecular H-bonding that keeps cage **II** dynamically assembled. Although the assembly structure fluctuates more than in the case of TBAB because the two arms outside cage **II** are exposed to the solvent and, thus, more flexible than those inside, cage **II** is stable enough to keep the self-assembled structure intact throughout the simulation. The MSD and corresponding diffusion coefficient calculation shows that the encapsulated TDDAB molecule shows a lower diffusion coefficient (, Table 2) than that of free TDDAB (, Table 2). Further evidence that cage **II** can hold large guest molecules comes from Figure S18b, which shows that the MSD of encapsulated TDDAB and that of cage **II** are comparable; which yields a similar diffusion coefficient of cage **II** (, Table 2) and TDDAB. Structures extracted from the simulation for the host–guest complexes with the two substrates under investigation are reported in Figure 4b,c, respectively.

**Table 2:** Diffusion Coefficients of Cage **II**, TBAB, TDDAB, and Their Corresponding Host–Guest Structures

	*D*
Cage **II**	∼2.8
TBAB	∼7.9
TBAB @ cage **II**	∼3.0
TDDAB	∼4.7
TDDAB @ cage **II**	∼ 2.6

To further validate the dynamic stability of all the assembled structures, the root-mean-square deviation (RMSD) with respect to a highly ordered hexameric reference structure optimized at 0 K was measured throughout the whole simulations. This measurement, therefore, detects any dynamic deviation from the ideal assembled structure (see Figure S18). It can be observed that for both cases, despite a general deviation from the ideal structure that is expected due to finite temperature effects and/or volume changes upon encapsulation, the value of RMSD remains constant apart from thermal fluctuations. Furthermore, the simulations indicated that on average eight water molecules are incorporated into the hydrogen bond network of cage **II** (see SI Figure 20).

**Figure 5:**
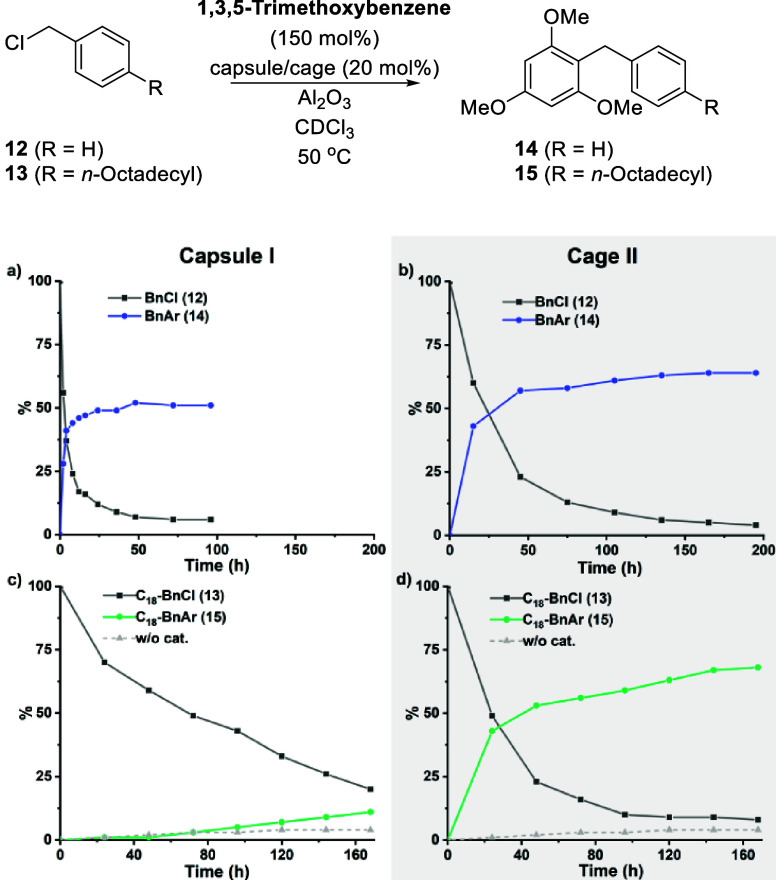
Reaction kinetics for the conversion of benzyl chloride with (a) capsule **I** and (b) cage **II** as catalysts, respectively; and the conversion of **13** with (c) capsule **I** and (d) cage **II** as catalysts, respectively. See the Supporting Information Section 7 for details.

After having demonstrated the ability of cage **II** to bind larger guests than the closed capsule **I**, we aimed to answer the final question: Is the cage still catalytically active? As mentioned before, this would be highly desirable, as previous attempts to obtain larger hydrogen-bond-based catalytically active capsules have not been successful. The Friedel–Crafts reaction, recently reported by the Gaeta and Neri group was chosen as it enables the facile modification of substrate size via the attachment of alkyl chains.^76^ Indeed, cage **II** turned out to be catalytically active in the reaction of benzyl chloride (**12**) and 1,3,5-trimethoxybenzene (Figure 5a). The direct comparison to the results of the resorcin[4]arene capsule (Figure 5b) indicated a rather similar catalytic activity. While the conversion rate is slightly reduced in the case of cage **II**, the overall yield is comparable to or even slightly improved. The substrate conversion was high (>90%), while the yield plateaued at approximately 60%, likely due to capsule/cage alkylation as also observed in previous studies.^83−85^ Importantly, no background reaction was observed in the absence of the capsule or cage (see SI Section 7.2). As indicated by the encapsulation studies discussed before, the “open window” of cage **II** should enable the binding of large substrates excluded in capsule **I**. Therefore, the conversion of 4-octadecyl benzyl chloride (**13**) was studied. As expected, capsule **I** did not accelerate the reaction to a significant extent (Figure 5c). The formation of the product (green line) was only slightly faster than the background reaction in the absence of the capsule (dotted gray line). However, cage **II** indeed catalyzed the reaction, nearly as efficiently as for the smaller substrate (Figure 5d), indicating that indeed the opening of cage **II** enables the conversion of substrates that do not fit the closed capsule **I**. This observation is in good agreement with the open-window model of cage **II** and the guest uptake experiments with the large ammonium salts (Table 1), as well as with the molecular dynamics simulations. To rule out the possibility of the Friedel–Crafts reaction taking place on the outer surface of cage **II**, two additional control experiments were carried out (see SI Chapter 7 for details). Blocking cage **II** with the high-affinity guest tetraethylammonium tetrafluoroborate (TEABF_4_, 100 mol %) suppressed its catalytic activity, and no product **15** was detected. Second, replacing the macrocycle with its subunit (4-hexylresorcinol) also failed to deliver Friedel–Crafts product **15**.

## Conclusions

In summary, we presented (1) the design and gram-scale synthesis of a structurally novel macrocycle, termed window[1]resorcin[3]arene (wRS), that is formally formed by the replacement of one resorcinol unit in resorcin[4]arene with an alkylidene chain. Interestingly, the *trans* isomer turned out to be much more soluble than the thermodynamically more stable *cis* product. (2) The *trans*-macrocycle is able to self-assemble to a hexameric cage of similar size to the hexameric resorcin[4]arene capsule. However, due to an opening, it can bind guests much larger than the closed capsule counterpart. Tetraalkyl ammonium guests were bound in fast exchange on the ^1^H NMR chemical shift time scale. (3) Most importantly, it was demonstrated that the cage retains the catalytic activity, and is able to catalyze the Friedel–Crafts reaction of substrates too large to be converted inside the resorcin[4]arene capsule. Thus, this cage represents the first example of a supramolecular assembly that can expand the substrate size scope of the closed resorcin[4]arene capsule. As this capsule is one of the most successfully applied molecular containers for catalysis, we believe that this finding is of high significance, as it enables the exploration of reactivity space not accessible via the closed hexameric resorcin[4]arene capsule.

## Methods

Representative procedure for the Friedel–Crafts reactions catalyzed by capsule/cage.

*Trans*-**2b** (30.0 μmol, 22.4 mg, equal to 5.00 μmol of cage **II**) and Al_2_O_3_ basic (50.0 mg) were weighed into a 2.5 mL screw-cap glass vial, and 500 μL of CDCl_3_ was added. The mixture was homogenized by careful sonicating and shaking. 1,3,5-Trimethoxybenzene (37.5 μmol, 6.31 mg) was added followed by benzyl chloride (25.0 μmol, 2.99 μL). After adding tetraethylsilane (10.0 μmol as the internal standard), the reaction mixture was placed in a 50 °C heating block. The material conversion and product formation were monitored by ^1^H NMR.

## Data Availability

The source data for Figures 1–5 and Tables 1 and 2 were deposited on Zenodo (zenodo.org/records/10222323)

## Supporting Information Available

Experimental details and NMR spectra of new compounds (PDF)
X-ray crystallographic data for *trans-*11a (CIF)
X-ray crystallographic data (CIF)

## Supplementary Material

au4c00097_si_001.pdf

au4c00097_si_002.cif

au4c00097_si_003.cif
