# Supplementary material for: Window[1]resorcin[3]arenes: A Novel Macrocycle Able to Self-Assemble to a Catalytically Active Hexameric Cage
Source: JACS Au. 2024 May 3;4(5):1901–10. doi: 10.1021/jacsau.4c00097 (PMC11134363; doi:10.1021/jacsau.4c00097)

Window[1]resorcin[3]arenes: A novel macrocycle able to self-assemble to a catalytically active hexameric cage

Tian-Ren Li,[a] Chintu Das,[c] Ivan Cornu,[a] Alessandro Prescimone,[a] GiovanniMaria Piccini,*[c] and Konrad Tiefenbacher* [a,b]

[a]Department of Chemistry, University of Basel, Mattenstrasse 24a, 4058 Basel, Switzerland

[b]Department of Biosystems Science and Engineering, ETH Zurich, Mattenstrasse 26, 4058 Basel, Switzerland.

[c]Institute of Technical and Macromolecular Chemistry, RWTH Aachen University, Worringerweg 2, 52074 Aachen Germany

E-mail: piccini@itmc.rwth-aachen.de

E-mail: konrad.tiefenbacher@unibas.ch; tkonrad@ethz.ch

Table of Contents

1. General information **2**

2. Synthetic Procedures and Characterization Data **4**

2.1 Synthesis of the key building blocks *4*
2.2 Acid-promoted macrocycle formation *10*
2.3 Synthesis of trans-window[1]resorcin[3]arene *14*
2.4 Synthesis of cis-window[1]resorcin[3]arene *17*
2.5 Comparison of NOESY NMR spectra *22*

3. Crystallographic Structures and X-Ray Data **24**

3.1 Crystallographic structures *24*
3.2 Preparation of the global ferrocenyl-protected derivative *26*
3.3 X-ray data *28*

4. Assembly Investigation **33**

4.1 The comparison of ^{1}H NMR spectra of trans-2b in difference solvents *33*
4.2 Estimation of the hydrodynamic radius of cage II in $CDCl_3$ *34*
4.3 Dissociation study of cage II with MeOH *36*
4.4 Binding studies of the window[1]resorcin[3]arenes cage II with tetrabutylammonium salts *37*
4.5 Binding studies of resorcin[4]arene capsule I with tetrabutylammonium bromide (TBAB) *41*
4.6 The comparison of capsule I & cage II with tetrabutylammonium salts as the guest molecules *43*
4.7 Binding studies of the window[1]resorcin[3]arene cage II with tetrahexadecylammonium bromide (THDAB) *45*
4.8 Guest encapsulation study of tetrahexadecylammonium bromide (THDAB) with both window[1]resorcin[3]arene cage II and resorcin[4]arene capsule I *47*
4.9 Assembly study of the mixture of capsule I and cage II *49*

5. Computational Study **50**

6. Model of Cage II **60**

7. Application of Window[1]resorcin[3]arene Cage II in Friedel–Crafts Reaction **61**

7.1 Synthesis of 4-octadecyl benzyl chloride *61*
7.2 Friedel–Crafts reactions catalyzed by capsule/cage *63*
7.3 Control experiments *70*

8. References **72**

9. NMR-Spectra of New Compounds **74**

1. General information

Experimental. Reactions were carried out under an atmosphere of argon unless otherwise indicated. For the synthetic applications of the window[1]resorcin[3]arenes (wRS), no precaution against air and moisture was taken. Analytical thin-layer chromatography (TLC) was performed on Merck silica gel 60 F_{254} glass-baked plates, which were analyzed after exposure to standard staining solutions (CAM: cerium ammonium molybdate or basic $KMnO_4$).

All NMR experiments were performed on a Bruker Avance III NMR spectrometer operating at 400 MHz and a Bruker Avance Neo NMR spectrometer operating at 500 MHz proton frequency. The instruments were equipped with a direct observe 5-mm BBFO smart probe or a direct observe 5-mm BBFO FB probe with a self-shielded z-gradient (500 MHz). The experiments were performed at 295 K and 298 K (500 MHz) and the temperature was calibrated using a methanol standard showing accuracy within +/-0.2 K. Chemical shifts of ^1H NMR and ^{13}C NMR are given in ppm by using $CHCl_3$ and $CDCl_3$ (7.26 ppm and 77.00 ppm, respectively). Coupling constants (*J*) are reported in Hertz (Hz). Standard abbreviations indicating multiplicity were used as follows: s (singlet), d (doublet), t (triplet), dd (doublet of doublets), ddd (doublet of doublet of doublets), dt (doublet of triplets), m (multiplet), q (quartet), hept (heptet), sept (septet) and br (broad signal).

All DOSY-NMR experiments were performed on a Bruker Avance III HD four-channel NMR spectrometer operating at 600.13 MHz proton frequency. The instrument was equipped with a cryogenic 5mm four-channel QCI probe (H/C/N/F) with a self-shielded z-gradient. The experiments were performed at 298 K and the temperature was calibrated using a methanol standard showing accuracy within +/- 0.2 K. For the PFGSE (pulsed-field gradient spin echo) diffusion experiment, the sample was placed in a 3 mm outer diameter tube, and the 3 mm tube was then inserted in a standard 5 mm round-bottom tube. This setup ensured a negligible temperature gradient on the sample even inside a cryogenic probe. The PFGSE experiments were performed using a bipolar gradient pulse sequence.[1] The sigmoidal intensity decrease was fitted with a two-parameter fit (I0 and diffusion coefficient D) with the DOSY routine implemented in topspin 3.6.1 [Bruker Biospin GmbH]. A typically observed intensity decrease is depicted below (Figure 1).

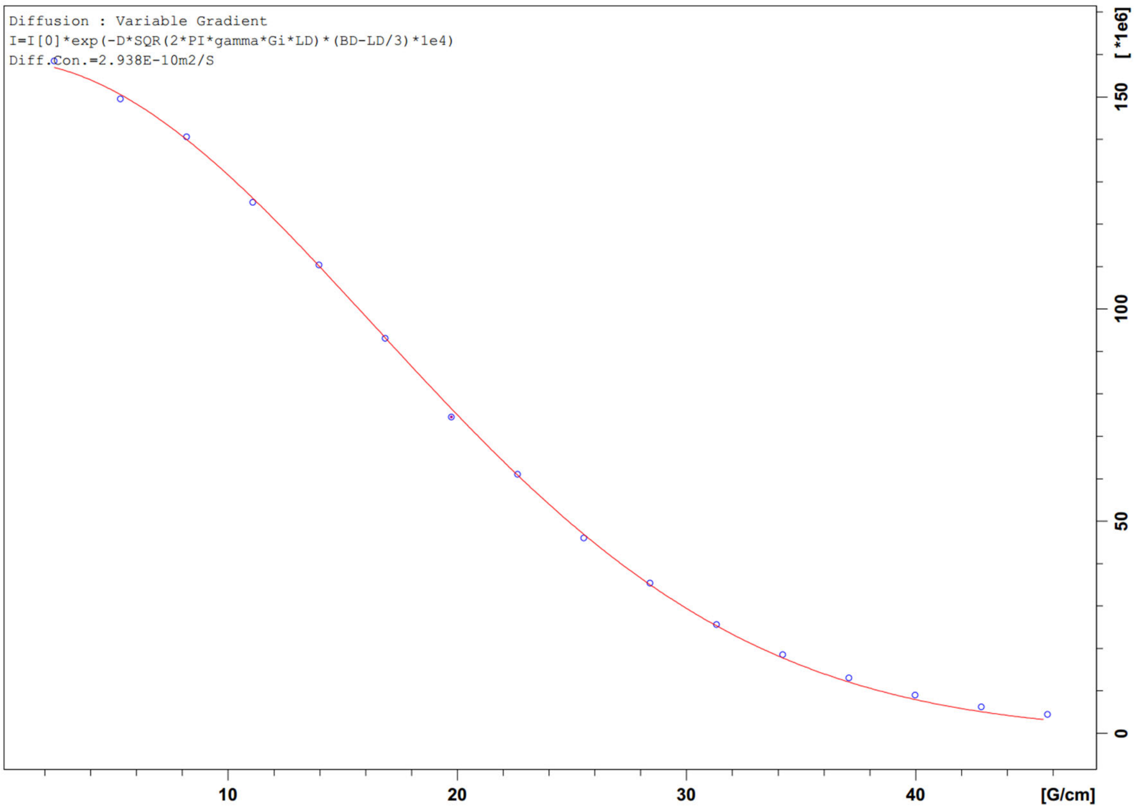

Figure S1: A typically observed intensity decrease in $CDCl_3$.

Mass spectroscopy using electron-ionization (ESI) was performed on an LCMS-2020 mass spectrometer instrument by SHIMADZU via direct injection. High-resolution mass spectra were obtained on a Thermo Scientific LTQ-FT Ultra via electrospray ionization (ESI) or a Finnigan MAT 8200 (EI) (ESI source parameters for positive polarity mode were: spray voltage, 4.0 kV; capillary temperature, 275 °C; capillary voltage, 48 V; and tube lens, –120 V).

Sources of chemicals. Commercial reagents were purchased from Sigma Aldrich, Alfa Aesar, Acros, Fluorochem, and Combi-block and were used as received with the following exceptions:

Solvents used in column chromatography separation were distilled at atmospheric pressure before use; 4 Å molecular sieves, Al_2O_3 neutral, and Al_2O_3 basic were purchased from Sigma Aldrich and activated by heating to 150 ºC overnight under vacuum; Deuterated chloroform ($CDCl_3$, 99.8%, stabilized over silver foil) was purchased from Cambridge Isotope Laboratories, and was passed through a short pad of Al_2O_3 basic before usage.

2. Synthetic Procedures and Characterization Data

2.1 Synthesis of the key building blocks

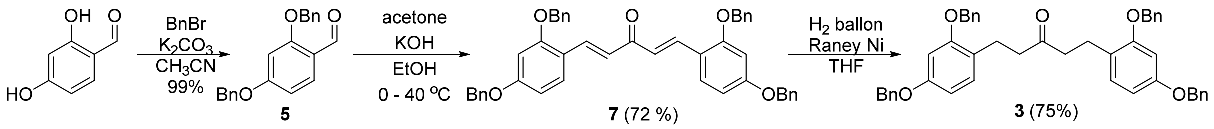

Scheme S1. The synthetic route toward building block **3**

Compound 4 *was synthesized following the literature-reported procedure*[2] *with slight modifications.* Benzyl bromide (24.5 mL, 205 mmol) was added into a mixture of 2,4-dihydroxybenzaldehyde (13.8 g, 100 mmol) and potassium carbonate (34.6 g, 250 mmol) in acetone (200 mL). The mixture was refluxed overnight. After the full consumption of the starting material, the reaction was allowed to cool to room temperature. The mixture was filtered through a short silica gel pad and the filter cake was washed well with acetone. The organic phase was concentrated on a rotavap. The crude product was redissolved into ethyl acetate, washed with sodium carbonate (2.0 M aq.) and brine, and then dried over anhydrous sodium sulfate. The solution was concentrated to give the title compound **5** as a yellowish solid (31.8 g, 99% yield), which is pure enough to be used directly in the next step.

The ^{1}H and ^{13}C NMR spectra of ***5*** *were in good agreement with the reported one.*[2]

1,5-bis(2,4-bis(benzyloxy)phenyl)penta-1,4-dien-3-one (7)

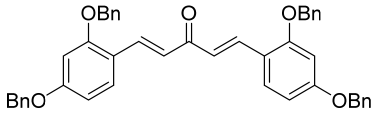

Chemical Formula: $C_{45}H_{38}O_5$
Molecular Weight: 658.79

Potassium hydroxide (pellets, 2.92 g, 52.0 mmol) was mixed with ethanol (16 mL) in a 50 mL flask. The KOH pellets were dissolved after vigorously stirring at room temperature for 30 min. 2,4-bis(benzyloxy)benzaldehyde **5** (12.7 g, 40.0 mmol) was added to the mixture to form a yellowish sludge. After cooling the reaction mixture to 0 °C with an ice-water bath, acetone (1.48 mL, 20.0 mmol) was added slowly via a syringe. The reaction was stirred for 2 hours at room temperature, then another 4 hours at 40 °C. The yellow suspension was then slowly poured into 100 mL of ice water. After neutralization with 3.20 mL AcOH and vigorously stirring for 1 hour, the mixture was allowed to stand in a 4 °C fridge for 2 hours. The yellow precipitate was filtrated and washed with cold water and cold EtOH/water (1:2 v/v) solution. The product was dried in the filtration funnel overnight by sucking air through the pad. The product **7** obtained (9.47 g,

72% yield) was pure enough for the next transformation. For the NMR sample, a small amount of the titled compound **7** was purified by column chromatography (EtOAc/Pentane = 1:4).

Yellow solid, 72% yield;

^{1}H NMR (600 MHz, $CDCl_3$) δ (ppm) ^{1}H NMR (600 MHz, $CDCl_3$) δ 8.06 (d, J = 16.0 Hz, 2H), 7.53 (d, J = 8.5 Hz, 2H), 7.46 – 7.40 (m, 12H), 7.40 – 7.34 (m, 6H), 7.34 – 7.30 (m, 2H), 7.09 (d, J = 16.0 Hz, 2H), 6.64 – 6.60 (m, 4H), 5.10 (s, 4H), 5.08 (s,4H).

^{13}C NMR (150 MHz, $CDCl_3$) δ (ppm) 189.9, 161.7, 159.0, 137.7, 136.3, 136.3, 130.2, 128.6, 128.6, 128.1, 127.9, 127.5, 127.1, 124.5, 117.6, 106.6, 100.6, 70.3, 70.1.

ESI-MS for: $C_{45}H_{39}O_5$ $[M+H]^+$: calcd 659.28, found 659.55;

HRMS (ESI) for: $C_{45}H_{38}O_5Na$ $[M+Na]^+$: calcd 681.2611, found 681.2600.

1,5-Bis(2,4-bis(benzyloxy)phenyl)pentan-3-one (3)

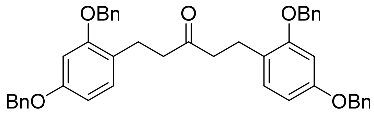

Chemical Formula: $C_{45}H_{42}O_5$
Molecular Weight: 662.83

Raney Ni (200 mg, water-coated) was weighed into a three-neck flask and washed 6 times with THF. The flask was then connected to a high vacuum pump for 3 hours to thoroughly remove the remaining H_2O. (Note: we found that thoroughly removing the residual water is crucial, as otherwise, the yield of the desired product decreases due to the debenzylation). Afterward, a solution of compound **7** (6.59 g, 10.0 mmol) in 50 mL THF was added. The reaction was stirred at room temperature under a hydrogen atmosphere (triple balloon). After the reaction completion (monitored by TLC), H_2 was released and the solution was carefully poured into another container so that the nickel remained at the magnetic stirring bar in the original flask. The solvent was evaporated on a rotavap. The crude product was purified by recrystallization from acetonitrile to obtain the titled compound **3** in 75% yield (4.95 g).

White solid, 75% yield;

^{1}H NMR (500 MHz, $CDCl_3$) δ (ppm) 7.46 – 7.27 (m, 20H), 7.00 (d, J = 8.2 Hz, 2H), 6.57 (d, J = 2.4 Hz, 2H), 6.48 (dd, J = 8.2, 2.4 Hz, 2H), 5.01 (s, 8H), 2.84 (dd, J = 8.4, 6.8 Hz, 4H), 2.64 (dd, J = 8.3, 6.9 Hz, 4H).

^{13}C NMR (125 MHz, $CDCl_3$) δ (ppm) 210.7, 158.4, 157.3, 137.0, 130.3, 128.6, 128.5, 128.0, 127.8, 127.5, 127.1, 122.4, 105.3, 100.6, 70.2, 69.8, 43.0, 24.5.

ESI-MS for: $C_{45}H_{42}O_5Na$ $[M+Na]^+$: calcd 685.29, found 685.50;

HRMS (ESI) for: $C_{12}H_{22}O_5Na$ $[M+Na]^+$: calcd 685.2924, found 685.2911.

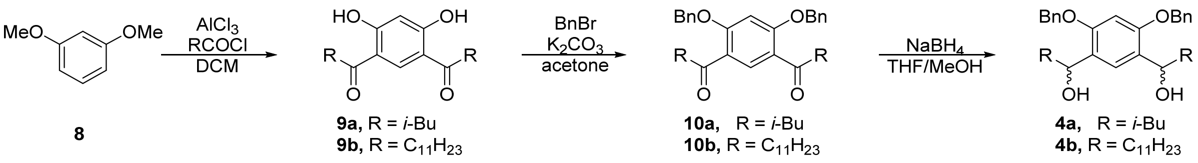

Scheme S2. The synthetic route towards building blocks **4a** and **4b.**

1,1'-(4,6-dihydroxy-1,3-phenylene)bis(3-methylbutan-1-one) (9a)

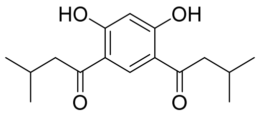

Chemical Formula: $C_{16}H_{22}O_4$
Molecular Weight: 278.3

For compound **8**, a solution of 1,3-dimethoxybenzene (2.76 g, 20.0 mmol) and isovaleryl chloride (5.06 g, 42.0 mmol) in DCM (20 mL) was added dropwise into a stirring mixture of anhydrous aluminum (III) chloride (5.60 g, 42.0 mmol) and DCM (20 mL) at 0 °C. Once the addition was complete, the reaction mixture was slowly warmed to room temperature and stirred for 48 hours and 8 hours more at 40 °C. After the full conversion of the starting materials (monitored by TLC), the reaction mixture was poured into ice water/conc. HCl (400 mL, 3:1 v/v). The organic layer was separated and washed with water and brine. After being concentrated on a rotavap, the crude product was triturated and sonicated in MeOH to afford compound **8** in 69 % yield (3.84 g). The product obtained was pure enough for the next transformation. For the NMR sample, crude product **8** was refined by recrystallization in MeOH.

White solid, 69% yield;

^{1}H NMR (500 MHz, $CDCl_3$) δ (ppm) 13.11 (s, 2H), 8.22 (s, 1H), 6.39 (s, 1H), 2.79 (d, J = 6.9 Hz, 4H), 2.28 (dq, J = 13.4, 6.7 Hz, 2H), 1.03 (d, J = 6.8 Hz, 12H).

^{13}C NMR (125 MHz, $CDCl_3$) δ (ppm) 204.6, 168.9, 135.1, 113.3, 105.0, 46.5, 25.8, 22.7.

ESI-MS for: $C_{16}H_{21}O_4$ [M-H]$^-$: calcd 277.14, found 277.10;

HRMS (ESI) for: $C_{16}H_{22}O_4Na$ [M+Na]$^+$: calcd 301.1410, found 301.1407.

1,1'-(4,6-dihydroxy-1,3-phenylene)bis(dodecan-1-one) (9b)

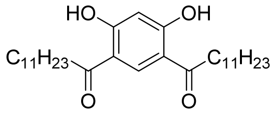

Chemical Formula: $C_{30}H_{50}O_4$
Molecular Weight: 474.73

Compound 9b was prepared following the same procedure as compound **9a** by using lauroyl chloride instead of isovaleryl chloride. The title compound was obtained in 76% yield.

White solid, 76% yield;

^{1}H NMR (600 MHz, $CDCl_3$) δ (ppm) 13.04 (s, 2H), 8.24 (s, 1H), 6.38 (d, J = 2.0 Hz, 1H), 2.92 (t, J = 7.5 Hz, 4H), 1.74 (q, J = 7.4 Hz, 4H), 1.39 – 1.24 (m, 32H), 0.87 (t, J = 7.0 Hz, 6H).
^{13}C NMR (150 MHz, $CDCl_3$) δ (ppm) 204.9, 168.8, 134.7, 113.0, 105.0, 37.7, 31.9, 29.6, 29.5, 29.4, 29.3, 29.3, 29.3, 24.7, 22.7, 14.1.
ESI-MS for: $C_{30}H_{49}O_4$ [M-H]$^-$: calcd 473.36, found 473.45;
HRMS (ESI) for: $C_{30}H_{49}O_4$ [M-H]$^-$: calcd 473.3636, found 473.3658.

1,1'-(4,6-bis(benzyloxy)-1,3-phenylene)bis(3-methylbutan-1-one) (10a)

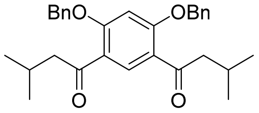

Chemical Formula: $C_{30}H_{34}O_4$
Molecular Weight: 458.6

For compound **10a**, benzyl bromide (4.87 mL, 41.0 mmol) was added into a mixture of compound **9a** (5.57 g, 20.0 mmol) and potassium carbonate (6.91 g, 50.0 mmol) in acetone (50 mL). The mixture was refluxed overnight. After the full consumption of the starting material, the reaction was allowed to cool to room temperature. The mixture was filtered through a short silica pad and the filter cake was washed well with acetone. The organic phase was concentrated on a rotavap. The crude product was redissolved in ethyl acetate, washed with sodium carbonate (2M, aq.) and brine, then dried over anhydrous sodium sulfate. The organic solution was concentrated to give compound **10a** as a white solid (9.17 g, 99% yield), which is pure enough to be used directly in the next step. For the NMR sample, the product was further purified by recrystallization from EtOAc/pentane (1:3 v/v).

White solid, 99% yield;

^{1}H NMR (600 MHz, $CDCl_3$) δ (ppm) 8.23 (s, 1H), 7.41 (dd, J = 21.3, 4.6 Hz, 10H), 6.57 (s, 1H), 5.16 (s, 4H), 2.80 (d, J = 7.0 Hz, 4H), 2.19 (dt, J = 13.5, 6.7 Hz, 2H), 0.86 (d, J = 6.9 Hz, 12H).
^{13}C NMR (150 MHz, $CDCl_3$) δ (ppm) 200.0, 161.5, 135.3, 134.1, 128.7, 128.4, 127.5, 121.9, 97.5, 71.0, 52.5, 24.8, 22.5.
ESI-MS for: $C_{30}H_{35}O_4$ [M+H]$^+$: calcd 459.25, found 459.15;
HRMS (ESI) for: $C_{30}H_{34}O_4Na$ [M+Na]$^+$: calcd 481.2349, found 481.2348.

1,1'-(4,6-bis(benzyloxy)-1,3-phenylene)bis(dodecan-1-one) (10b)

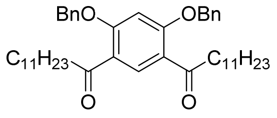

Chemical Formula: $C_{44}H_{62}O_4$
Molecular Weight: 654.98

Compound **10b** was prepared following the same procedure as compound **10a**. The title compound was obtained in 99% yield.

White solid, 99% yield;

^{1}H NMR (600 MHz, $CDCl_3$) δ (ppm) 8.21 (s, 1H), 7.41 – 7.36 (m, 10H), 6.53 (s, 1H), 5.13 (s, 4H), 2.87 (t, J = 7.6 Hz, 4H), 1.60 (d, J = 7.2 Hz, 4H), 1.29 – 1.18 (m, 32H), 0.88 (t, J = 7.0 Hz, 6H).

^{13}C NMR (150 MHz, $CDCl_3$) δ (ppm) 200.5, 161.6, 135.4, 134.3, 128.8, 128.5, 127.5, 121.8, 97.5, 71.0, 43.7, 31.9, 29.6, 29.5, 29.4, 29.3, 29.3, 24.5, 22.7, 14.1.

ESI-MS for: $C_{44}H_{63}O_4$ [M+H]$^+$: calcd 655.47, found 655.45;

HRMS (ESI) for: $C_{44}H_{62}O_4Na$ [M+Na]$^+$: calcd 677.4540, found 677.4527.

1,1'-(4,6-bis(benzyloxy)-1,3-phenylene)bis(3-methylbutan-1-ol) (4a)

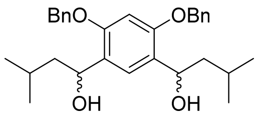

Chemical Formula: $C_{30}H_{38}O_4$
Molecular Weight: 462.6300

Compound **10a** (4.58 g, 10.0 mmol) was dissolved into a solvent mixture of EtOH/THF (40 mL, 1:1). After cooling to 0 ºC, $NaBH_4$ (1.51 g, 40.0 mmol) was added slowly into the mixture. The reaction was stirred at room temperature for 4 hours (monitored by TLC). After the total consumption of **10a**, the reaction was carefully quenched with NH_4Cl (saturated aq.). The biphasic mixture was extracted three times with DCM. The combined organic layer was washed with brine and dried over anhydrous sodium sulfate. After filtration, the solvent was removed on a rotavap (temperature below 35 ºC) to afford compound **4a** as a white solid (3.66 g, 81% yield), which is pure enough for the next transformation.

Compound **4a** is unstable on silica-gel columns. Therefore, no further purification was conducted.

White solid, 81% yield;

^{1}**H NMR** (500 MHz, $CDCl_3$) δ (ppm) 7.42 – 7.32 (m, 11H), 6.55 (s, 1H), 5.05 (s, 4H), 5.01 – 4.96 (m, 2H), 2.28 (dd, J = 14.7, 5.6 Hz, 2H), 1.78 – 1.70 (m, 4H), 1.60 – 1.53 (m, 2H), 0.95 – 0.90 (m, 12H).

^{13}**C NMR** (125 MHz, $CDCl_3$) δ (ppm) 155.4, 155.4, 136.6, 136.6, 128.7, 128.7, 128.3, 128.1, 128.1, 127.3, 127.0, 125.9, 125.8, 125.4, 125.3, 98.1, 98.1, 70.5, 68.5, 68.3, 46.8, 46.7, 24.9, 23.3, 23.3, 22.1, 22.1.

ESI-MS for: $C_{30}H_{38}O_4Na$ $[M+Na]^+$: calcd 485.27, found 485.30;

HRMS (ESI) for: $C_{30}H_{38}O_4Na$ $[M+Na]^+$: calcd 485.2662, found 485.2655.

1,1'-(4,6-bis(benzyloxy)-1,3-phenylene)bis(dodecan-1-ol) (4b)

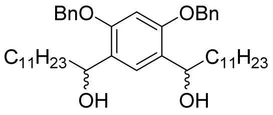

Chemical Formula: $C_{44}H_{66}O_4$
Molecular Weight: 659.01

Compound **4b** was synthesized following the same procedure. The titled compound was obtained in 80% yield.

Compound **4b** is unstable on silica-gel columns. Therefore, no further purification was conducted.

White solid, 80% yield;

^{1}**H NMR** (600 MHz, $CDCl_3$) δ (ppm) 7.39 – 7.31 (m, 10H), 7.25 (s, 1H), 6.53 (s, 1H), 5.03 (s, 4H), 4.86 (ddd, J = 13.2, 10.4, 5.8 Hz, 2H), 2.31 (dd, J = 19.8, 5.7 Hz, 2H), 1.76 (dddd, J = 23.7, 15.2, 8.6, 4.0 Hz, 4H), 1.45 – 1.38 (m, 2H), 1.29 – 1.21 (m, 34H), 0.86 (t, J = 7.0 Hz, 6H).

^{13}**C NMR** (150 MHz, $CDCl_3$) δ (ppm) 155.5, 136.6, 136.6, 128.7, 128.1, 127.3, 125.7, 125.6, 125.4, 125.4, 98.0, 70.5, 70.5, 70.3, 37.5, 37.5, 31.9, 29.7, 29.6, 29.6, 29.6, 29.6, 29.4, 26.1, 26.1, 22.7, 14.1.

ESI-MS for: $C_{44}H_{66}O_4Na$ $[M+Na]^+$: calcd 681.49, found 681.45;

HRMS (ESI) for: $C_{44}H_{66}O_4Na$ $[M+Na]^+$: calcd 681.4853, found 681.4844.

2.2 Acid-promoted macrocycle formation

2.2.1 Optimization of the reaction conditions

The optimization of the acid-promoted macrocyclic formation was performed with ketone **3** and di-benzylic alcohol **4b**. The results are summarized in the following table.

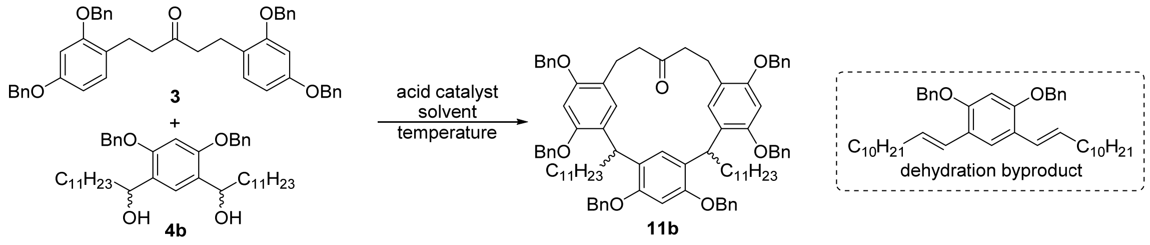

Entry	Solvent	Acid	Loading	Temp. (°C)	Yield (%)[b]	*cis:trans*[c]
1	EtOH	Conc. HCl	0.1 mL	reflux	-[d]	-
2	EtOH	Conc. HCl	0.1 mL	25	-[d]	-
3	EtOH	HCl (1N)	0.1 mL	25	-[d]	-
4	EtOH	HCl (0.1 N)	0.1 mL	25	-[e]	-
5	CH_3CN	MsOH	0.1 eq.	25	37	-
6	CH_3CN	TsOH	0.1 eq.	25	-[e]	-
7	CH_3CN	TfOH	0.1 eq.	25	50	45:55
8	CH_3CN	Amberlyst 15 H^+	100 mg	25→75	-[e]	-
9	CH_3CN	$Sc(OTf)_3$	0.1 eq.	25	51	49:51
10	CH_3CN	TfOH	0.1 eq.	0	57	44:56
11	DCM	TfOH	0.1 eq.	25	48	49:51
12	DCM	BF_3Et_2O	0.1 eq.	0	complex[h]	-
13	DCM	TfOH	0.1 eq.	0	55	47:53
14	DCM	TfOH	0.1 eq.	-20	64	41:59
15	DCM	TfOH	0.1 eq.	-78→0	70	37:63
16[f]	DCM	TfOH	0.1 eq.	-78→0	72	38:62
17[g]	DCM	TfOH	0.1 eq.	-78→0	78	37:63

[a]Unless noted otherwise, reactions were performed with **3** (20.0 μmol, 1.0 equiv.), **4b** (24.0 μmol, 1.2 equiv.), acid promoter (10 mol%), in solvents (500 μL) for 2 hours. [b]Isolated yield of both isomers combined. [c]Determined by ^{1}H NMR analysis. [d]Substrates remained. [e]Dehydration byproduct was detected as the main product. [f]1.00 mL DCM was used. [g]2.00 mL DCM was used. [h]Trace amount of 11b was detected by NMR, however it was inseparable from unknown impurities.

For the large-scale preparation, see section 2.2.2.

2.2.2 Synthetic procedures and characterizations

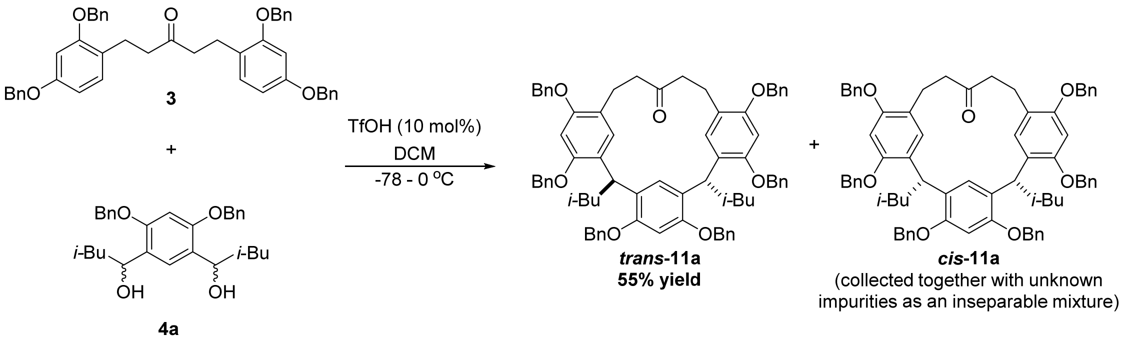

Scheme S3. Synthesis of benzyl-protected macrocycles ***trans*- & *cis*-11a**

In a 500 mL round-bottom flask, intermediate **3** (1.99 g, 3.00 mmol) and **4a** (1.67 g, 3.60 mmol) were dissolved into 300 mL DCM. After cooling to -78 °C with a dry-ice cooling bath, trifluoromethanesulfonic acid (26.5 μL, 300 μmol) was added dropwise with 2.00 mL DCM. Then the reaction was gradually warmed to 0 °C in 3 hours. After the total consumption of material **3** (monitored by TLC), Et_3N (100 μL) was added to quench the reaction. DCM was evaporated on a rotavap to afford the crude product as a yellow oil. The precipitation of *trans*-product was induced by adding a solvent mixture of MeOH/EtOAc/pentane (1:1:20). After triturated and sonicated, a white powder was collected by filtration, which was then washed with the above-mentioned solvent mixture, dried under a high vacuum to afford the ***trans*-11a** in 55% yield.

The filtrate was combined and concentrated on a rotavap. The majority of impurities were removed by column chromatography, and the remaining mixture contains mainly ***cis*-11a** along with a trace amount of inseparable *trans*-isomer and other unknown impurities. This mixture was collected and used in the synthesis of ***cis*-2a** without further purification (see section 2.4).

***trans*-1^4,1^6,3^4,3^6,5^4,5^6-hexakis(benzyloxy)-2,4-diisobutyl-1,3,5(1,3)-tribenzenacyclodecaphan-8-one (*trans*-11a)**

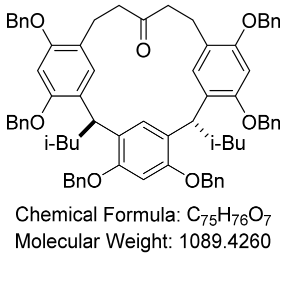

Chemical Formula: $C_{75}H_{76}O_7$
Molecular Weight: 1089.4260

White solid, 55% yield (*trans*-isomer);

^{1}H NMR (600 MHz, $CDCl_3$) δ (ppm) 7.44 – 7.27 (m, 20H), 7.19 – 7.13 (m, 6H), 7.07 – 7.04 (m, 4H), 6.94 (s, 2H), 6.48 (s, 2H), 6.41 (s, 1H), 6.27 (s, 1H), 5.05 (d, J = 11.8 Hz, 2H), 4.98

(d, J = 11.8 Hz, 2H), 4.88 – 4.83 (m, 6H), 4.79 (d, J = 11.8 Hz, 2H), 4.68 (d, J = 11.9 Hz, 2H), 3.16 (ddd, J = 14.5, 8.0, 4.3 Hz, 2H), 2.86 – 2.72 (m, 4H), 2.67 (ddd, J = 14.6, 8.4, 4.3 Hz, 2H), 1.61 (ddd, J = 13.0, 8.7, 5.8 Hz, 2H), 1.40 (ddt, J = 35.4, 13.3, 7.0 Hz, 4H), 0.80 (d, J = 6.5 Hz, 6H), 0.68 (d, J = 6.4 Hz, 6H).

^{13}C NMR (150 MHz, $CDCl_3$) δ (ppm) 210.2, 155.5, 155.0, 154.4, 137.9, 137.6, 137.5, 128.6, 128.5, 128.3, 128.2, 127.8, 127.6, 127.4, 127.4, 127.3, 127.2, 127.2, 127.2, 125.6, 121.3, 99.2, 97.8, 70.6, 70.4, 70.4, 44.3, 42.4, 32.6, 25.7, 23.3, 23.3, 22.5.

ESI-MS for: $C_{75}H_{76}O_7Na$ [M+Na]$^+$: calcd 1111.55, found 1112.25;

HRMS (ESI) for: $C_{75}H_{76}O_7Na$ [M+Na]$^+$: calcd 1111.5483, found 111.5484.

The single-crystal sample of ***trans*-11a** that is suitable for structure resolution was obtained by slow evaporation of a solution of ***trans*-11a** (4.00 mg) in EtOAc/toluene (300 μL + 300 μL) at room temperature. The crystal was first observed with naked eyes on the 6th day.

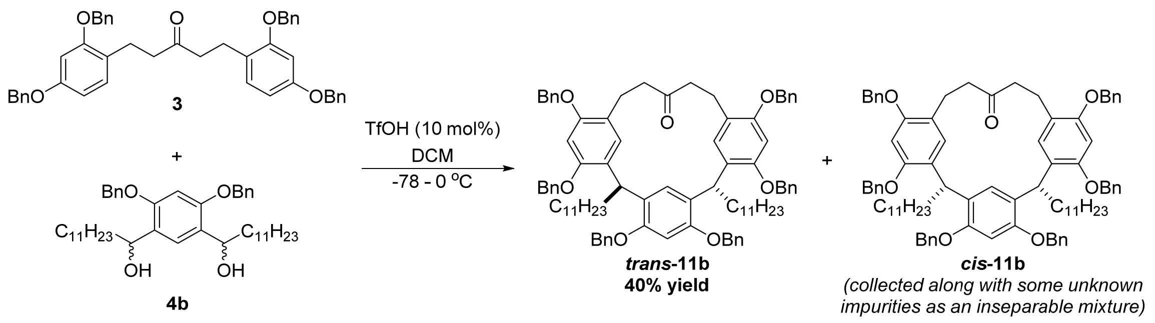

Scheme S4. Synthesis of benzyl-protected macrocycles ***trans*- & *cis*-11b**

In a 500 mL round-bottom flask, intermediate **3** (1.99 g, 3.00 mmol) and **4b** (2.37 g, 3.60 mmol) were dissolved into 300 mL DCM. After cooling to -78 °C with a dry-ice cooling bath, trifluoromethanesulfonic acid (26.5 μL, 300 μmol) was added dropwise with 2.00 mL DCM. The reaction was gradually warmed to 0°C in 3 hours. After the total consumption of material **3** (monitored by TLC), Et_3N (100 μL) was added to quench the reaction. DCM was evaporated on a rotavap to afford the crude product as brown oil. The *trans*-isomer (***trans*-11b**) was separated by column chromatography in 40% yield as a colorless oil.

The fractions that contained ***cis*-11b** were combined, along with some inseparable unknown impurities. This mixture was collected and used in the synthesis of ***cis*-2b** without further purification (see section 2.4).

(Note: the reactions should not be allowed to prolong or reach a higher temperature. Otherwise, the yield and trans/cis ratio of the desired products will decrease due to the acid-promoted epimerization and elimination, and the dehydrated byproduct was formed, ***see section 2.2.1****.)*

***trans*-1^4,1^6,3^4,3^6,5^4,5^6-hexakis(benzyloxy)-2,4-diundecyl-1,3,5(1,3)-tribenzenacyclodecaphan-8-one (*trans*-11b)**

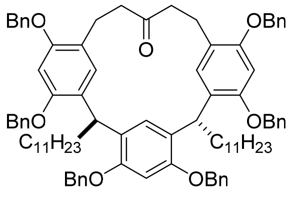

Chemical Formula: $C_{89}H_{104}O_7$
Molecular Weight: 1285.8040

Colorless oil, 40% yield (*trans*-isomer);

^{1}H NMR (500 MHz, $CDCl_3$) δ (ppm) 7.48 – 7.41 (m, 4H), 7.41 – 7.27 (m, 16H), 7.19 – 7.11 (m, 6H), 7.07 – 6.99 (m, 4H), 6.96 (s, 2H), 6.48 (s, 2H), 6.40 (s, 1H), 6.23 (s, 1H), 5.08 – 4.98 (m, 4H), 4.86 (d, J = 12.0 Hz, 2H), 4.84 – 4.78 (m, 4H), 4.74 (t, J = 7.6 Hz, 2H), 4.63 (d, J = 11.8 Hz, 2H), 3.19 (ddd, J = 14.5, 7.9, 4.3 Hz, 2H), 2.84 (ddd, J = 17.2, 8.4, 4.3 Hz, 2H), 2.70 (dddd, J = 29.8, 14.5, 8.2, 4.2 Hz, 4H), 1.71 (td, J = 8.5, 4.0 Hz, 2H), 1.63 – 1.56 (m, 2H), 1.28 – 1.09 (m, 36H), 0.87 (t, J = 7.0 Hz, 6H).

^{13}C NMR (125 MHz, $CDCl_3$) δ (ppm) 210.0, 155.5, 154.9, 154.4, 138.1, 137.5, 137.5, 128.5, 128.4, 128.3, 128.1, 127.7, 127.3, 127.3, 127.3, 127.2, 126.9, 125.9, 121.4, 99.3, 97.8, 70.6, 70.4, 70.2, 42.4, 35.1, 34.7, 31.9, 29.8, 29.7, 29.7, 29.7, 29.4, 28.0, 23.3, 22.7, 14.1.

ESI-MS for: $C_{89}H_{104}O_7Na$ $[M+Na]^+$: calcd 1307.77, found 1308.55;

HRMS (ESI) for: $C_{89}H_{104}O_7Na$ $[M+Na]^+$: calcd 1307.7674, found 1307.7655.

2.3 Synthesis of *trans*-window[1]resorcin[3]arene

2.3.1 Optimization of the global debenzylation

The optimization of the global debenzylation was performed with benzyl-protected ***trans*-11b** with undecyl-feet. The results were summarized in the following table.

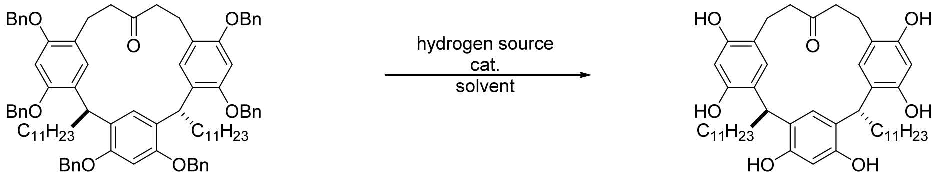

Entry	Solvent	Hydrogen	Cat.	Loading	Temp.	Yield (%)[b]
1	MeOH	H_2	Pd/C (5% w/w)	10 mg	25	n.d.[d]
2	MeOH	H_2	Pd/C (5% w/w)	20 mg	25	n.d.[d]
3	MeOH	H_2	Pd/C (5% w/w)	20 mg	50	n.d.[d]
4	wet MeOH[c]	H_2	Pd/C (5% w/w)	20 mg	50	n.d.[d]
5	wet EtOAc[c]	H_2	Pd/C (5% w/w)	20 mg	50	n.d.[d]
6	wet THF[c]	H_2	Pd/C (5% w/w)	20 mg	50	n.d.[d]
7	MeOH	HCO_2NH_4	Pd/C (5% w/w)	20 mg	reflux	34%
8	MeOH	H_2	Raney Ni	20 mg	25	n.d.[d]
9	MeOH	H_2	$Pd(OH)_2$/C (10%	20 mg	25	80%
10	MeOH	H_2	$Pd(OH)_2$/C (10%	10 mg	25	52%
11	MeOH	H_2	$Pd(OH)_2$/C (10%	5 mg	25	n.d.[d]
12	MeOH/THF (2:1)	H_2	$Pd(OH)_2$/C (10%	20 mg	25	93%
13	MeOH	HCO_2NH_4	$Pd(OH)_2$/C (10%	10 mg	reflux	68%

[a]Unless noted otherwise, reactions were performed with ***trans*-11b** (20.0 μmol, 1.00 equiv.), triple-layer hydrogen balloon, or HCO_2NH_4 (800 μmol, 40.0 eq.) as reductant, solvent (2.00 mL) for 24 hours at indicated temperatures. [b]Isolated yield. [c]10.0 μL of deionized water was added. [d]Complex reaction mixture was observed due to partial debenzylation.

(Note: by testing $Pd(OH)_2$/C from different chemical suppliers, we found that the reaction performances vary between the Pd sources. To ensure the good reproducibility of the global debenzylation, here we suggest the weight of $Pd(OH)_2$/C should not be lower than 70% of the weight of the material.)

2.3.2 Synthetic procedures and characterization

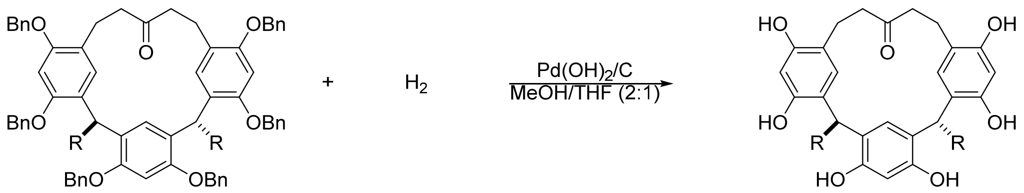

***trans*-11a:** R = *i*-Bu
***trans*-11b:** R = $C_{11}H_{23}$

***trans*-2a:** R = *i*-Bu
***trans*-2b:** R = $C_{11}H_{23}$

Scheme S5. Synthesis of *trans*-**2a&2b**

$Pd(OH)_2/C$ (300 mg) and Bn-protected intermediate ***trans*-11a** (545 mg, 500 umol) were mixed in a solvent mixture of MeOH/THF (80 mL / 40 mL) in a 100 mL two-neck flask. The mixture was stirred for 5 min, and then H_2 was charged (triple-layer balloon). The reaction was stirred at room temperature overnight. After the full conversion of material ***trans*-11a** (monitored by TLC), the mixture was carefully filtered through a short Celite pad and washed three times with DCM. The combined solution was concentrated on a rotavap. The crude product was purified by column chromatography to afford compound ***trans*-2a** in 89% yield (244 mg).

***trans*-1^4,1^6,3^4,3^6,5^4,5^6-hexahydroxy-2,4-diisobutyl-1,3,5(1,3)-tribenzenacyclodecaphan-8-one (*trans*-2a)**

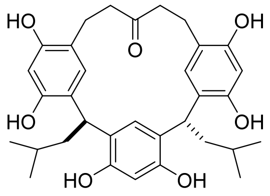

Chemical Formula: $C_{33}H_{40}O_7$
Molecular Weight: 548.6760

Yellowish foam, 89% yield;

^{1}H NMR (500 MHz, Acetone-D6) δ (ppm) 8.59 – 7.77 (br, 6H), 7.01 (s, 2H), 6.54 (s, 1H), 6.36 (s, 1H), 6.31 (s, 2H), 4.38 (t, J = 8.0 Hz, 2H), 2.95 – 2.90 (m, 2H), 2.81 (dd, J = 6.6, 4.3 Hz, 4H), 2.76 – 2.72 (m, 2H), 1.89 (dt, J = 14.2, 7.4 Hz, 2H), 1.72 – 1.67 (m, 2H), 1.44 (dt, J = 13.4, 6.7 Hz, 2H), 0.87 (dd, J = 6.7, 3.9 Hz, 12H).

^{13}C NMR (125 MHz, Acetone-D6) δ (ppm) 209.5, 154.3, 154.0, 152.8, 130.0, 129.7, 124.7, 122.3, 120.1, 103.6, 103.5, 42.8, 42.6, 26.6, 23.1, 22.8, 22.7.

ESI-MS for: $C_{33}H_{39}O_7$ $[M-H]^-$: calcd 547.27, found 547.45;

HRMS (ESI) for: $C_{33}H_{40}O_7Na$ $[M+Na]^+$: calcd 571.2666, found 571.2660.

Compound ***trans*-2b** was synthesized following the same procedure. The titled compound was isolated in 92% yield.

***trans*-1^4,1^6,3^4,3^6,5^4,5^6-hexahydroxy-2,4-diundecyl-1,3,5(1,3)-tribenzenacyclodecaphan-8-one (*trans*-2b)**

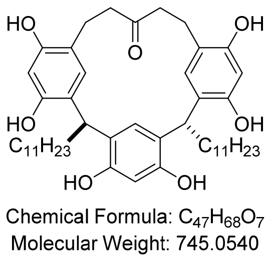

Yellowish foam, 92% yield;

^{1}H NMR (500 MHz, Acetone-D6) δ (ppm) 8.78 – 7.16 (br, 6H), 7.02 (s, 2H), 6.58 (s, 1H), 6.35 (s, 1H), 6.28 (s, 2H), 4.22 (t, *J* = 7.8 Hz, 2H), 2.93 – 2.87 (m, 2H), 2.75 (ddd, *J* = 18.9, 11.0, 5.2 Hz, 6H), 1.99 (ddt, *J* = 15.0, 8.1, 4.0 Hz, 2H), 1.86 (dtd, *J* = 13.1, 8.9, 4.8 Hz, 2H), 1.26 (d, *J* = 7.1 Hz, 36H), 0.87 (t, *J* = 6.7 Hz, 6H).

^{13}C NMR (125 MHz, Acetone-D6) δ (ppm) 209.5, 154.3, 154.0, 152.9, 130.2, 129.6, 124.8, 122.4, 120.2, 103.8, 103.6, 42.7, 38.7, 33.7, 32.7, 31.8, 30.4, 30.3, 30.2, 30.1, 28.9, 23.3, 22.7, 14.4.

ESI-MS for: $C_{47}H_{69}O_7$ [M+H]$^+$: calcd 745.50, found 745.55;

HRMS (ESI) for: $C_{47}H_{68}O_7Na$ [M+Na]$^+$: calcd 767.4857, found 767.4847.

Mp. 216 °C

2.4 Synthesis of *cis*-window[1]resorcin[3]arene

2.4.1 Acid-promoted *trans*- to *cis*-epimerization

Gold-catalyzed epimerization from *trans*-2b to *cis*-2b

We were pleased to see the compound ***trans*-2b** with undecyl-feet easily isomerized to its *cis*-isomer in $CDCl_3$ in the presence of 10 mol% of IPrAuOTf[3]. The compound ***cis*-2b** was isolated in almost quantitative yield.

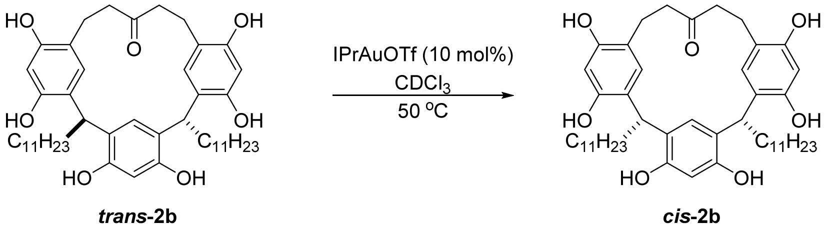

Scheme S6. Isomerization procedure from ***trans*-2b** to ***cis*-2b.**

Procedure: Compound ***trans*-2b** (149 mg, 200 μmol) was dissolved into 10 mL $CDCl_3$. The mixture was stirred at room temperature for 15 min. After that, IPrAuOTf (14.7 mg, 20.0 μmol) was added and the mixture was heated to 50 °C for 8 hours. The white precipitation was observed while the reaction proceeded. After cooling to room temperature, the white precipitation was collected by filtration and washed carefully with cooled $CHCl_3$. The collected product was dried under a high vacuum to afford ***cis*-2b** in 99% yield (149 mg) as a white foamy solid.

cis-$1^4,1^6,3^4,3^6,5^4,5^6$-hexahydroxy-2,4-diundecyl-1,3,5(1,3)-tribenzenacyclodecaphan-8-one (*cis*-2b)

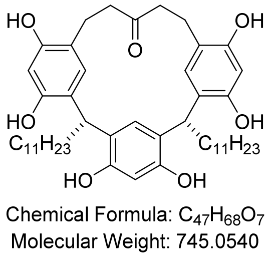

White foamy solid, 99% yield;

^{1}H NMR (500 MHz, acetone-D6) δ (ppm) 8.66 (s, 2H), 8.11 (s, 2H), 7.93 (s, 2H), 7.38 (s, 2H), 7.17 (s, 1H), 6.42 (s, 1H), 6.19 (s, 2H), 4.31 (dd, J = 9.9, 5.8 Hz, 2H), 2.85 – 2.58 (m, 8H), 2.35 – 2.28 (m, 2H), 2.08 – 2.03 (m, 2H), 1.46 – 1.30 (m, 36H), 0.96 – 0.89 (m, 6H).

^{13}C NMR (125 MHz, acetone-D6) δ (ppm) 209.9, 155.0, 154.2, 153.2, 130.5, 127.3, 125.9, 124.1, 121.6, 104.5, 103.9, 43.6, 35.6, 34.8, 33.4, 31.3, 31.2, 31.2, 29.6, 24.1, 24.0, 15.1.

ESI-MS for: $C_{47}H_{67}O_7$ [M-H]$^-$: calcd 743.50, found 743.40;

HRMS (ESI) for: $C_{47}H_{68}O_7Na$ $[M+Na]^+$: calcd 767.4857, found 767.4844. **Mp**. 252 °C (decomp.)

However, this gold-catalyzed epimerization process didn't work with ***trans*-2a**. Thus, several acid promoters were screened for the *'trans- to cis-'* epimerization of ***trans*-2a**. The results were summarized in the following table:

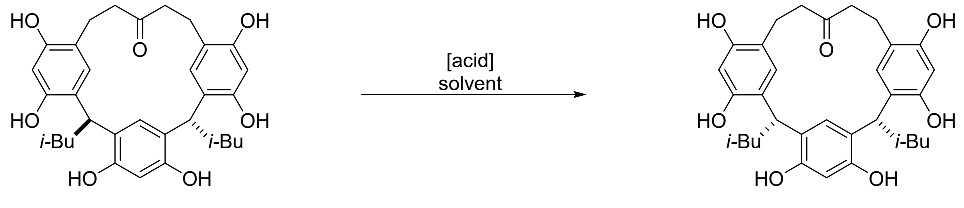

Entry[a]	Acid	Solvent	Observations
1	IPrAuOTf	acetone	no conversion
2	IPrAuOTf	$CDCl_3$	no conversion
3	IPrAuOTf	THF	no conversion
4	$FeCl_3$	acetone	material decomposed
5	$FeCl_3$	DCM	material decomposed
6	$AlCl_3$	acetone	material decomposed
7	$AlCl_3$	DCM	red messy mixture
8	$Sc(OTf)_3$	acetone	46%, 9:1 (*cis/trans*)[b]
9	$Sc(OTf)_3$	DCM	material decomposed
10[c]	$Sc(OTf)_3$	THF (reflux)	70% 10:1 (*cis/trans*)[b]
11	$Zn(OTf)_2$	acetone	no conversion
12	TsOH	acetone	major decomposition
13	PPTS	acetone	no conversion
14	Amberlyst 15	acetone	no conversion at r.t., decomposed at reflux
15	TfOH	acetone	major decomposition

[a]Unless noted otherwise, reactions were performed with ***trans*-2a** (5.48 mg, 10.0 μmol, 1.0 equiv.), Lewis acid or Bronsted acid (2.00 μmol, 0.2 equiv.), in solvents (1.00 mL) for 24 hours at 25 °C; [b]Isolated yield; [c]Reaction time: 8 h.

Scandium-catalyzed epimerization from *trans*-2a to *cis*-2a

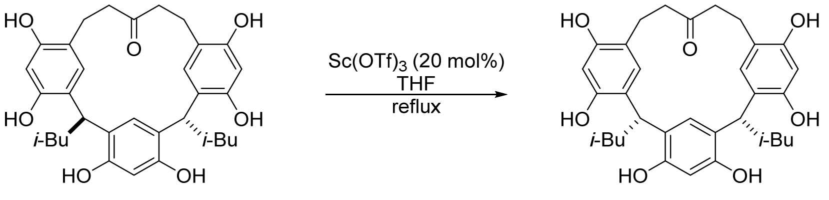

Scheme S7. Isomerization process from ***trans*-2a** to ***cis*-2a**

Procedure: Compound ***trans*-2a** (54.8 mg, 100 μmol) was dissolved into 5.00 mL THF. The mixture was stirred at room temperature for 15 min. After that, $Sc(OTf)_3$ (9.84 mg, 20.0 μmol) was added and the yellowish solution was degassed by a 'freeze/unfreeze' operation 3 times with a liquid nitrogen bath. The mixture was slowly warmed to room temperature, and then heated to reflux overnight. After the reaction completion, the *cis*-isomer was separated by column chromatography to afford ***cis*-2a** in 70% yield (36.2 mg) as a white solid.

***cis*-1^4,1^6,3^4,3^6,5^4,5^6-hexahydroxy-2,4-diisobutyl-1,3,5(1,3)-tribenzenacyclodecaphan-8-one (*cis*-2a)**

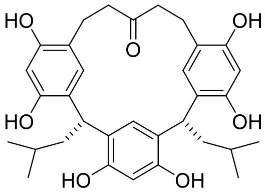

Chemical Formula: $C_{33}H_{40}O_7$
Molecular Weight: 548.6760

Yellowish solid, 70% yield;

^{1}H NMR (500 MHz, Acetone-D6) δ (ppm) 8.64 (s, 2H), 8.05 (s, 2H), 7.87 (s, 2H), 7.40 (s, 2H), 7.18 (s, 1H), 6.43 (s, 1H), 6.20 (s, 2H), 4.47 (dd, J = 10.5, 5.6 Hz, 2H), 2.85 (ddd, J = 12.0, 10.4, 1.9 Hz, 2H), 2.79 – 2.68 (m, 4H), 2.65 – 2.57 (m, 2H), 2.34 (ddd, J = 13.2, 10.5, 5.1 Hz, 2H), 1.82 (ddd, J = 13.2, 8.7, 5.6 Hz, 2H), 1.52 – 1.45 (m, 2H), 0.99 (dd, J = 6.7, 4.8 Hz, 12H).

^{13}C NMR (125 MHz, Acetone-D6) δ (ppm) 209.9, 155.0, 154.2, 153.0, 130.6, 127.9, 125.8, 124.0, 121.6, 104.5, 103.9, 44.7, 43.6, 32.2, 27.4, 24.6, 24.1, 23.1.

ESI-MS for: $C_{33}H_{40}O_7Na$ $[M+Na]^+$: calcd 571.27, found 571.30;

HRMS (ESI) for: $C_{33}H_{40}O_7Na$ $[M+Na]^+$: calcd 571.2666, found 571.2659

2.4.2 Synthesis of *cis*-window[1]resorcin[3]arene with global-benzylated intermediates

In the above-mentioned macrocyclization step, the benzyl-protected *cis*-isomers were collected as the minor epimers along with unknown impurities (see section 2.2 for details). Although these two benzyl-protected *cis*-window[1]resorcin[3]arenes couldn't be fully purified, they were used directly in the Pd-catalyzed hydrogenolysis process. The debenzylated products ***cis*-2a** & ***cis*-2b** could be obtained.

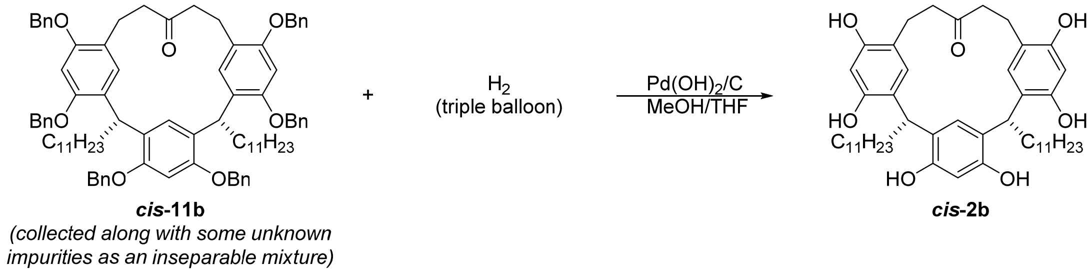

Scheme S8. Synthesis of ***cis*-2b** from ***cis*-11b**

Procedure: $Pd(OH)_2/C$ (100 mg) and ***cis*-11b** (193 mg, mixed with some impurities) were mixed in a solvent mixture of MeOH/THF (20 ml/10 ml) in a 100 mL two-neck flask. The mixture was stirred for 5 min, and then H_2 was charged (triple-layer balloon). The reaction was stirred at room temperature overnight. After the full conversion of the material (monitored by TLC), H_2 was released. The mixture was carefully filtered through a short Celite pad and washed 3 times with DCM. The combined solution was concentrated on a rotavap. The crude product was purified by column chromatography to afford compound ***cis*-2b** as an off-white powder (96 mg).

Based on the amount of **3** and **4b** used in the last step, the overall yield in the two steps was calculated as 17%.

This overall yield varies from 16% to 19% in several batches of synthesis, presumably due to the uncertain amount of the inseparable impurities contained in the material.

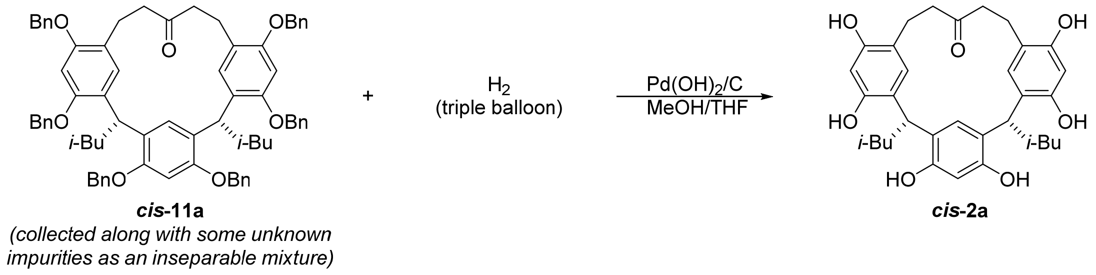

Scheme S9. Synthesis of ***cis*-2a** from ***cis*-11a**

Procedure: Pd(OH)$_2$/C (100 mg) and ***cis*-11a** (163 mg, mixed with some impurities and a trace amount of ***trans*-11a**) were mixed in a solvent mixture of MeOH/THF (20 ml/10 ml) in a 100 mL two-neck flask. The mixture was stirred for 5 min, and then H_2 was charged (triple-layer balloon). The reaction was stirred at room temperature overnight. After the full conversion of the material (monitored by TLC), H_2 was released. The mixture was carefully filtered through a short Celite pad and washed 3 times with DCM. The combined solution was concentrated on a rotavap. The crude product was purified by column chromatography to afford compound ***cis*-2a** as a white powder (57 mg).

Based on the amount of **3** and **4a** used in the last step, the overall yield in the two steps was calculated as 14%.

This overall yield varies from 11% to 16% in several batches of synthesis, presumably due to the uncertain amount of the inseparable impurities contained in the material.

2.5 Comparison of NOESY NMR spectra

Besides X-ray crystallography structures (see the following section), NOESY experiments provide additional evidence to distinguish the conformations of *cis*- & *trans*-isomers.

As shown below, on the NOESY spectra of ***trans*-2b**, NOE interactions were detected between the benzylic protons H_1 and lower rim protons H_2/H_3. In ***cis*-2b**, no NOE was detected between those protons as both two benzylic protons are pointing outward of the cavity.

Full sets of characterizations of these two compounds are shown in section 8.

***cis*-2b** in acetone-D6

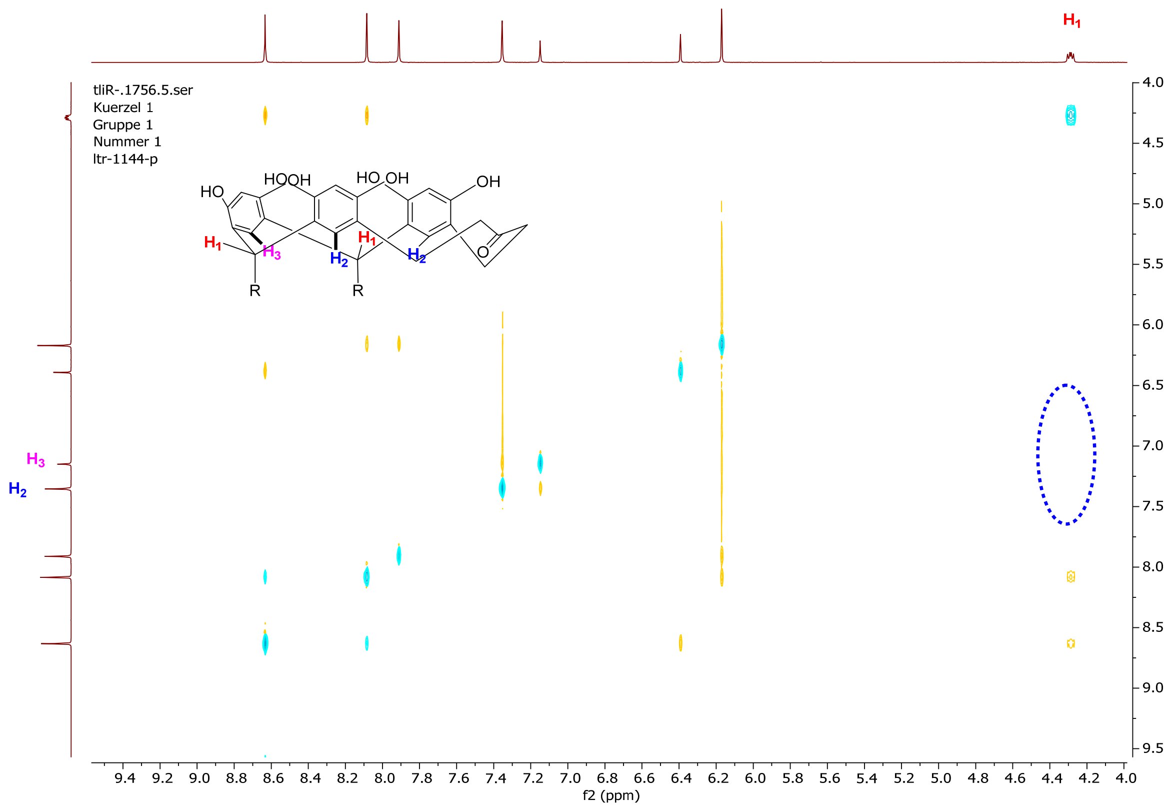

***trans*-2b** in acetone-D6

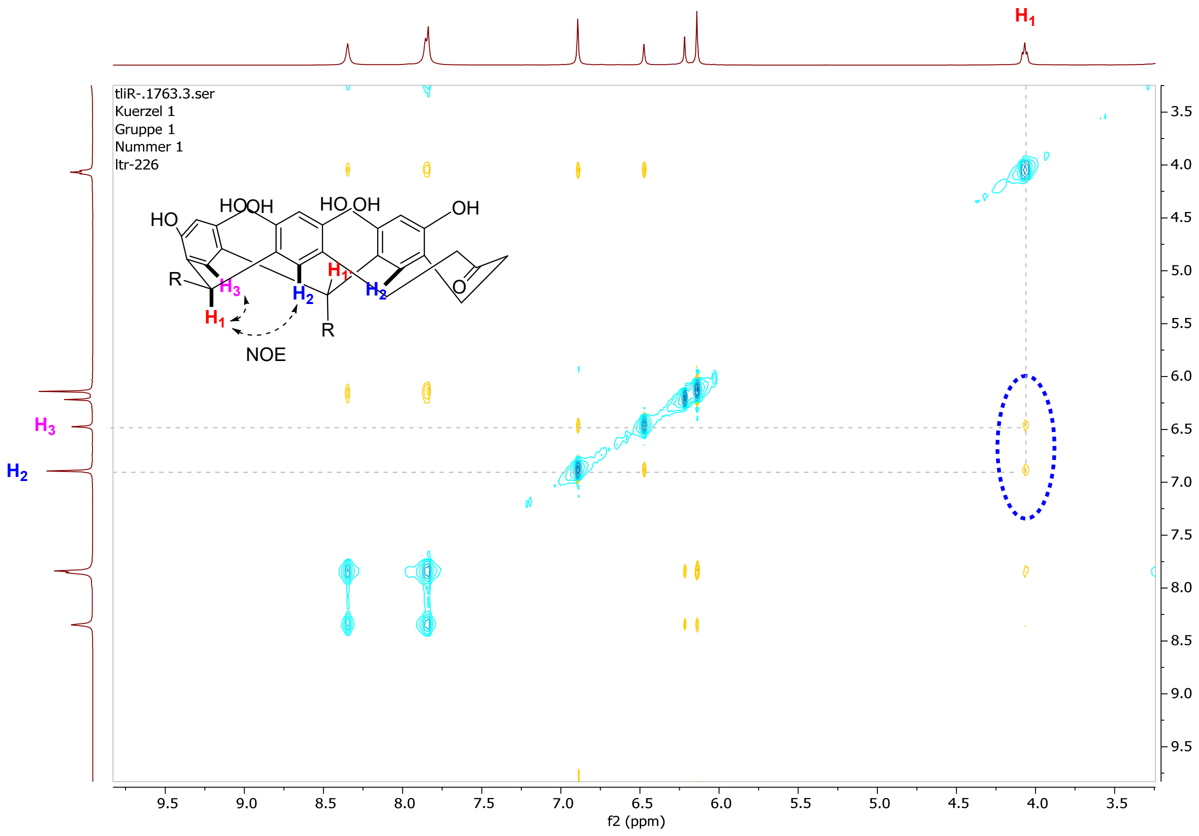

3. Crystallographic Structures and X-Ray Data

3.1 Crystallographic structures

***trans*-2a** (global benzyl protected)

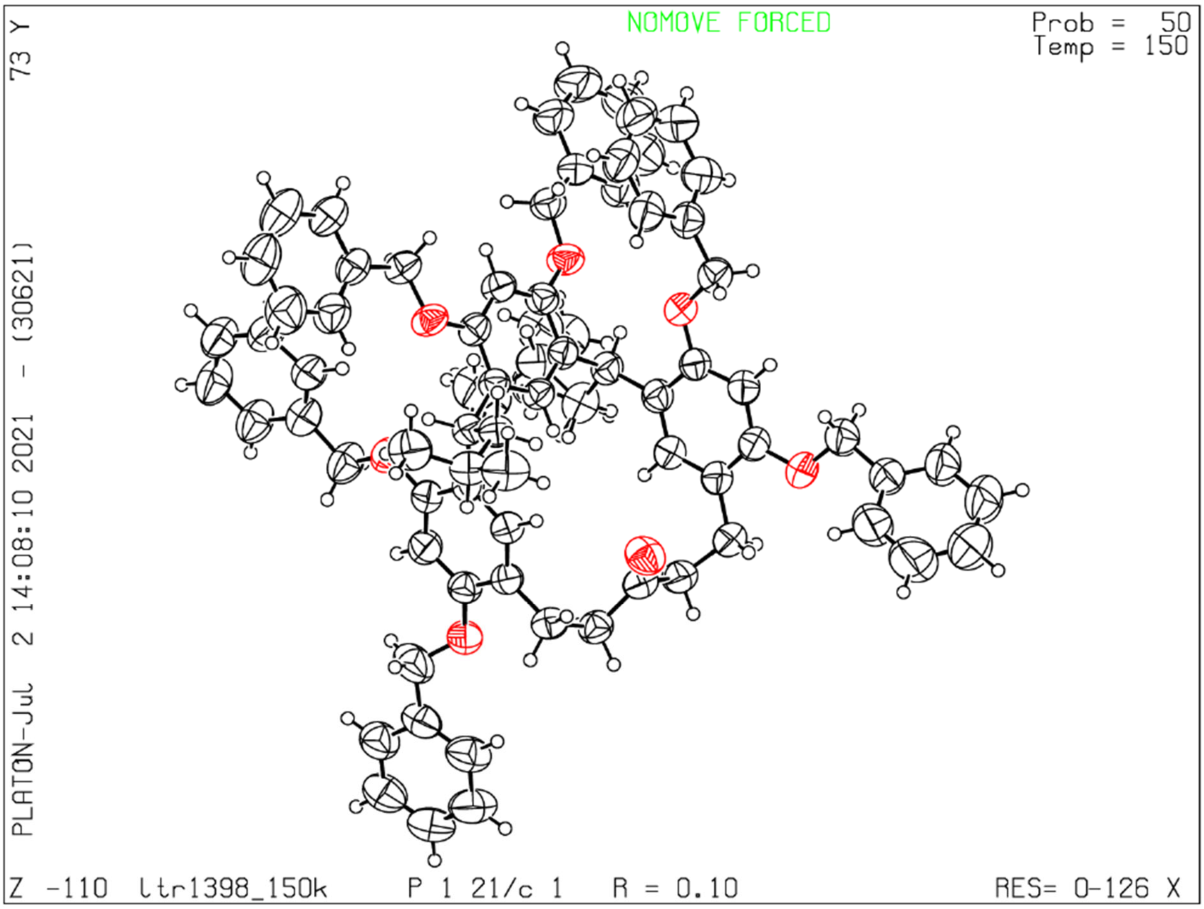

***cis*-2a** (global ferrocenyl protected)

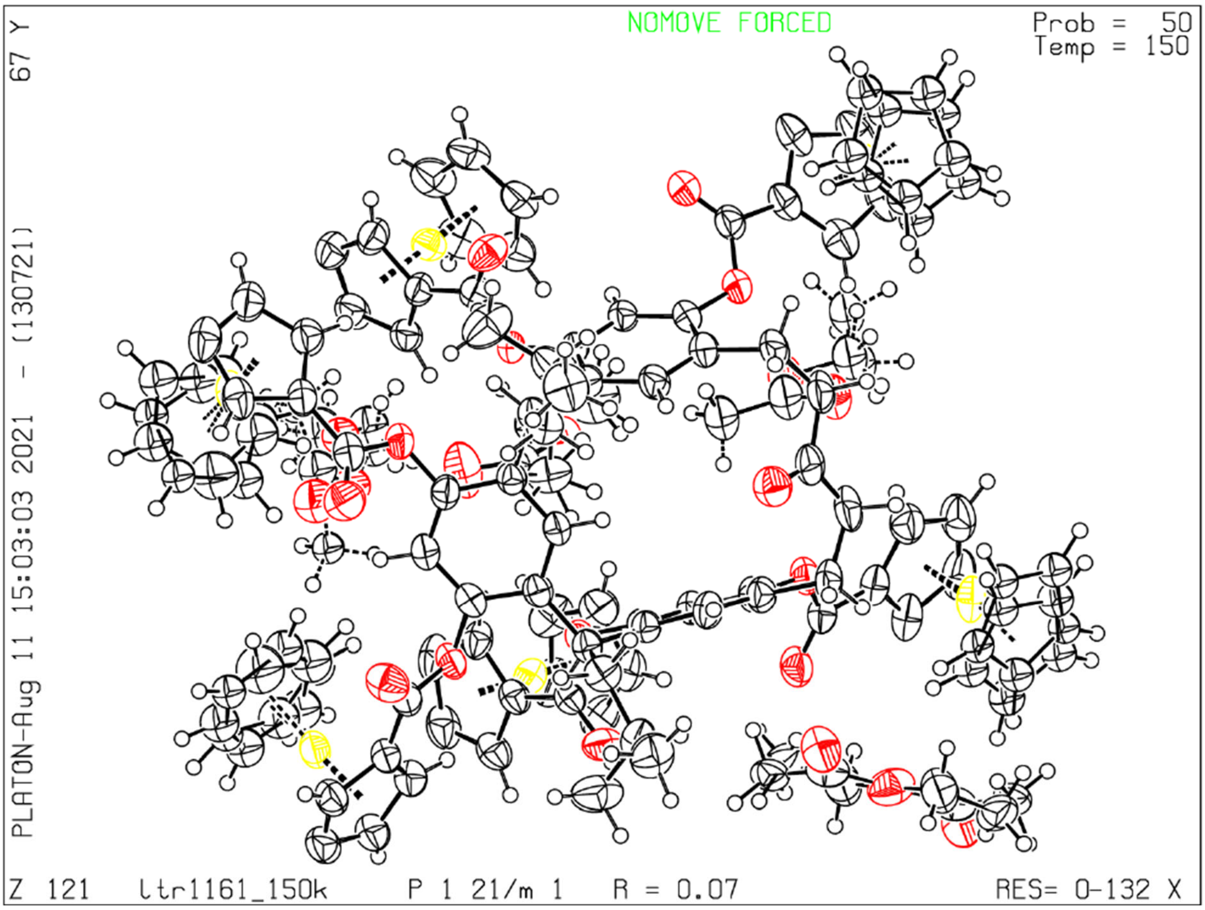

3.2 Preparation of the global ferrocenyl-protected derivative

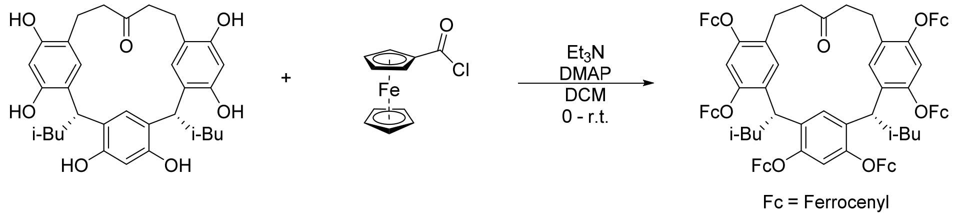

***cis*-2a** **S1**

Scheme S10. Global protection of ***cis*-2a** with ferrocenyl chloride.

Procedure: ***cis*-2a** (100 μmol, 54.8 mg) was weighed into a 25 mL flame-dried flask and 5.0 mL DCM was added. The solution was cooled to 0 °C with an ice-water cooling bath. Et_3N (279 μL, 2.00 mmol) was added followed by DMAP (6.11 mg, 50.0 μmol). After stirring for 10 min, a solution of ferrocenyl chloride[4] (348 mg, 1.40 mmol) in 5.00 mL DCM was added dropwise. The reaction was slowly warmed to room temperature and continued stirred for 20 hours before being quenched with $NaHCO_3$ (saturated aq.). The product was extracted with 4 x 5.0 mL DCM. The combined organic solution was washed with 10.0 mL brine, dried over anhydrous. Na_2SO_4, and concentrated on a rotavap. The crude product was purified by column chromatography. The resulting red slime, which contains some impurities, was then triturated and sonicated with 5.0 mL MeOH. The resulting orange solid was filtered, washed with 10 mL MeOH, and dried by sucking air through the filter cake for 3 hours. The pure product **S1** was collected (130 mg, 71% yield).

***cis*-2,4-diisobutyl-8-oxo-1,3,5(1,3)-tribenzenacyclodecaphane-1^4,1^6,3^4,3^6,5^4,5^6-hexayl hexaferrocenecarboxylate (S1)**

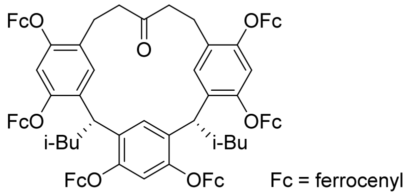

Chemical Formula: $C_{99}H_{90}Fe_6O_{13}$
Molecular Weight: 1820.85

Orange solid, 71% yield;

^{1}H NMR (500 MHz, $CDCl_3$) δ (ppm) 7.57 (s, 2H), 7.21 (s, 1H), 7.11 (s, 3H), 5.05 (ddt, J = 8.2, 2.6, 1.4 Hz, 4H), 4.96 (dt, J = 2.6, 1.3 Hz, 2H), 4.91 (dt, J = 2.6, 1.3 Hz, 2H), 4.81 (dt, J = 2.6, 1.3 Hz, 2H), 4.70 (dt, J = 2.6, 1.3 Hz, 2H), 4.64 (qq, J = 3.9, 2.6, 2.0 Hz, 10H), 4.59 (dt, J = 2.6, 1.3 Hz, 2H), 4.54 (td, J = 2.6, 1.4 Hz, 2H), 4.40 (s, 10H), 4.35 (s, 10H), 4.29 (s, 10H), 3.12 – 3.01 (m, 8H), 2.01 – 1.95 (m, 2H), 1.84 (dt, J = 14.2, 7.2 Hz, 2H), 1.64 (dt, J = 13.4, 6.6 Hz, 2H), 0.89 (d, J = 6.6 Hz, 6H), 0.81 (d, J = 6.6 Hz, 6H).

^{13}**C NMR** (125 MHz, $CDCl_3$) δ (ppm) 209.8, 171.2, 171.1, 170.8, 149.4, 149.2, 148.6, 135.7, 134.2, 131.8, 131.6, 129.0, 118.5, 118.4, 73.7, 73.7, 73.6, 73.6, 73.6, 72.9, 72.7, 72.3, 72.2, 72.1, 72.0, 72.0, 71.9, 71.8, 71.6, 71.6, 71.5, 71.5, 71.4, 71.3, 50.6, 45.8, 43.1, 35.8, 27.2, 25.5, 24.1, 23.7, 23.5, 15.0.

ESI-MS for: $C_{99}H_{88}Fe_6O_{13}Na$ $[M+Na]^+$: calcd 1843.22, found 1844.10;

HRMS (ESI) for: $C_{99}H_{88}Fe_6O_{13}Na$ $[M+Na]^+$: calcd 1843.2220, found 1843.2237.

The single-crystal sample that is suitable for structure resolution was obtained by slow evaporation of a solution of **S1** (3.00 mg) in EtOAc/heptane (400 μL + 200 μL) at room temperature. The crystal was first observed with naked eyes on the 4th day.

3.3 X-ray data

3.3.1 X-ray data of the *trans*-isomer

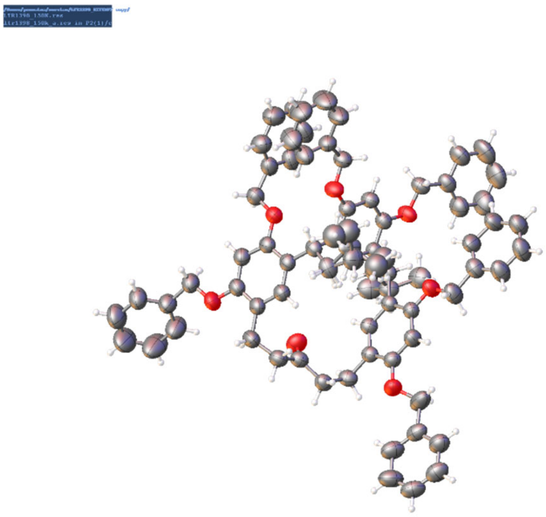

Compound	LTR1398_150K
Formula	$C_{79.9}H_{81.6}O_7$
$D_{calc.}$/ g cm^{-3}	1.153
μ/mm^{-1}	0.359
Formula Weight	1153.85
Colour	colourless
Shape	block-shaped
Size/mm^3	0.22×0.17×0.13
T/K	150
Crystal System	monoclinic
Space Group	$P2_1/c$
a/Å	9.7121(3)
b/Å	35.1657(10)
c/Å	19.9152(6)
α/°	90
β/°	102.349(2)
γ/°	90
V/Å^3	6644.3(3)
Z	4
Z'	1
Wavelength/Å	1.34143
Radiation type	GaK_α
Θ_{min}/°	2.186
Θ_{max}/°	55.289
Measured Refl's.	41164
Indep't Refl's	12471
Refl's I≥2 σ(I)	9097
R_{int}	0.0589
Parameters	749
Restraints	18
Largest Peak	0.314
Deepest Hole	-0.418
GooF	0.987
wR_2 (all data)	0.3873
wR_2	0.3253
R_1 (all data)	0.1170
R_1	0.0994

Experimental. Single colourless block-shaped crystals of **LTR1398_150K** were used as supplied. A suitable crystal with dimensions 0.22 × 0.17 × 0.13 mm^3 was selected and The crystal was mounted on a mylar loop in perfluoroether oil on a STOE STADIVARI diffractometer. The crystal was kept at a steady T = 150 K during data collection. The structure was solved with the **ShelXT** 2018/2 (Sheldrick, 2018) solution program using dual methods and by using **Olex2** 1.3 (Dolomanov et al., 2009) as the graphical interface. The model was refined with **ShelXL** 2018/3 (Sheldrick, 2015) using full matrix least squares minimisation on F^2.

Crystal Data. $C_{79.9}H_{81.6}O_7$, M_r = 1153.85, monoclinic, $P2_1/c$ (No. 14), a = 9.7121(3) Å, b = 35.1657(10) Å, c = 19.9152(6) Å, β = 102.349(2)°, $\alpha = \gamma$ = 90°, V = 6644.3(3) Å^3, T = 150 K, Z = 4, Z' = 1, μ(GaK_α) = 0.359, 41164 reflections measured, 12471 unique (R_{int} = 0.0589) which were used in all calculations. The final wR_2 was 0.3873 (all data) and R_1 was 0.0994 (I≥2 σ(I)).

Structure Quality Indicators

**Reflections:**	d min (Ga) 2θ=110.6° 0.82	I/σ(I) 21.5	Rint 5.89%	Full 107.2° 97% to 110.6° 99.2
**Refinement:**	Shift 0.000	Max Peak 0.3	Min Peak -0.4	GooF 0.987

A colourless block-shaped-shaped crystal with dimensions 0.22 × 0.17 × 0.13 mm^3 was mounted on a mylar loop in perfluoroether oil. Data were collected using a STOE STADIVARI diffractometer equipped with an Oxford Cryosystems low-temperature device operating at T = 150 K.

Data were measured using rotation method, ω scans using GaK_α radiation. The diffraction pattern was indexed and the total number of runs and images was based on the strategy calculation from the program X-Area Pilatus3_SV 1.31.154.0 (STOE, 2019). The maximum resolution that was achieved was Θ = 55.289° (0.82 Å).

The unit cell was refined using X-Area Integrate 1.77.0.0 (STOE, 2019)X-Area LANA 1.77.1.0 (STOE, 2019)X-Area X-Red32 1.65.0.0 (STOE, 2018) on 55574 reflections, 135% of the observed reflections.

Data reduction, scaling and absorption corrections were performed using X-Area Integrate 1.77.0.0 (STOE, 2019)X-Area LANA 1.77.1.0 (STOE, 2019)X-Area X-Red32 1.65.0.0 (STOE, 2018). The final completeness is 99.20 % out to 55.289° in Θ. A multi-scan absorption correction was performed using STOE X-Red32, absorption correction by Gaussian integration, analogous to P. Coppens in: F. R. Ahmed (Editor), "Crystallographic Computing", Munksgaard, Copenhagen (1970), 255 - 270. Afterwards scaling of reflection intensities was performed within STOE LANA. J. Koziskova, F. Hahn, J. Richter, J. Kozisek, Acta Chimica Slovaca, vol. 9, no. 2, 2016, pp. 136 - 140. Finally a spherical absorption correction was done within STOE LANA.. The absorption coefficient μ of this material is 0.359 mm^{-1} at this wavelength (λ = 1.34143Å) and the minimum and maximum transmissions are 0.287 and 0.644.

The structure was solved and the space group $P2_1/c$ (# 14) determined by the ShelXT 2018/2 (Sheldrick, 2018) structure solution program using dual methods and refined by full matrix least squares minimisation on $\boldsymbol{F^2}$ using version 2018/3 of **ShelXL** 2018/3 (Sheldrick, 2015). All non-hydrogen atoms were refined anisotropically. Hydrogen atom positions were calculated geometrically and refined using the riding model. Hydrogen atom positions were calculated geometrically and refined using the riding model.

_refine_special_details: one isopropyle subsituent is disordered over 2 orientations with50-50 occupancies. SADI and EADP restraints were used with anisotropic refinement.A solvent mask was used to treat the solvent region and0.7 Tolune were found. All the formulae and numebers take thisresult into account.

_exptl_absorpt_process_details: STOE X-Red32, absorption correction by Gaussian integration, analogous toP. Coppens in: F. R. Ahmed (Editor), "Crystallographic Computing", Munksgaard,Copenhagen (1970), 255 - 270. Afterwards scaling of reflection intensities wasperformed within STOE LANA. J. Koziskova, F. Hahn, J. Richter, J. Kozisek, ActaChimica Slovaca, vol. 9, no. 2, 2016, pp. 136 - 140.Finally a spherical absorption correction was done within STOE LANA.

There is a single molecule in the asymmetric unit, which is represented by the reported sum formula. In other words: Z is 4 and Z' is 1.

3.3.2 X-ray data of the *cis*-isomer

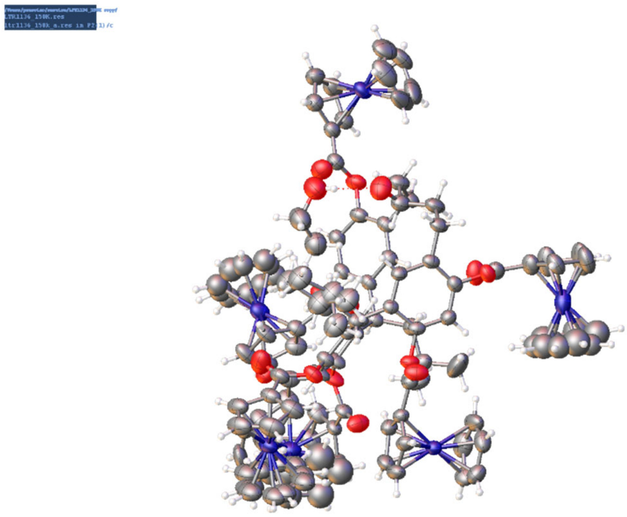

**Compound**	**LTR1136_150K**
Formula	$C_{103}Fe_6H_{101}O_{15}$
$D_{calc.}$/ g cm^{-3}	1.430
μ/mm^{-1}	5.647
Formula Weight	1913.93
Colour	red
Shape	block-shaped
Size/mm^3	0.20×0.15×0.11
T/K	150
Crystal System	monoclinic
Space Group	$P2_1/c$
a/Å	24.9648(7)
b/Å	16.1427(7)
c/Å	23.8243(8)
α/°	90
β/°	112.148(2)
γ/°	90
V/Å^3	8892.7(6)
Z	4
Z'	1
Wavelength/Å	1.34143
Radiation type	GaK$_\alpha$
Θ_{min}/°	1.662
Θ_{max}/°	55.297
Measured Refl's.	65504
Indep't Refl's	16558
Refl's I≥2 σ(I)	13218
$R_{\rm int}$	0.0415
Parameters	1055
Restraints	36
Largest Peak	1.353
Deepest Hole	-1.023
GooF	1.025
wR_2 (all data)	0.1764
wR_2	0.1611
R_1 (all data)	0.0819
R_1	0.0639

Experimental. Single red block-shaped crystals of **LTR1136_150K** were used as supplied. A suitable crystal with dimensions 0.20 × 0.15 × 0.11 mm^3 was selected and The crystal was mounted on a mylar loop in perfluoroether oil on a STOE STADIVARI diffractometer. The crystal was kept at a steady T = 150 K during data collection. The structure was solved with the **ShelXT** 2018/2 (Sheldrick, 2018) solution program using dual methods and by using **Olex2** 1.3 (Dolomanov et al., 2009) as the graphical interface. The model was refined with **ShelXL** 2018/3 (Sheldrick, 2015) using full matrix least squares minimisation on F^2.

Crystal Data. $C_{103}Fe_6H_{101}O_{15}$, M_r = 1913.93, monoclinic, $P2_1/c$ (No. 14), a = 24.9648(7) Å, b = 16.1427(7) Å, c = 23.8243(8) Å, β = 112.148(2)°, $\alpha = \gamma$ = 90°, V = 8892.7(6) Å^3, T = 150 K, Z = 4, Z' = 1, μ(GaK$_\alpha$) = 5.647, 65504 reflections measured, 16558 unique ($R_{\rm int}$ = 0.0415) which were used in all calculations. The final wR_2 was 0.1764 (all data) and R_1 was 0.0639 (I≥2 σ(I)).

Structure Quality Indicators

**Reflections:**	d min (Ga) 2θ=110.6° 0.82	I/σ(I) 36.7	Rint 4.15%	Full 107.2° 96% to 110.6° 98.9
**Refinement:**	Shift -0.001	Max Peak 1.4	Min Peak -1.0	GooF 1.025

A red block-shaped-shaped crystal with dimensions 0.20 × 0.15 × 0.11 mm³ was mounted on a mylar loop in perfluoroether oil. Data were collected using a STOE STADIVARI diffractometer equipped with an Oxford Cryosystems low-temperature device operating at T = 150 K.

Data were measured using rotation method, ω scans using GaK_{α} radiation. The diffraction pattern was indexed and the total number of runs and images was based on the strategy calculation from the program X-Area Pilatus3_SV 1.31.154.0 (STOE, 2019). The maximum resolution that was achieved was Θ = 55.297° (0.82 Å).

The unit cell was refined using X-Area Integrate 1.77.0.0 (STOE, 2019)X-Area LANA 1.77.1.0 (STOE, 2019)X-Area X-Red32 1.65.0.0 (STOE, 2018) on 76408 reflections, 117% of the observed reflections.

Data reduction, scaling and absorption corrections were performed using X-Area Integrate 1.77.0.0 (STOE, 2019)X-Area LANA 1.77.1.0 (STOE, 2019)X-Area X-Red32 1.65.0.0 (STOE, 2018). The final completeness is 98.90 % out to 55.297° in Θ. A multi-scan absorption correction was performed using STOE X-Red32, absorption correction by Gaussian integration, analogous to P. Coppens in: F. R. Ahmed (Editor), "Crystallographic Computing", Munksgaard, Copenhagen (1970), 255 - 270. Afterwards scaling of reflection intensities was performed within STOE LANA. J. Koziskova, F. Hahn, J. Richter, J. Kozisek, Acta Chimica Slovaca, vol. 9, no. 2, 2016, pp. 136 - 140. Finally a spherical absorption correction was done within STOE LANA.. The absorption coefficient μ of this material is 5.647 mm^{-1} at this wavelength (λ = 1.34143Å) and the minimum and maximum transmissions are 0.104 and 0.221.

The structure was solved and the space group $P2_1/c$ (# 14) determined by the ShelXT 2018/2 (Sheldrick, 2018) structure solution program using dual methods and refined by full matrix least squares minimisation on $\boldsymbol{F^2}$ using version 2018/3 of **ShelXL** 2018/3 (Sheldrick, 2015). All non-hydrogen atoms were refined anisotropically. Hydrogen atom positions were calculated geometrically and refined using the riding model. Hydrogen atom positions were calculated geometrically and refined using the riding model.

_refine_special_details: Some Cp rings are orientationally disordered.Geometric restraints and isotropic refinement had to be used.One Ferrocene unit is disorderd over two orientations.Solvent mask had to be used to treat part of the solvent region:1EtOH per formula unit was found and added to all of the formulae and numbers.

_exptl_absorpt_process_details: STOE X-Red32, absorption correction by Gaussian integration, analogous toP. Coppens in: F. R. Ahmed (Editor), "Crystallographic Computing", Munksgaard,Copenhagen (1970), 255 - 270. Afterwards scaling of reflection intensities wasperformed within STOE LANA. J. Koziskova, F. Hahn, J. Richter, J. Kozisek, ActaChimica Slovaca, vol. 9, no. 2, 2016, pp. 136 - 140.Finally a spherical absorption correction was done within STOE LANA.

There is a single molecule in the asymmetric unit, which is represented by the reported sum formula. In other words: Z is 4 and Z' is 1.

4. Assembly Investigation

4.1 The comparison of ^{1}H NMR spectra of *trans*-2b in difference solvents

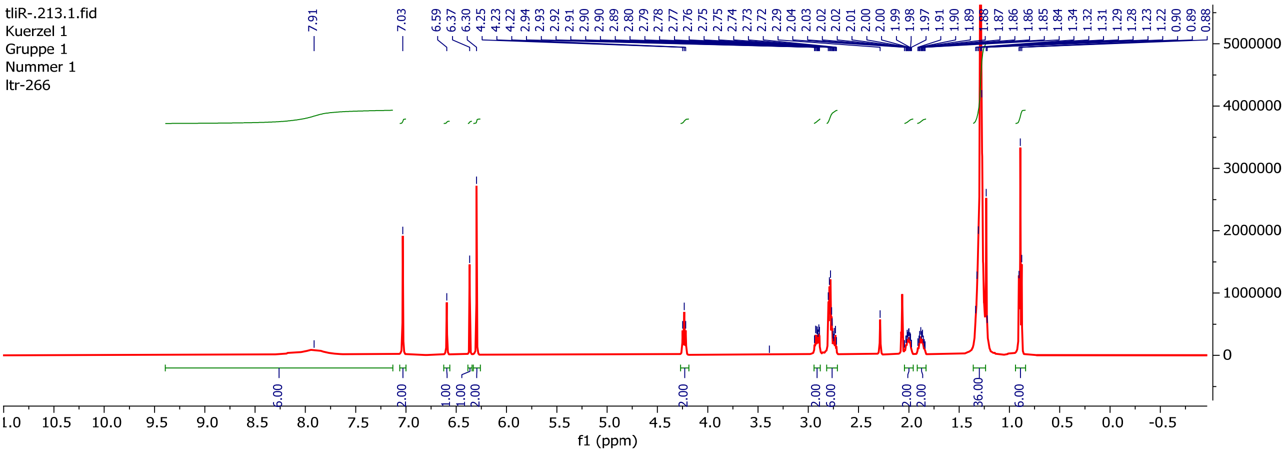

Figure S2: ^{1}H-NMR spectra of ***trans*-2b** in acetone-D6

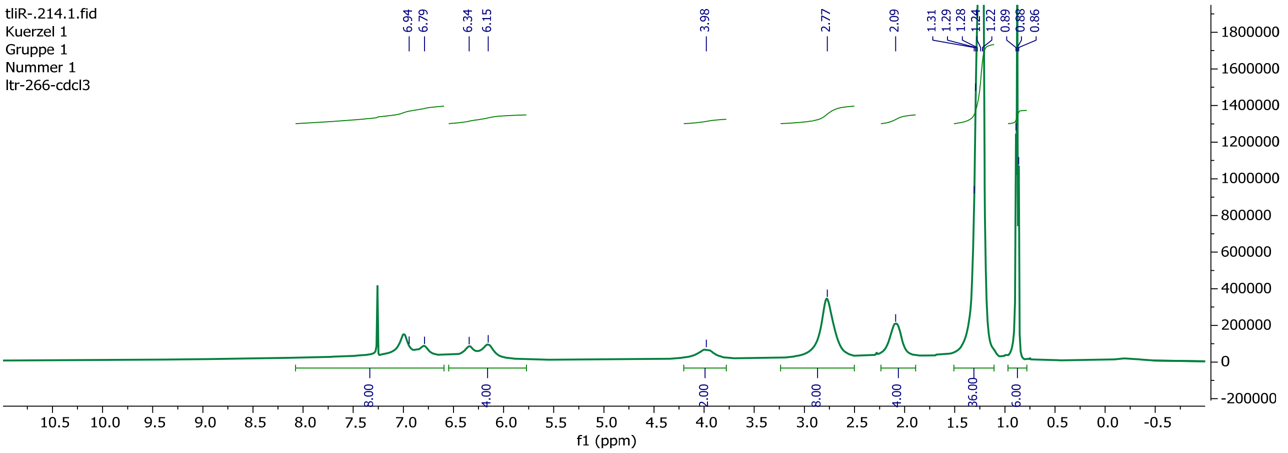

Figure S3: ^{1}H-NMR spectra of ***trans*-2b** in $CDCl_3$

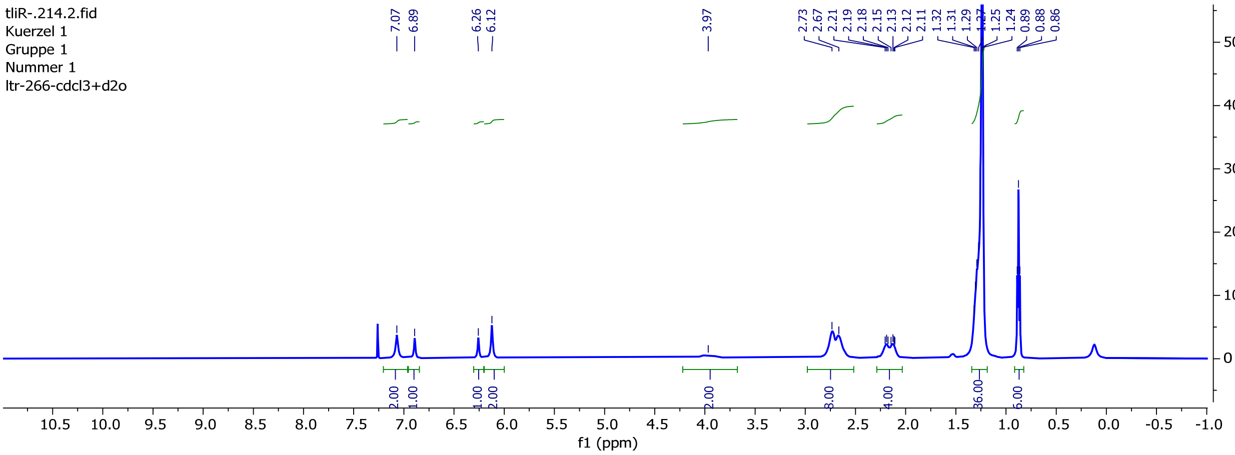

Figure S4: ^{1}H-NMR spectra of ***trans*-2b** in D_2O-saturated $CDCl_3$

4.2 Estimation of the hydrodynamic radius of cage II in $CDCl_3$

The hydrodynamic radius r_h of cage **II** was estimated using a semi-empirical approach towards the modified Stokes-Einstein equation (equation 1).[5] This approach has been used before for size estimation of similar systems.[6] The equation was solved numerically using the Math Input-function of Wolfram Alpha.

$$\mathrm{D} = \frac{k_B T}{\left(\frac{6}{1 + 0.695\left(\frac{rsolv}{rh}\right)^{2.234}}\right) \times \pi\eta r_h} \qquad \text{(Equation 1)}$$

D = Diffusion coefficient obtained from DOSY-measurements [2.9×10^{-10} m^2 s^{-1}]

k_B = Boltzmann constant [$1.3806485 \times 10^{-23}$ $m^2 \cdot kg \cdot s^{-1} \cdot K^{-1}$]

T = Temperature [298 K]

r_{solv} = Hydrodynamic radius of the solvent [0.260×10^{-9} m][6]

r_h = Hydrodynamic radius of the analyte [m]

η = Viscosity of the solvent at 298 K [0.542×10^{-3} $kg \cdot m^{-1} \cdot s^{-1}$]

r_h = 1.4 nm

The diffusion coefficient of cage **II** was measured by DOSY NMR experiments.

Sample preparation: 2.98 mg (4.00 µmol) of *trans*-**2b** was weighed into a GC vial followed with 200 µL water saturated $CDCl_3$. The obtained mixture was sonicated for 1 min to afford a clear solution [$c_{(cage\ II)}$ = 3.33 mM], which was carefully transfered into a 3mm NMR tube. The sample was used for NMR measurement on a 600 MHz NMR instrument at 20 °C.

The signal at 0.88 ppm (refers to the four methyl groups on undecanyl feet) was picked for the intensity decrease curve fitting.

For more instrument/operational details, see section 1.

The diffusion fitting curve from the measurement of D [2.9×10^{-10} m^2 s^{-1}] is shown here:

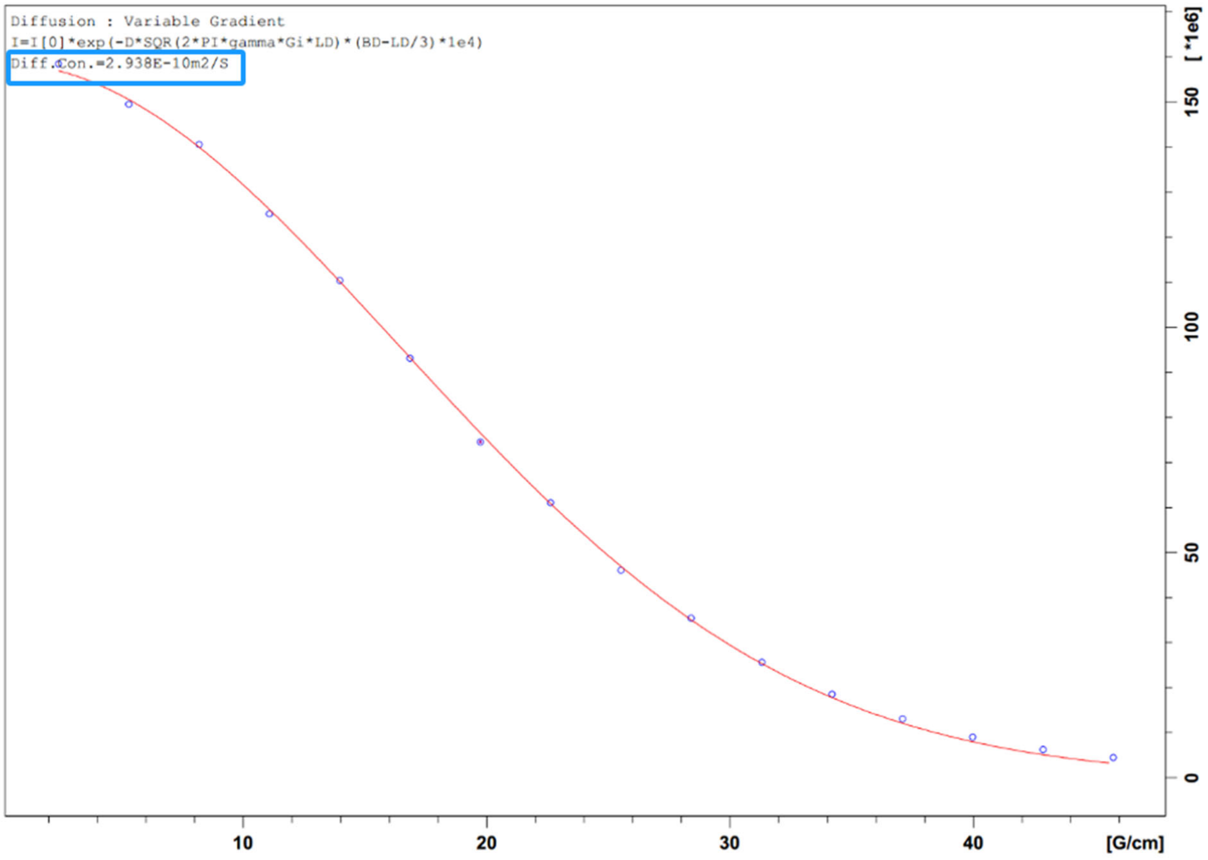

4.3 Dissociation study of cage II with MeOH

The assembly stability of cage **II** was tested by the addition of MeOH as a polar additive.

For each measurement, cage **II** in $CDCl_3$ (3.33 mM) was charged into a 3 mm NMR tube, and a specific amount MeOH was added as indicated in the following table. The DOSY NMR experiment of each sample was measured and the results were summarized. Complete disassembly of the cage was observed for >200 equiv. of MeOH/cage.

Equiv. of MeOH added	D ($X10^{-10}$ m^2s^{-1})
0	3.07
50	3.46
100	3.54
200	4.22

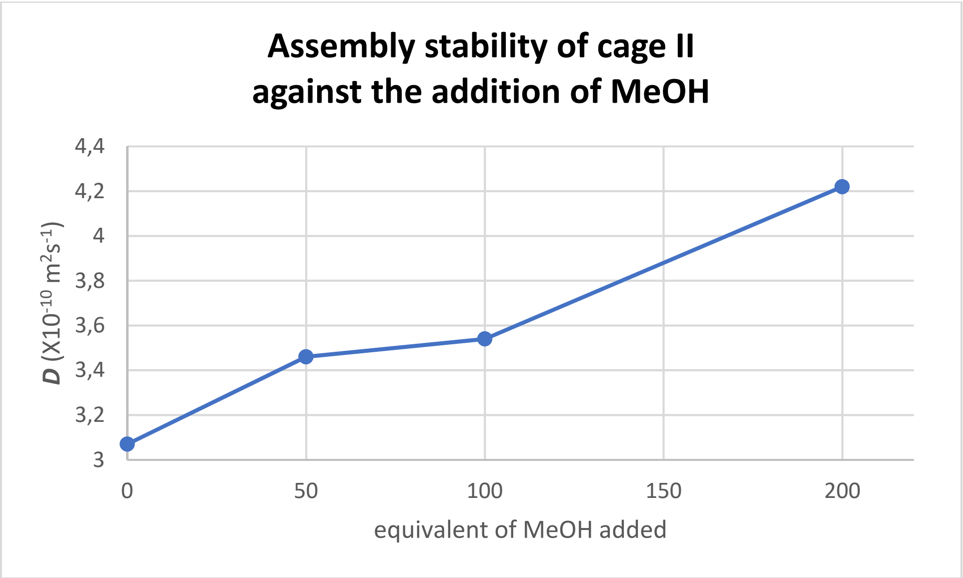

4.4 Binding studies of the window[1]resorcin[3]arenes cage II with tetrabutylammonium salts

Both tetrabutylammonium bromide (TBAB) and tetrabutylammonium tetrafluoroborate ($TBABF_4$) were used in this section. The binding studies of wRS cage **II** with TBAB and $TBABF_4$ were carried out separately.

Sample preparation with TBAB: 2.98 mg (4.00 µmol) of ***trans*-2b** was weighed into a GC vial. The indicated amount of TBAB was added with 200 µL $CDCl_3$ via micro-syringe as stock solution. The obtained mixture was sonicated for 1 min to afford a clear solution, and careful transfered into a 3mm NMR tube. This sample was used for NMR measurement.

Entry	Cage **II**/TBAB	m_{wRS} (mg)	m_{TBAB} (mg)	D_{cage}($x10^{-10} m^2s^{-1}$)	D_{TBAB}($x10^{-10} m^2s^{-1}$)
1	1:0	2.98	0	2.9	-
2	1:0.33	2.98	0.072	2.8	-
3	1:0.5	2.98	0.108	2.8	-
4	1:1	2.98	0.216	2.8	-
5	1:2	2.98	0.432	2.9	-
6	1:3	2.98	0.648	2.7	2.9
7	1:4	2.98	0.864	2.7	2.9
8	1:5	2.98	1.080	2.7	3.3
9	0:1	0	1.080	-	7.7*

*the diffusion coefficient of free TBAB was not shown in the graphics.

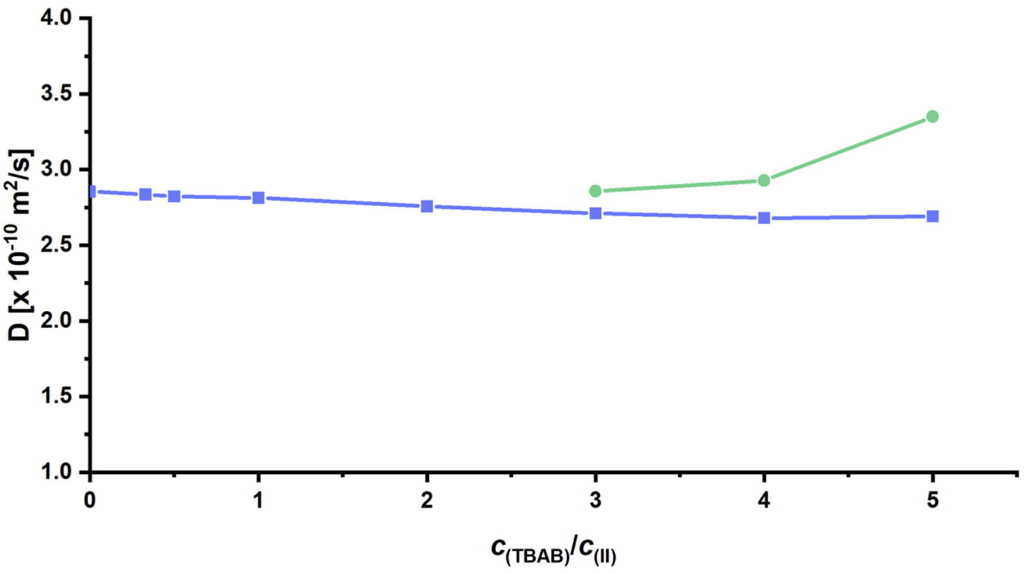

Figure S5: Diffusion coefficients *D* of the cage **II** (■) and unencapsulated TBAB (●).

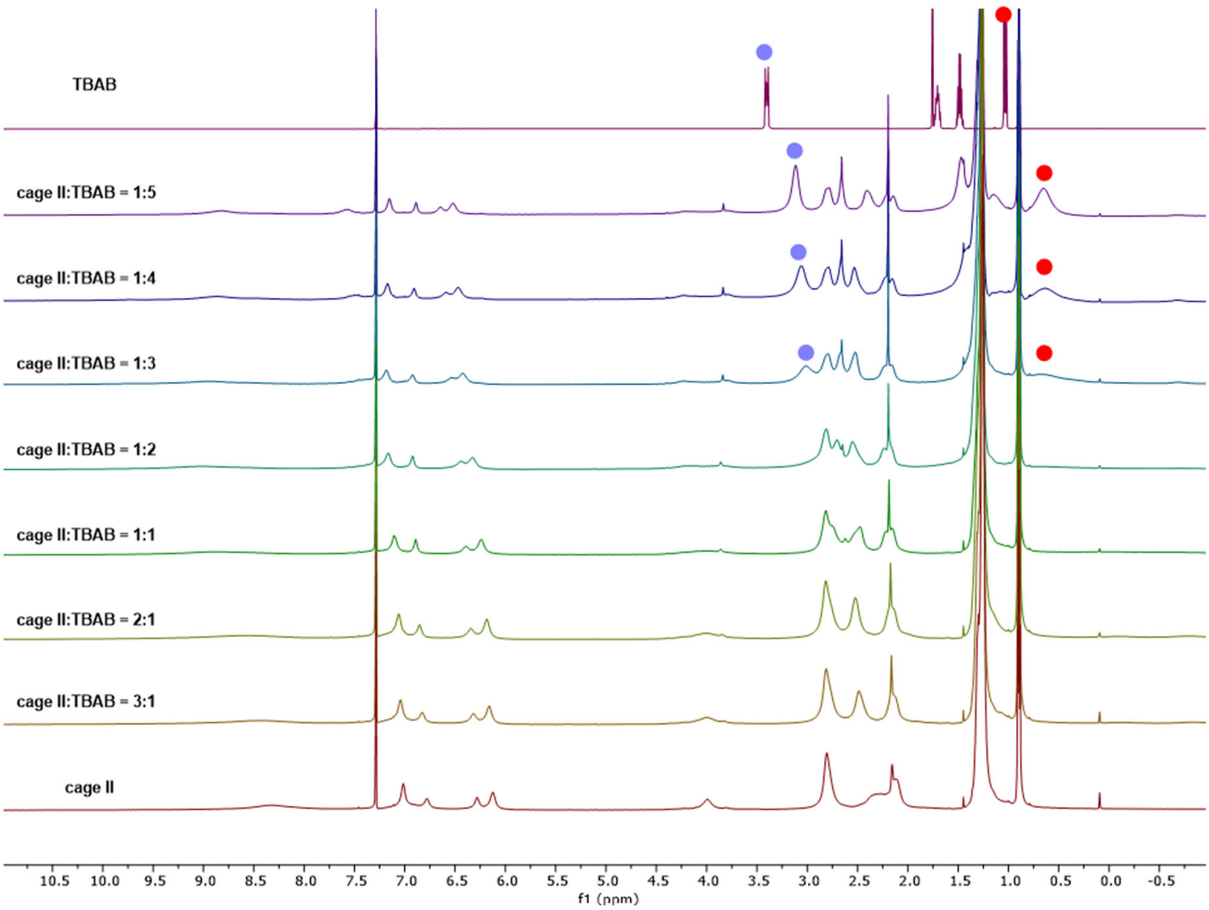

Figure S6: ^{1}H NMR stacking of TBAB titration experiment with cage **II**.

Sample preparation with $TBABF_4$: 2.98 mg (4.00 µmol) of ***trans*-2b** was weighed into a GC vial. The indicated amount of $TBABF_4$ was added as stock solutions together with 200 µL $CDCl_3$ via micro-syringe. The obtained mixture was sonicated for 1 min to afford a clear solution, and then carefully transfered into a 3mm NMR tube. The sample was used for NMR measurement.

Entry	Cage **II**/$TBABF_4$	m_{WRS} (mg)	m_{TBABF4} (mg)	D_{cage}($x10^{-10} m^2s^{-1}$)	D_{TBABF4}($x10^{-10} m^2s^{-1}$)
1	1:0	2.98	0	2.9	-
2	1:0.33	2.98	0.073	2.8	-
3	1:0.5	2.98	0.110	2.9	-
4	1:0.66	2.98	0.146	2.9	
5	1:1	2.98	0.220	2.9	-
6	1:2	2.98	0.439	2.8	3.3
7	1:3	2.98	0.659	2.9	3.8
8	1:4	2.98	0.878	2.8	3.9
9	1:5	2.98	1.098	2.8	4.1
10	0:1	0	1.098	-	7.3*

*the diffusion coefficient of free $TBABF_4$ was not shown in the graphics.

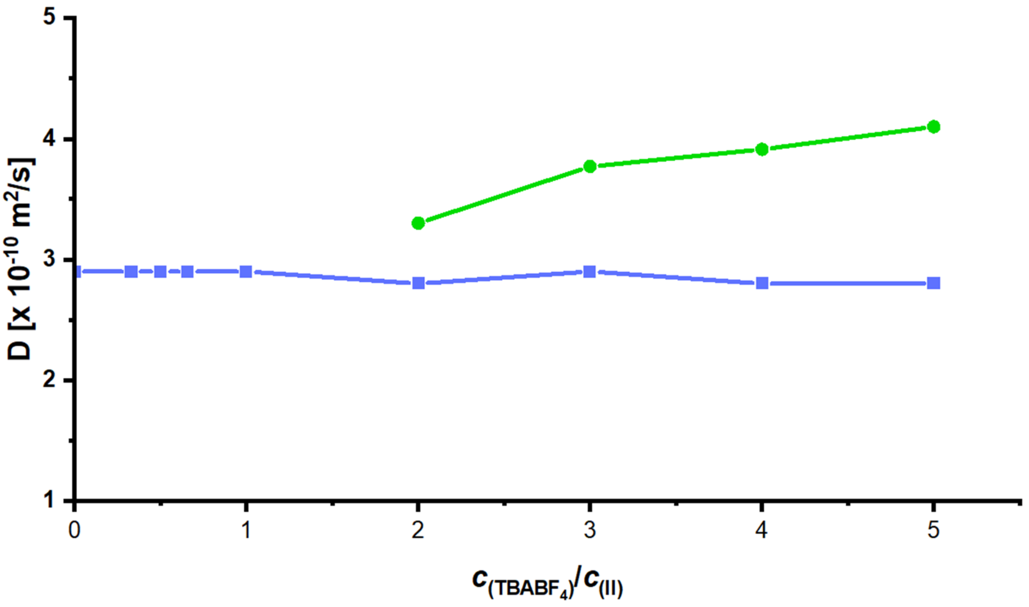

Figure S7: Diffusion coefficients *D* of the cage **II** (■) and unencapsulated $TBABF_4$ (●).

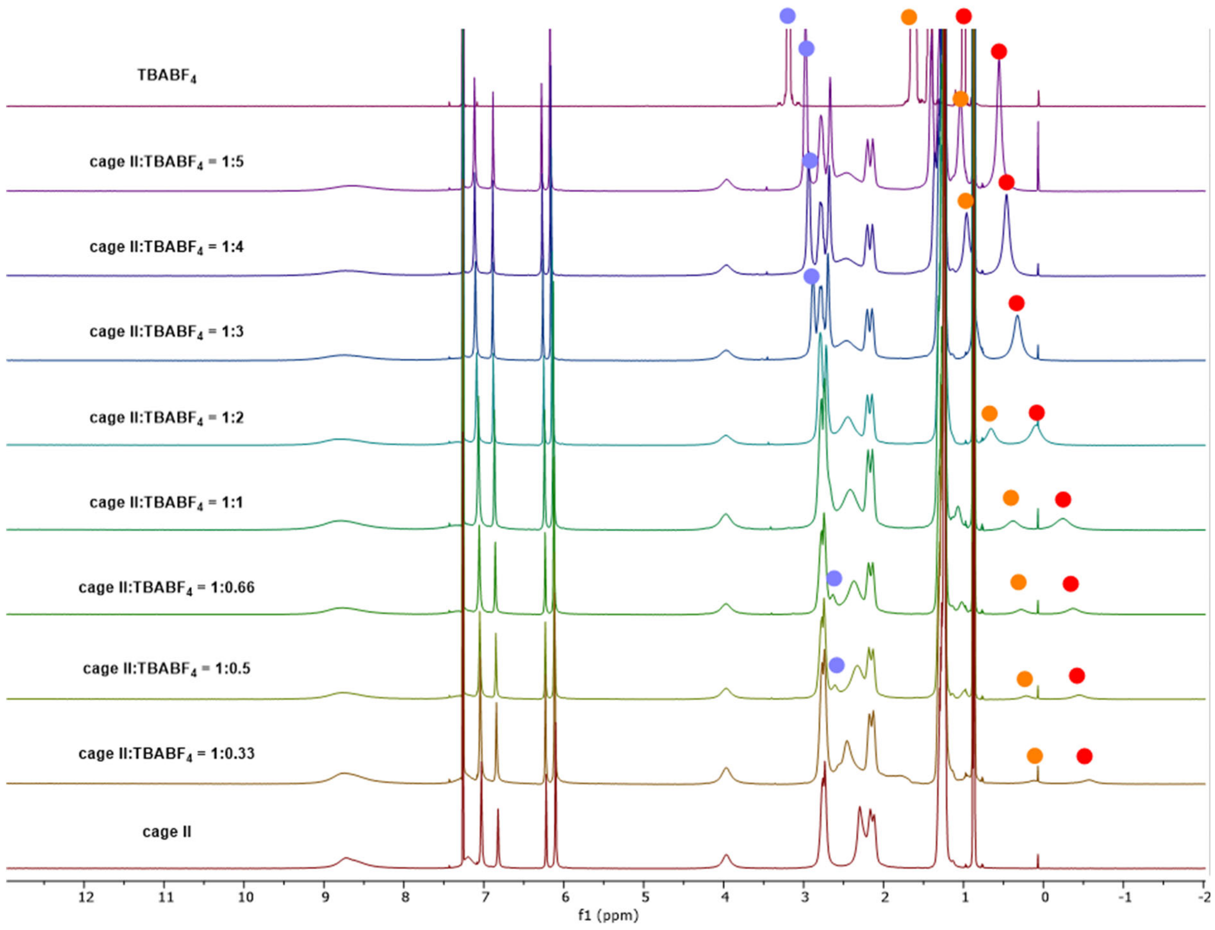

Figure S8: ^{1}H NMR stacking of $TBABF_4$ titration experiment with cage **II**.

4.5 Binding studies of resorcin[4]arene capsule I with tetrabutylammonium bromide (TBAB)

Sample preparation: 4.40 mg (4.00 µmol) of *C11*-resorcin[4]arene was weighed into a GC vial. The indicated amount of TBAB was added as stock solutions together with 200 µL $CDCl_3$ via micro-syringe. The obtained mixture was sonicated for 1 min to afford a clear solution and then carefully transfered into a 3mm NMR tube. The sample was used for NMR measurement.

Entry	Capsule I /TBAB	$M_{capsule\ I}$ (mg)	M_{TBAB} (mg)	$D_{capsule\ I}$ ($x10^{-10}m^2s^{-1}$)	$D_{TBAB-encapsulated}$ ($x10^{-10}m^2s^{-1}$)	$D_{TBAB-unencapsulated}$ ($x10^{-10}m^2s^{-1}$)
1	1:0	4.40	0	2.3	-	-
2	1:0.33	4.40	0.072	2.3	2.4	-
3	1:0.66	4.40	0.143	2.3	2.3	-
4	1:1	4.40	0.215	2.3	2.3	-
5	1:2	4.40	0.430	2.3	2.3	3.9
6	1:3	4.40	0.645	2.3	2.3	5.3
7	1:4	4.40	0.860	2.3	2.5	5.9
8	1:5	4.40	1.075	2.3	2.8	6.1
9	0:1	0	1.075	-	-	7.7*

*the diffusion coefficient of free TBAB was not shown in the graphics.

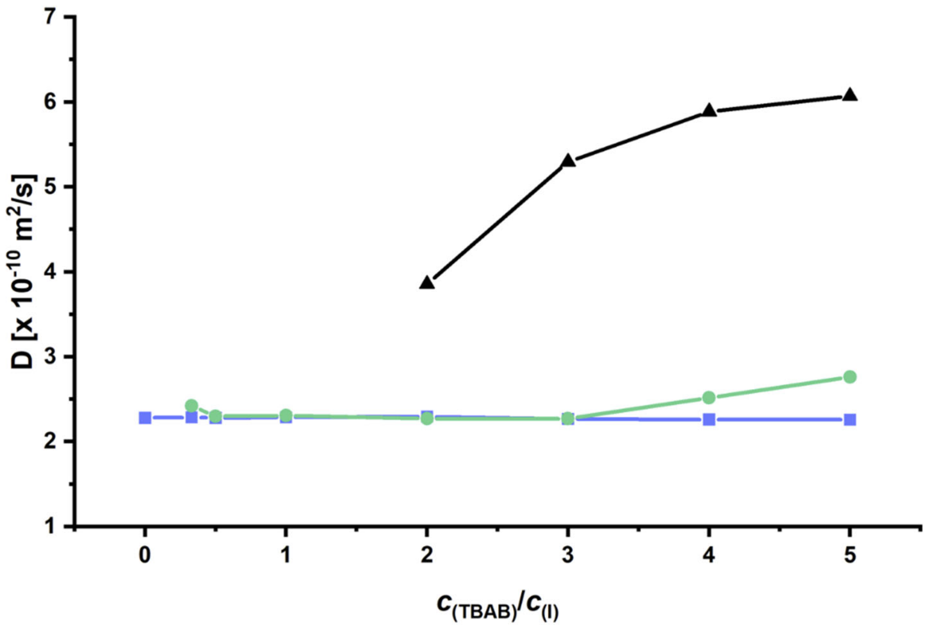

Figure S9: Diffusion coefficients *D* of the capsule **I** (■) and encapsulated TBAB (●), and unencapsulated TBAB (▲).

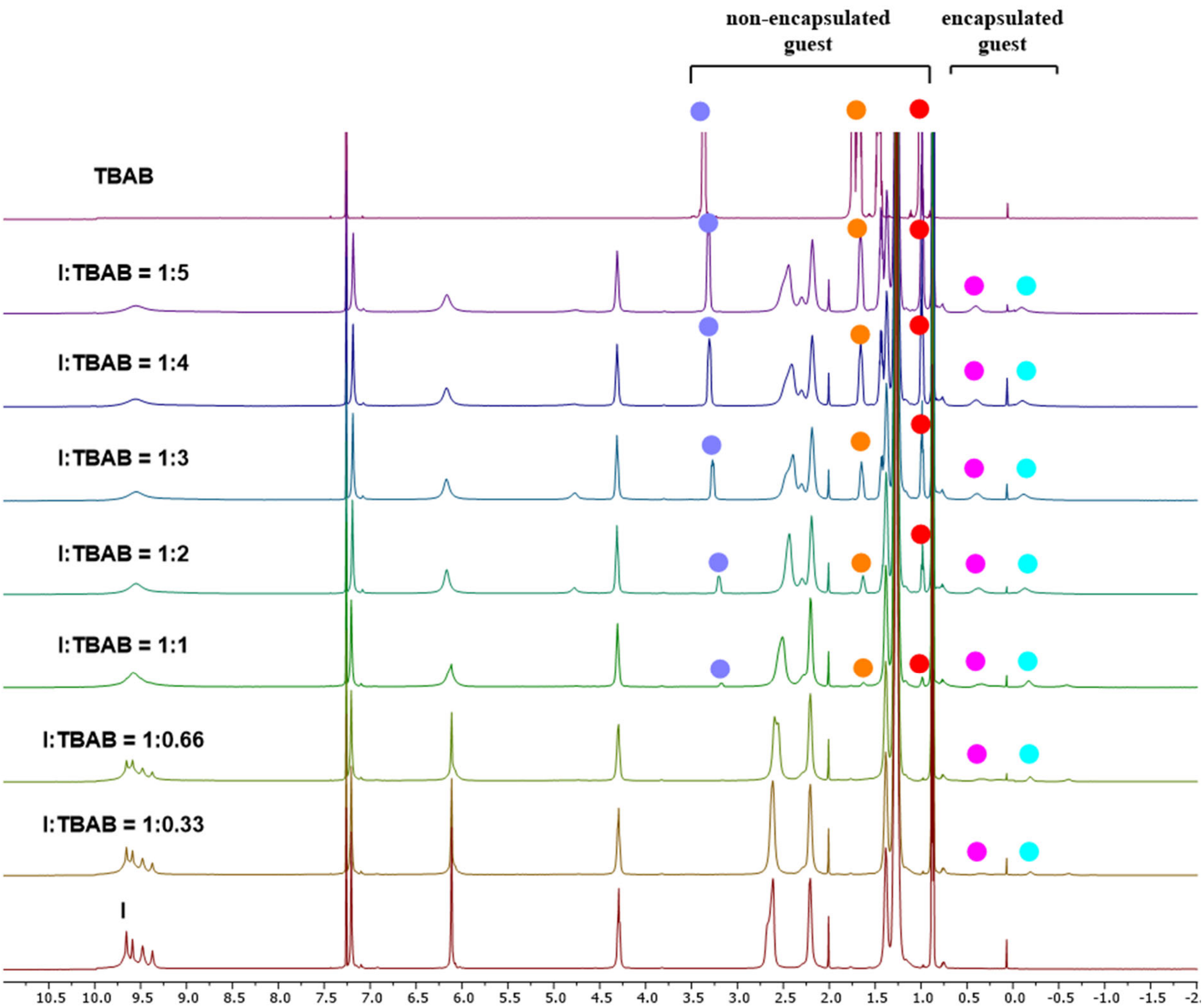

Figure S10: ^{1}H NMR stacking of TBAB titration experiment with capsule **I**.

4.6 The comparison of capsule I & cage II with tetrabutylammonium salts as the guest molecules

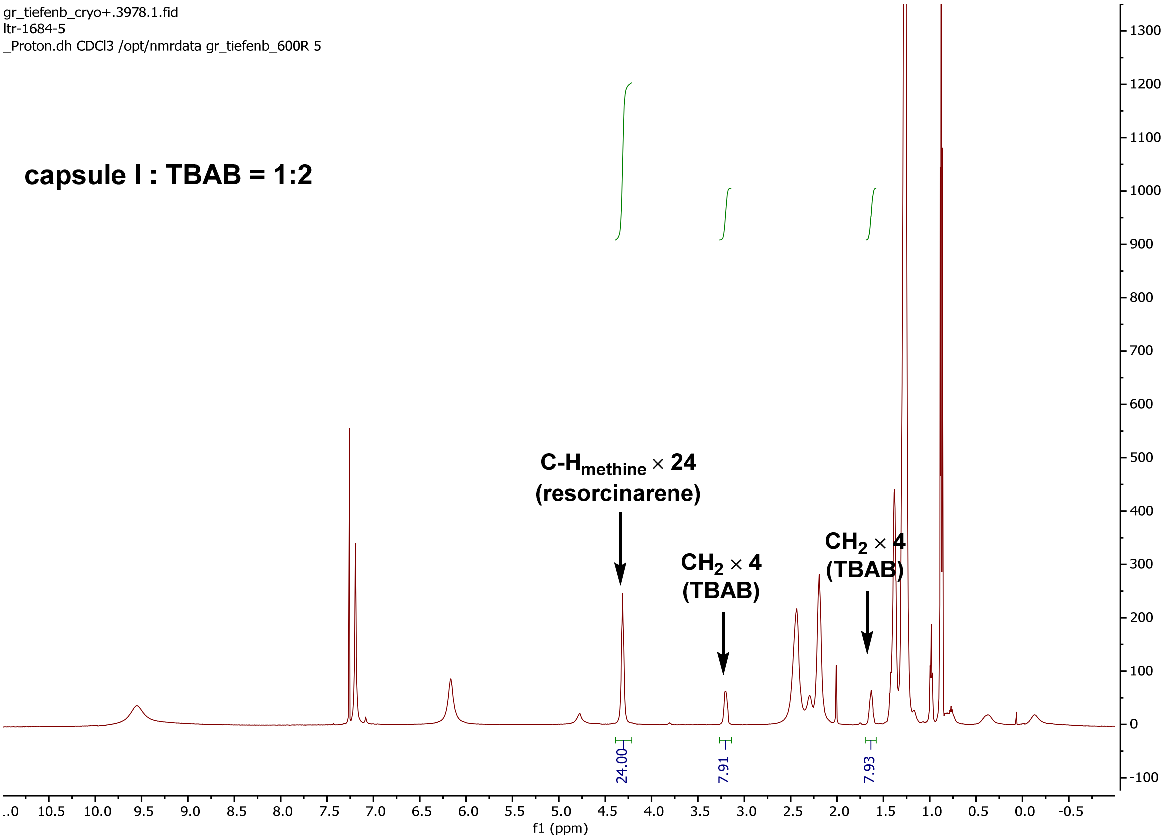

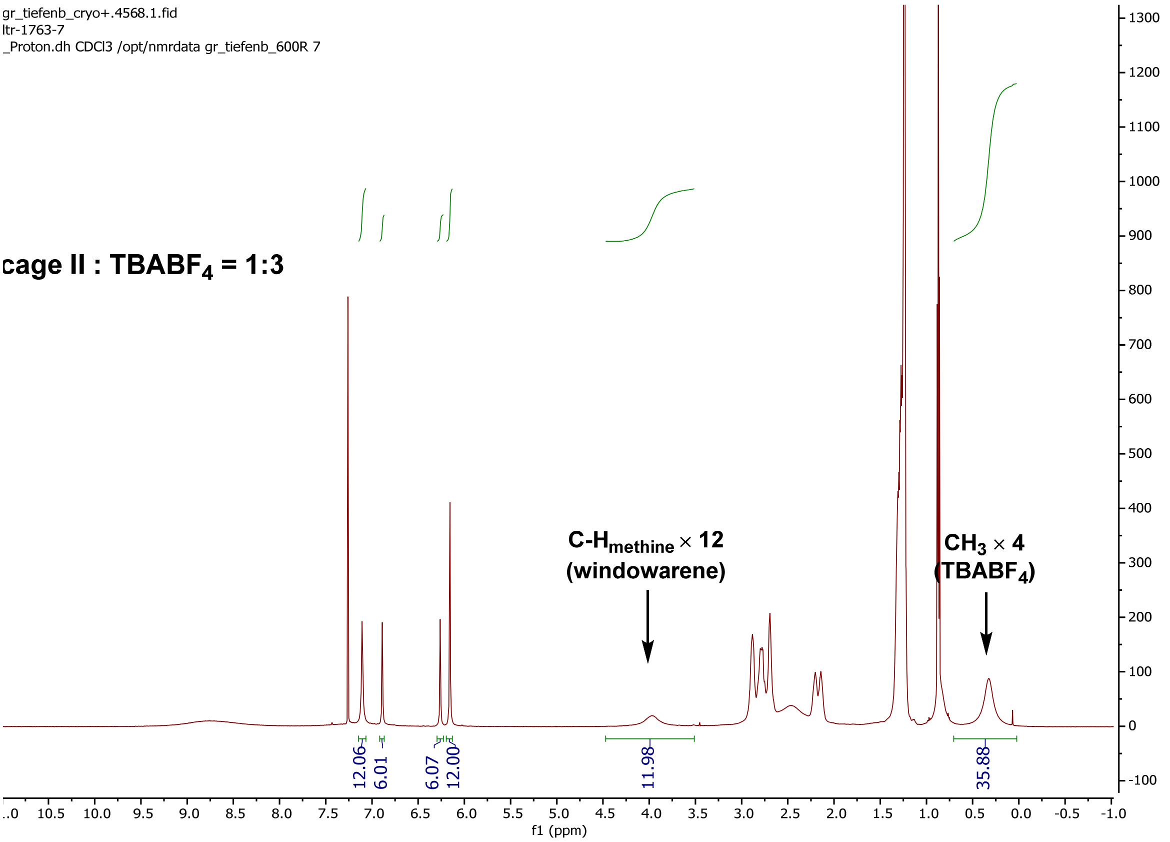

Figure S11: The comparison of **I** and **II** with TBA salts as guest molecules

The integration of unencapsulated TBAB in the ^{1}H NMR spectrum of a mixture of capsule **I**:TBAB = 1:2 indicated that 1 equivalent of TBAB was encapsulated by capsule **I** (Top).

Due to signal overlap in the ^{1}H NMR spectra of the mixture of cage **II** and TBAB (see page S35), $TBABF_4$ as the guest molecule was used here instead. The integration of $TBABF_4$ in the ^{1}H NMR spectrum of a mixture of cage **II**:$TBABF_4$ = 1:3 indicated that the guest molecule was encapsulated in fast exchange on the NMR chemical shift time scale (Bottom).

4.7 Binding studies of the window[1]resorcin[3]arene cage II with tetrahexadecylammonium bromide (THDAB)

Sample preparation: 2.98 mg (4.00 µmol) of ***trans*-2b** was weighed into a GC vial. The indicated amount of THDAB was added as stock solution wogether with 200 µL $CDCl_3$ via micro-syringe. The obtained mixture was sonicated for 1 min to afford a clear solution and carefully transfered into a 3mm NMR tube. The sample was used for NMR measurement.

Entry	Cage **II**/ THDAB	m_{host} (mg)	M_{guest} (mg)	D_{host} ($x10^{-10}m^2s^{-1}$)	D_{guest} ($x10^{-10}m^2s^{-1}$)
1	1:0	2.98	0	3.0	-
2	1:0.33	2.98	0.221	2.9	2.8
3	1:0.66	2.98	0.443	2.9	2.8
4	1:1	2.98	0.664	2.7	2.7
5	1:2	2.98	1.328	2.7	2.7
6	1:3	2.98	1.992	2.6	2.7
7	1:5	2.98	3.320	2.5	3.0
8	0:1	0	3.320	-	4.5*

*the diffusion coefficient of free THDAB was not shown in the graphics.

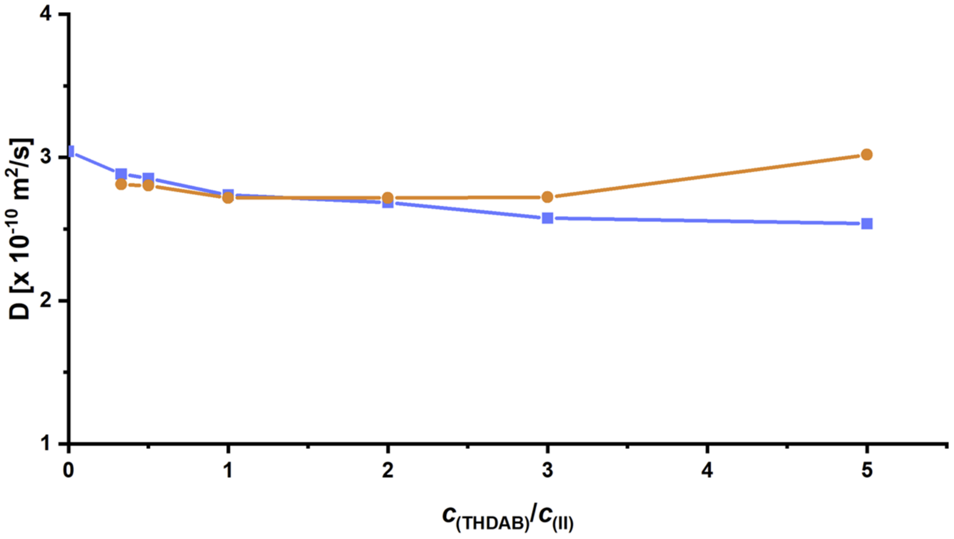

Figure S12: Diffusion coefficients *D* of the cage **II** (■) and THDAB (●).

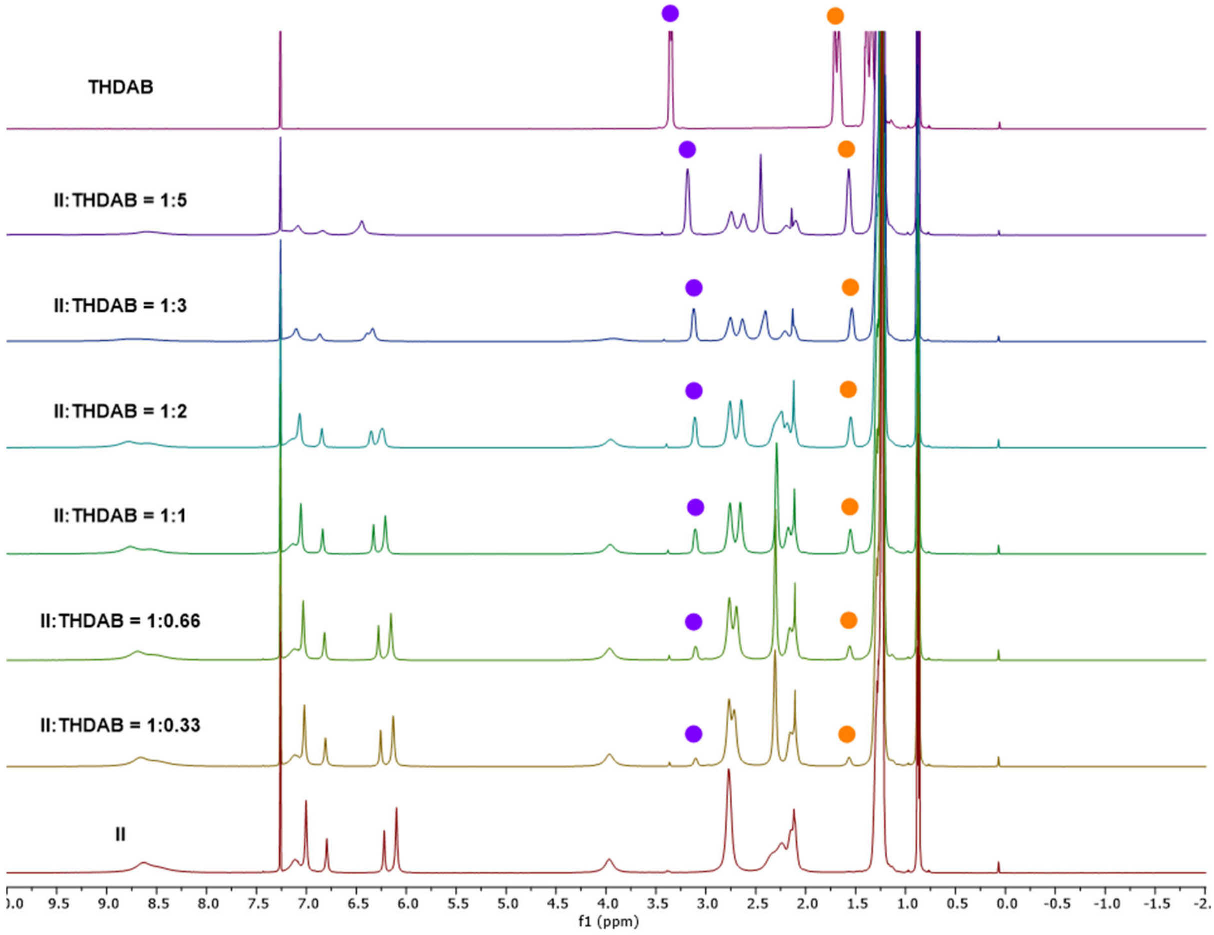

Figure S13: ^{1}H NMR spectra stacking of THDAB titration experiment.

4.8 Guest encapsulation study of tetrahexadecylammonium bromide (THDAB) with both window[1]resorcin[3]arene cage II and resorcin[4]arene capsule I

Encapsulation studies of both capsules **I** & cage **II** in the presence of 1.0 equivalent tetrahexadecylammonium bromide (THDAB) as the guest molecule were conducted.

Procedure: 2.98 mg (4.00 µmol, equal to 667 nmol of cage **II**) of ***trans*-2b** was weighed into a GC vial, and 664 µg (667 nmol) of THDAB was added as stock solution together with 200 µL $CDCl_3$ via micro-syringe. The obtained mixture was sonicated for 1 min to afford a clear solution, and carefully transfered into a 3mm NMR tube. Then, the liquid level of this NMR sample was soaked in a cooling bath of acetone/liquid nitrogen, and the top part of the NMR tube was sealed by carefully melting the glass with a butane torch while uniformly spinning the tube. After being slowly warmed to room temperature, the sample is ready to be used for the NMR measurement.

The NMR sample of resorcin[4]arene capsule-THDAB 1:1 solution was prepared following the same procedure, with 4.40 mg (4.00 µmol, equal to 667 nmol of hexameric capsule) of *C11*-resorcinarene.

Note: completely sealing the NMR tubes with a butane torch proved to be important, which prevents the volatilizing of $CDCl_3$ and the changing of the water contents.

The well-prepared NMR samples were loaded on a Bruker automated 600 MHz NMR instrument, and the DOSY experiments were performed every 24 hours for 7 days. The results were summarized in the following table and graphics.

**Time (day)**	0	1	2	3	4	5	6	7
**D(cage II)**	2.7	2.7	2.7	2.7	2.6	2.6	2.7	2.6
**D(THDAB)**	2.7	2.7	2.7	2.7	2.7	2.7	2.7	2.7

**Time (day)**	0	1	2	3	4	5	6	7
**D(capsule I)**	2.2	2.2	2.2	2.3	2.2	2.2	2.2	2.2
**D(THDAB)**	3.0	2.9	2.9	2.8	2.9	2.9	2.9	2.8

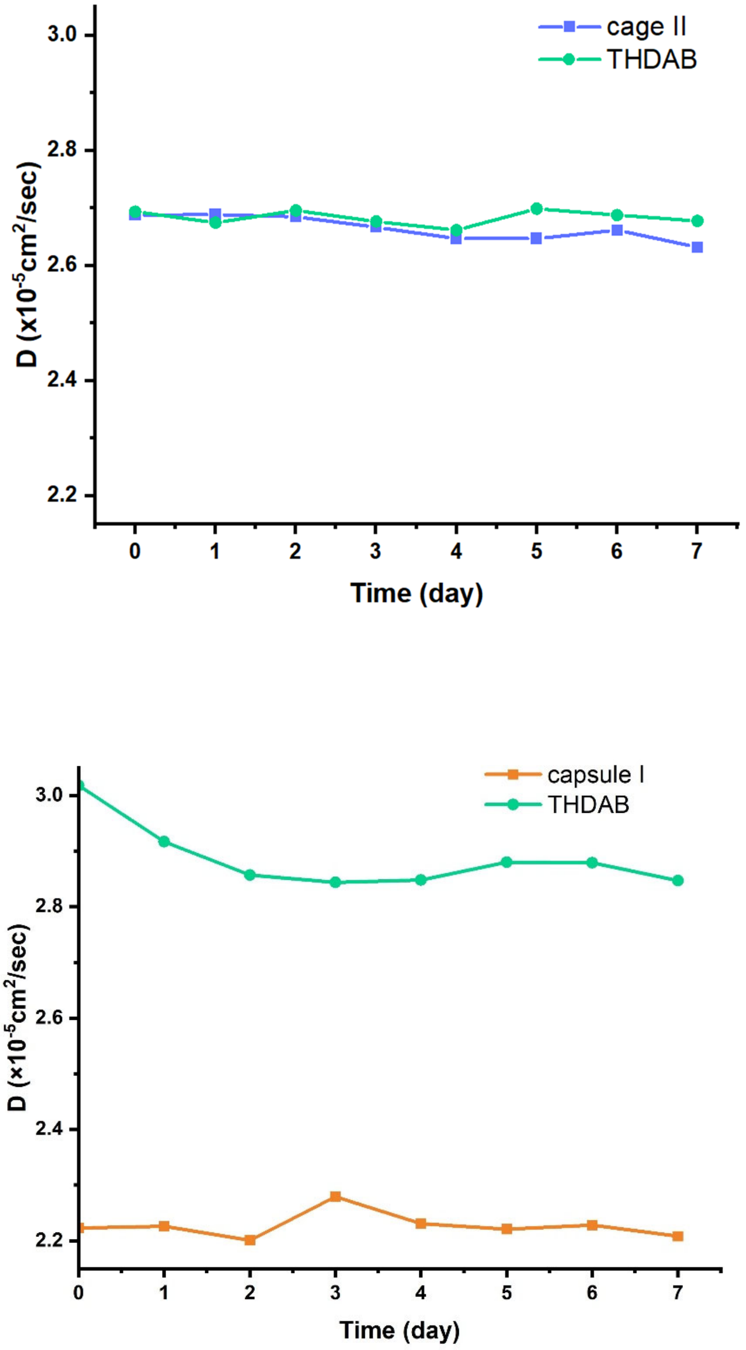

Figure S14. Diffusion coefficients of the 1:1 mixture of cage **II** & THDAB over 7 days (top); diffusion coefficients of the 1:1 mixture of capsule **I** & THDAB over 7 days (bottom)

4.9 Assembly study of the mixture of capsule I and cage II

We performed ^{1}H NMR and DOSY experiments with both capsule I [3.33 mM] and cage **II** [3.33 mM] mixed in one solution. The mixture of **I&II** shows complete self-sorting behavior. No hybrid assembly was observed. Only two assembly species were observed in the DOSY experiment and their diffusion coefficients match capsule **I** and cage **II**.

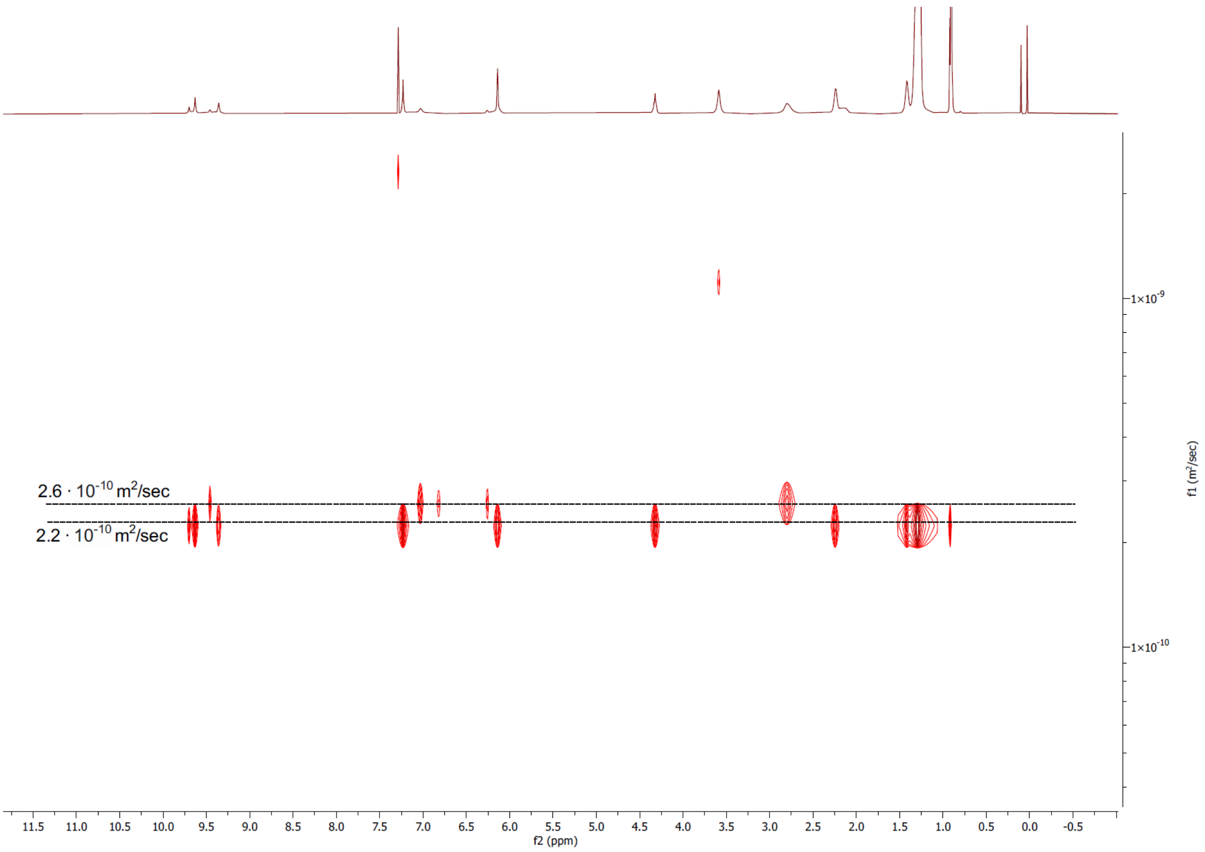

5. Computational Study

Molecular dynamics simulation and free energy calculation

To shed light on the dynamical behaviour of the self-assembly process, we began with the fundamental building block of the self-assembled structure. First, we employed enhanced sampling metadynamics[7][8] simulation to investigate more on the structural properties of the trans isomer which is experimentally the most soluble building block. Using metadynamics, we can interconvert between potential structural conformations (in this case, crown and chair conformation) and obtain the related free energy profile.

After that, to gain further insight into the dynamics of cage **II** and its encapsulation behaviour, we performed classical equilibrium molecular dynamics simulations for cage **II** without any guest molecule and then with guest molecules TBAB and TDDAB.

Metadynamics simulations to determine the most stable conformer

We performed a metadynamics simulation of the trans isomer placed in a simulation box with size (46.591×46.972×43.671 Å^3) solvated with chloroform with periodic boundary condition. The simulation has been performed at 300K sampling a 50 ns trajectory with a 2 fs timestep using GROMACS[9] patched with PLUMED2[10,11] code for the application of the metadynamics bias. To drive the interconversion between crown and chair conformer the bias has been added along a collective variable (CV) defined as antisymmetric linear combination of cosine of dihedral angle ϕ_1 (connecting carbon atom 1,2,3 and 4) and cosine of dihedral angle ϕ_2 (connecting Carbon atom 7,6,5,4) as shown in Fig. S15.

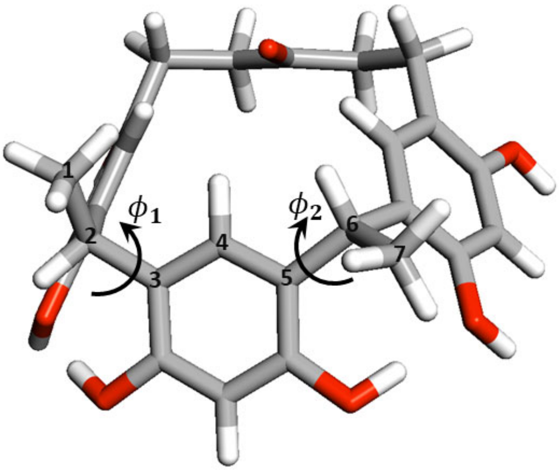

Figure S15: showing the description of the dihedral angle considered for the construction of CV

$$C12 = -cos\ (\phi_1)\ + cos\ (\phi_2)$$

From the metadynamics simulation it is found that the interconversion from crown to chair

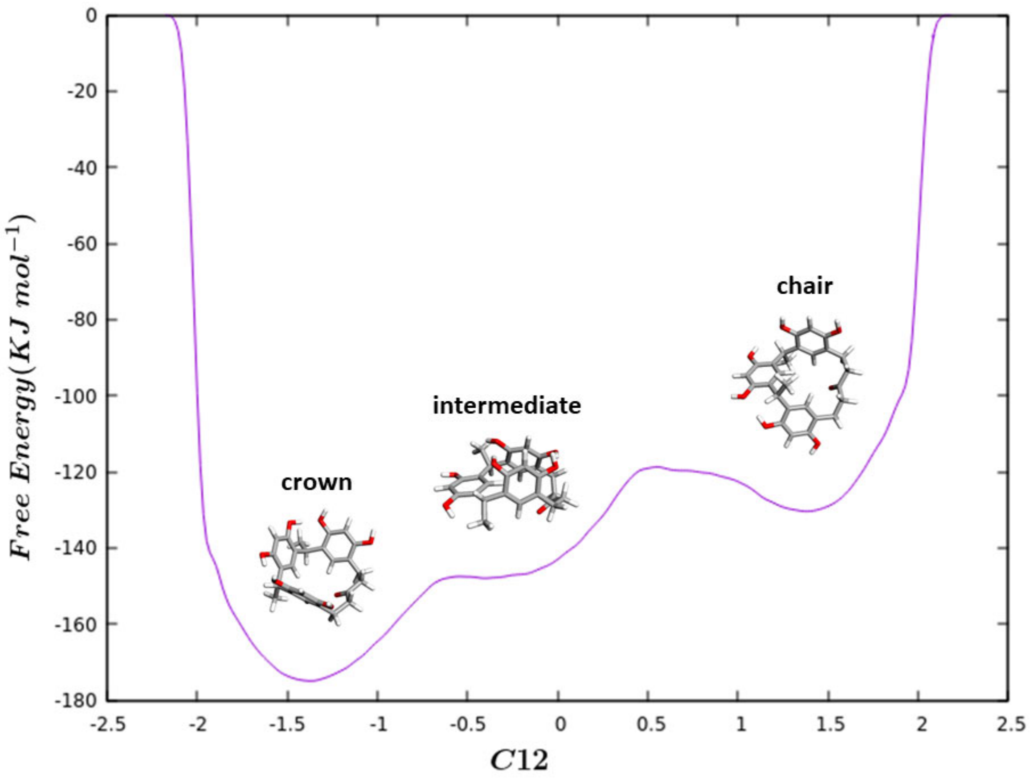

Figure S16: Free energy profile for the transition from crown to chair conformation showing that during the interconversion the system passes through an intermediate conformation.

form happens through an intermediate conformation which lies in between the crown and chair conformation in the free energy diagram as shown in fig S16. Nonetheless, the stability of the crown conformation ($\Delta G_{crown \leftarrow chair} \simeq -40$ kJ/mol) is such that we can clearly state that assembly can occur only in this conformation.

Classical molecular dynamics simulation:

Simulation details:

To investigate the dynamical behaviour of cage **II** we performed molecular dynamics simulation of cage **II** without any guest molecule and then cage **II** with TBAB and TDDAB, respectively. The host and guest molecules have been parameterized using an all-atoms GAFF[12]/RESP[13] procedure as implemented in the antechamber program. RESP charges were calculated using GAUSSIAN 09 [13] at the HF/6-31G* level. Then one host container and one guest molecule are put inside a simulation box and the whole system is solvated with chloroform. The simulation box size of each case is enclosed in the following table.

System	Simulation box size($Å^3$)
Empty cage **II**	$(57.591 \times 57.761 \times 57.512)$
Cage **II** + TBAB	$(84.815 \times 86.284 \times 89.932)$
Cage **II** + TDDAB	$(84.815 \times 86.284 \times 89.932)$

To get the initial structure of empty Cage II, we started from the guess structure as preliminary structure. This preliminary structure was introduced into the simulation box of the specified dimensions and subjected to an initial minimization procedure aimed at attaining a configuration with the minimum possible energy. Then the system is heated up to 300 K in the NVT ensemble for 150 ps with 0.5 fs step using the velocity rescaling algorithm[14]. The pressure has been subsequently adjusted using NPT simulation for 10 ns with Parrinello-Rahman barostat[15] using 1fs step size. Then for each of the systems a production run of 100 ns was performed with 1fs as step size. Then, the equilibrated system was set for 100 ns production run with 1fs as step size.

Following this, we proceeded with the investigation of guest encapsulation using the equilibrated empty Cage II. Our focus centered on studying the encapsulation of both TBAB and TDDAB. The smaller size of TBAB facilitated its accommodation within the Cage II cavity. Conversely, dealing with the larger guest molecule TDDAB within Cage II required the application of replica exchange molecular dynamics (REMD) to enhance conformational

sampling. This was crucial for acquiring a suitable initial structure for the host-guest assembly. To optimize the conformational space, we employed REMD with 7 replicas spanning temperatures from 250 K to 500 K, attempting exchanges every 100 fs in the NVT ensemble for a duration of 1000 ps.

Subsequently, each of the two systems underwent an initial minimization process to achieve a structure with minimal energy. The system was then heated to 300 K in the NVT ensemble over 150 ps with a 0.5 fs step, followed by pressure equilibration in the NPT ensemble for 10 ns using the Parrinello-Rahman barostat with a step size of 1 fs. A production run of 100 ns, with a step size of 1 fs, was conducted for each system. To enhance statistical reliability, five separate production runs were carried out for each system.

Mean Square Displacement and corresponding diffusion coefficient:

Mean Square Displacement (MSD) is calculated using the Einstein relation which is given as

$$MSD(\tau) = \langle \frac{1}{N} \sum_{i=1}^{N} |r - r(\tau)|^2 \rangle$$

Where N is the number of equivalent particles the MSD is calculated over, r is their coordinates and τ is the lag-times. The diffusion coefficients are calculated from the MSD via the relation

$$D = \frac{MSD}{6\tau}$$

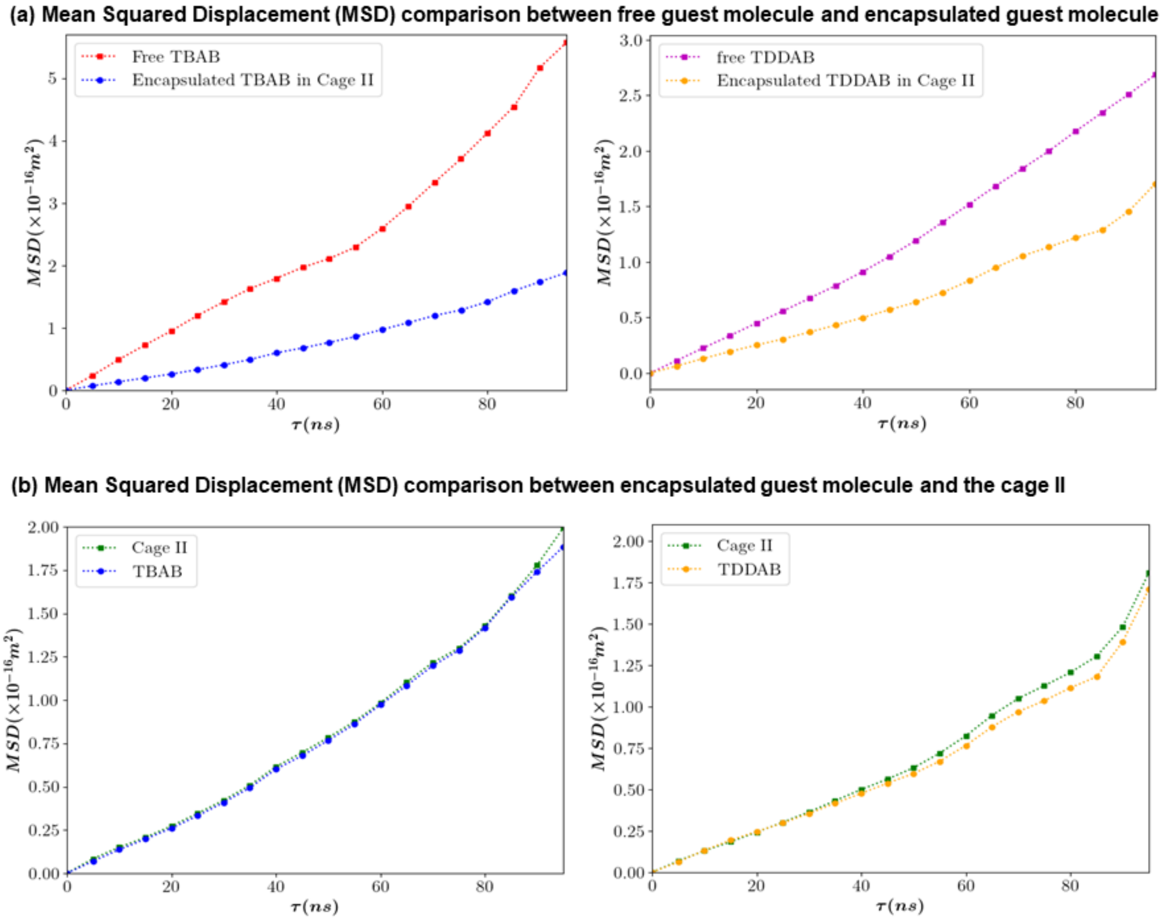

Figure S17. Mean-Squared displacement comparison a) between free guest molecule (TBAB And TDDAB) and the corresponding encapsulated form in cage **II**, b) between the encapsulated guest molecule and the cage **II** in each case.

Root mean squared deviation (RMSD):

The root mean square deviation (RMSD) is calculated with respect to a fully ordered hexameric reference structure optimised at 0 K to check dynamical stability of cage **II**. RMSD at a particular instance is a measure of average deviation of atomic positions compared to a reference structure according to the equation below:

$$RMSD(x, x^{ref}) = \sqrt{\frac{1}{n}\sum_{i=1}^{n}|x - x^{ref}|^2}$$

where x refers to instantaneous position and x^{ref} is the position of the reference structure. The averaging is done over all the atoms present in the structure. Here n is the number of atoms present in the structure.

In the case of encapsulation of TBAB the RMSD of cage **II** is slightly lower ($\sim 0.83 \times 10^{-9}\ m$) than that of encapsulation of TDDAB ($\sim 0.88 \times 10^{-9}\ m$), which is because of the larger size of the TDDAB compared to TBAB as described below in the Figure S18.

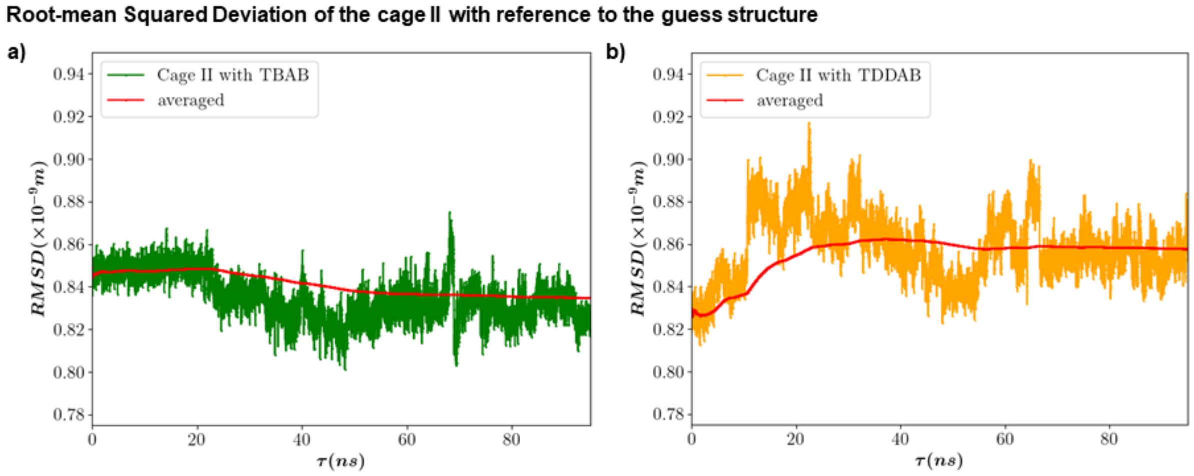

Figure S18. Root-mean Squared deviation (RMSD) of the cage **II** a) with TBAB and b) with TDDAB

Intermolecular H- bond:

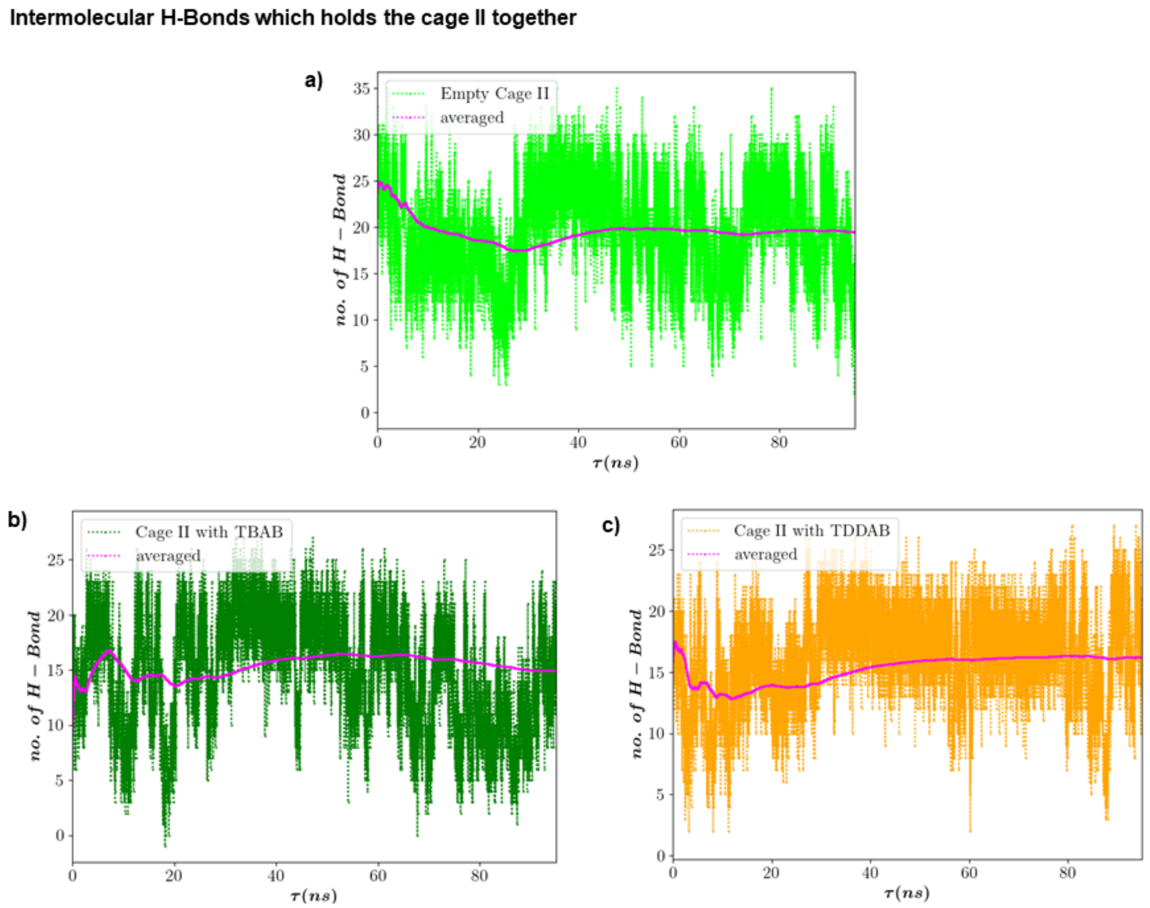

Figure S19. Intermolecular H-Bonds that holds the cage **II** together a) for cage **II** without any guest molecule, b) for capsulation of TBAB as guest molecule and c) for capsulation of TDDAB as guest molecule

The amount of intermolecular H-bonds that hold cage **II** together has been estimated using VMD[16]. On average, Cage **II** without any guest molecule is maintained throughout the simulation by 20 intermolecular H-bonds, as illustrated in the figure below. While with TBAB and TDDAB inside the Cage **II** as guest molecule, around 15 intermolecular H-bonds hold the cage together.

Number of water molecules within H-bonded network of Cage II:

From the above plot we can see that the Cage **II** most likely incorporates 8 water molecules which stays in the H-bonded network of Cage **II**.

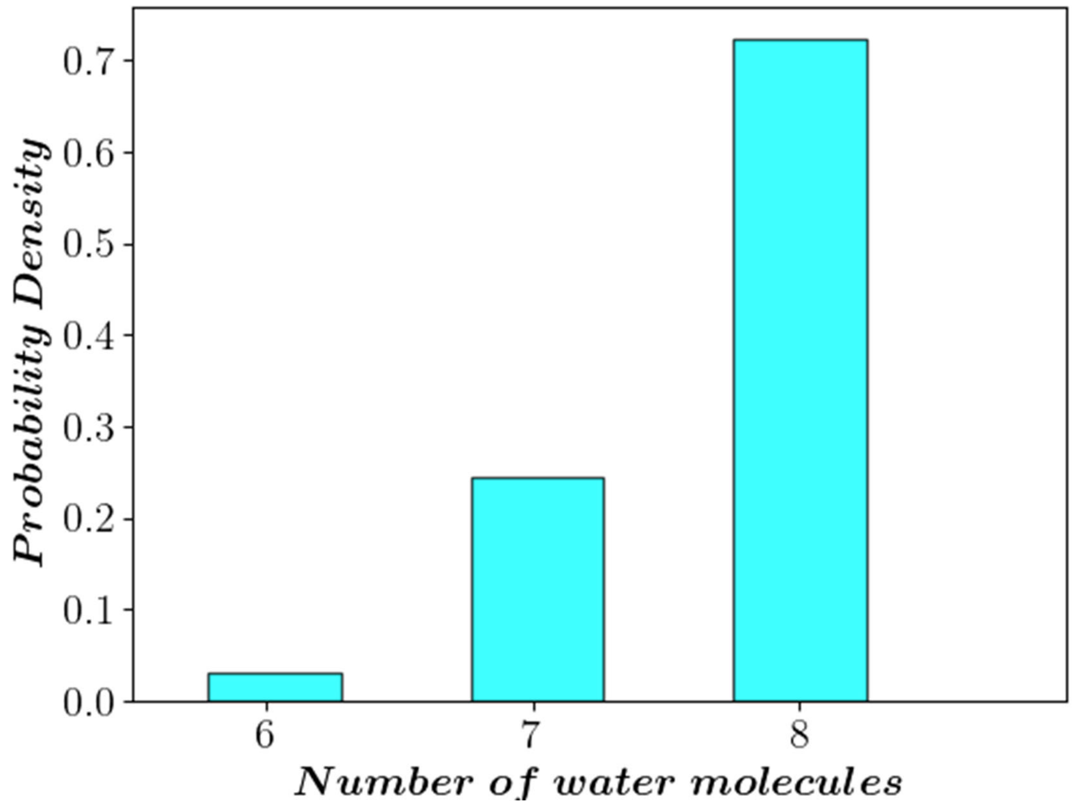

Figure S20. Probability density of water molecules participating in the H- bonded network of Cage **II**

Snapshot of Cage II with TDDAB :

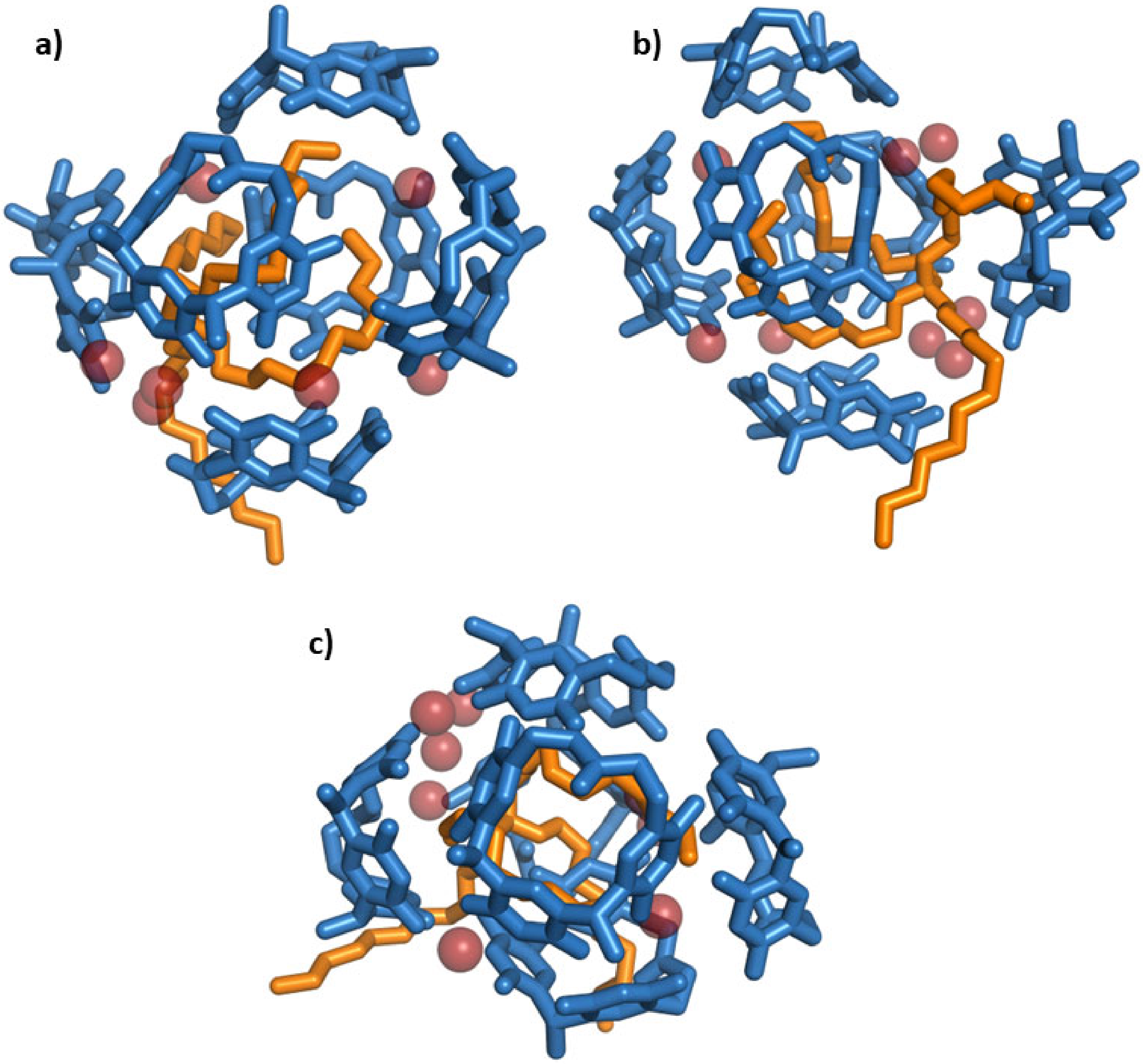

Figure S21. Snapshot of Cage **II** with TDDAB from different orientations

Snapshot of reference Cage II optimized at 0K:

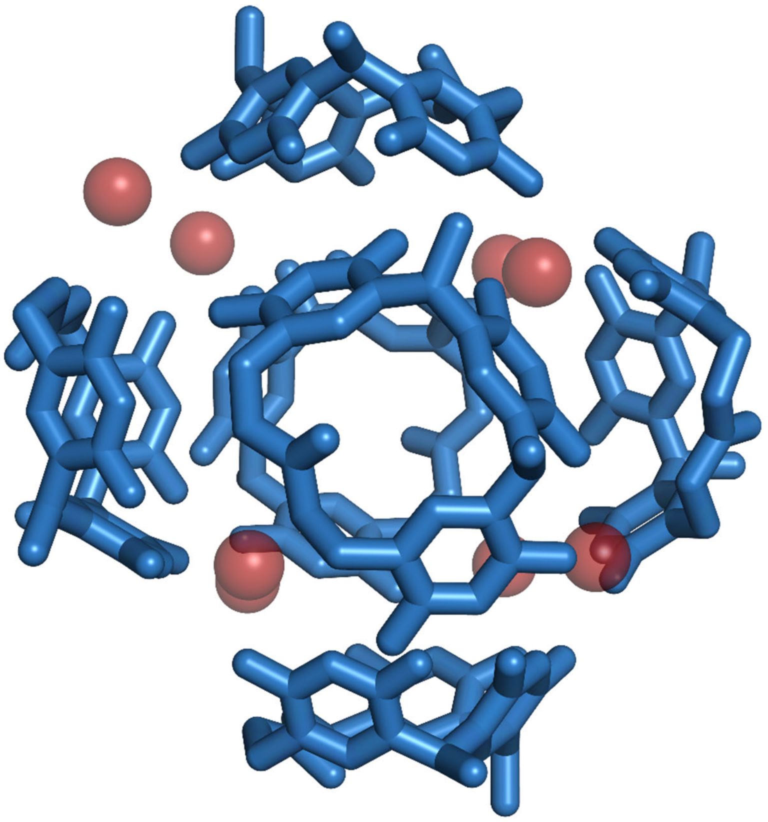

Figure S22. Snapshot of highly ordered hexameric structure of Cage II optimized at 0 K used as a reference for RMSD calculation.

6. Model of Cage II

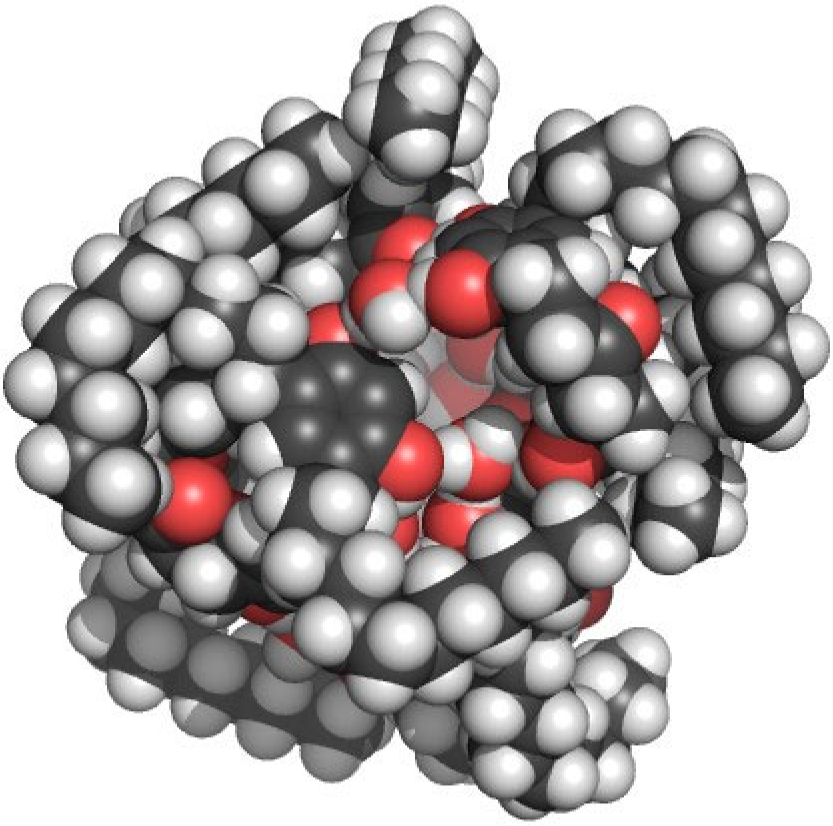

Figure S23. Molecular model of cage **II**.

7. Application of Window[1]resorcin[3]arene Cage II in Friedel–Crafts Reaction

7.1 Synthesis of 4-octadecyl benzyl chloride

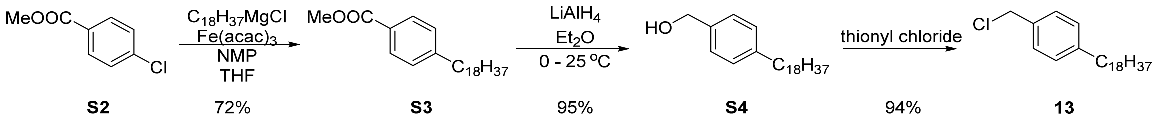

Scheme S22. Synthesis of 4-octadecyl benzyl chloride **13**

Procedure: In an oven-dried 100 mL Schlenk flask, methyl 4-chlorobenzoate (1.08 g, 5.00 mmol) was dissolved into 20 mL dry THF under an argon atmosphere. $Fe(acac)_3$ (88.3 mg, 250 μmol, 5 mol%) and NMP (5.79 mL, 60.0 mmol) were added subsequently. After cooling to 0 °C with an ice-water cooling bath, octadecylmagnesium chloride solution (12.0 mL, 6.00 mol, 0.5 M in THF) was added dropwise into the reaction mixture. After being vigorously stirred at 0 °C for 30 min, the reaction was quenched with NH_4Cl (saturated aq. solution). The biphasic mixture was extracted 3 times with EtOAc, and the combined organic phase was dried over anhydrous Na_2SO_4. The solvent was removed on a rotavap to afford the crude product which was then purified by column chromatography. The product **S3** was obtained as a white solid (1.40 g, 72% yield).

The ^1H&^{13}C NMR spectra of ***S3*** *were in good agreement with the reported ones.*[17]

(4-octadecylphenyl)methanol (S4)

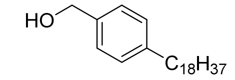

Chemical Formula: $C_{25}H_{44}O$
Molecular Weight: 360.6260

$LiAlH_4$ (274 mg, 7.20 mmol) was weighed into an oven-dried 50 mL three-neck flask, Et_2O (10 mL) was then added via a syringe under an argon atmosphere. After cooling to 0 °C with an ice-water bath, a solution of **S3** (1.40 g, 3.60 mmol) in 10 mL Et_2O was added dropwise with vigorous stirring. The reaction was allowed to continue for 30 min at 0 °C before being warmed to room temperature for further 2 hours. After the full consumption of **S3**, 3.00 mL NaOH (6.0 M aq.) was added slowly to quench the reaction. The resulting grey suspension was filtered through a short pad of Celite. The filter cake was washed 5 times with hot EtOAc. The filtrate was then combined and concentrated on a rotavap. The crude product was purified by column chromatography to afford **S4** as a white solid (1.24 g, 95% yield).

White solid, 95% yield;

^{1}H NMR (600 MHz, $CDCl_3$) δ (ppm) 7.28 (d, J = 7.8 Hz, 2H), 7.18 (d, J = 7.9 Hz, 2H), 4.65 (d, J = 4.3 Hz, 2H), 2.62 – 2.58 (m, 2H), 1.66 (s, 1H), 1.62 – 1.58 (m, 2H), 1.31 – 1.25 (m, 30H), 0.89 (t, J = 7.0 Hz, 3H).

^{13}C NMR (150 MHz, $CDCl_3$) δ (ppm) 142.5, 138.1, 128.6, 127.1, 65.3, 63.1, 35.7, 32.8, 31.9, 31.5, 29.7, 29.7, 29.7, 29.7, 29.6, 29.6, 29.5, 29.4, 29.4, 29.3, 25.7, 22.7, 14.1.

HRMS (ESI) for: $C_{25}H_{44}OAg$ $[M+Ag]^+$: calcd 467.2438, found 467.2432.

1-(chloromethyl)-4-octadecylbenzene (13)

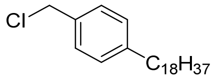

Chemical Formula: $C_{25}H_{43}Cl$
Molecular Weight: 379.0690

In an oven-dried 50 mL flask, intermediate **S4** (1.24g, 3.44 mmol) was dissolved into 20 mL dry DCM under an argon atmosphere. The mixture was then cooled to 0 ºC with an ice-water cooling bath. After stirring at this temperature for 5 min, thionyl chloride (300 μL, 4.13 mmol) was added dropwise. The reaction was stirred for a further 2 hours and monitored by TLC. After the full consumption of the material, all volatiles were directly removed under vacuum. The remaining residual was then dissolved into 20 mL DCM and washed with saturated $NaHCO_3$ solution and brine. After concentration on a rotavap, the crude product was then purified by column chromatography to afford the desired product as a white solid (1.22g, 94% yield).

White solid, 94% yield;

^{1}H NMR (600 MHz, $CDCl_3$) δ (ppm) 7.30 (d, J = 7.9 Hz, 2H), 7.17 (d, J = 7.9 Hz, 2H), 4.58 (s, 2H), 2.62 – 2.58 (m, 2H), 1.63 – 1.58 (m, 2H), 1.26 (d, J = 3.2 Hz, 30H), 0.89 (t, J = 7.0 Hz, 3H).

^{13}C NMR (150 MHz, $CDCl_3$) δ (ppm) 143.4, 134.7, 128.8, 128.5, 46.3, 35.7, 31.9, 31.4, 29.7, 29.7, 29.7, 29.6, 29.5, 29.4, 29.3, 22.7, 14.1.

HRMS (ESI) for: $C_{25}H_{43}ClAg$ $[M+Ag]^+$: calcd 485.2099, found 485.2093.

7.2 Friedel–Crafts reactions catalyzed by capsule/cage

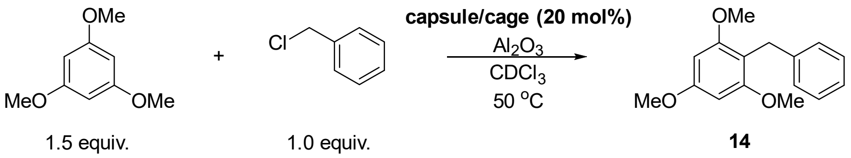

Procedure: ***trans*-2b** (30.0 µmol, 22.4 mg, equal to 5.00 µmol of cage **II**) and Al_2O_3 basic (50.0 mg) were weighed into a 2.5 mL screw-cap glass vial, and 500 µL $CDCl_3$ was added, the mixture was homogenized by careful sonicating and shaking. 1,3,5-Trimethoxybenzene (37.5 µmol, 6.31 mg) was added followed by benzyl chloride (25.0 µmol, 2.99 µL). After adding TES (10.0 µmol as the internal standard), the reaction mixture was placed in a 50 °C heating block. The material conversion and product formation were monitored by ^{1}H NMR. The results are summarized in the following table and graphic:

Time (h)	0	15	45	75	105	135	165	195
Bn-Cl **12** (%)	100	60	23	13	9	6	5	4
Product **14** (%)	0	43	57	58	61	63	64	64

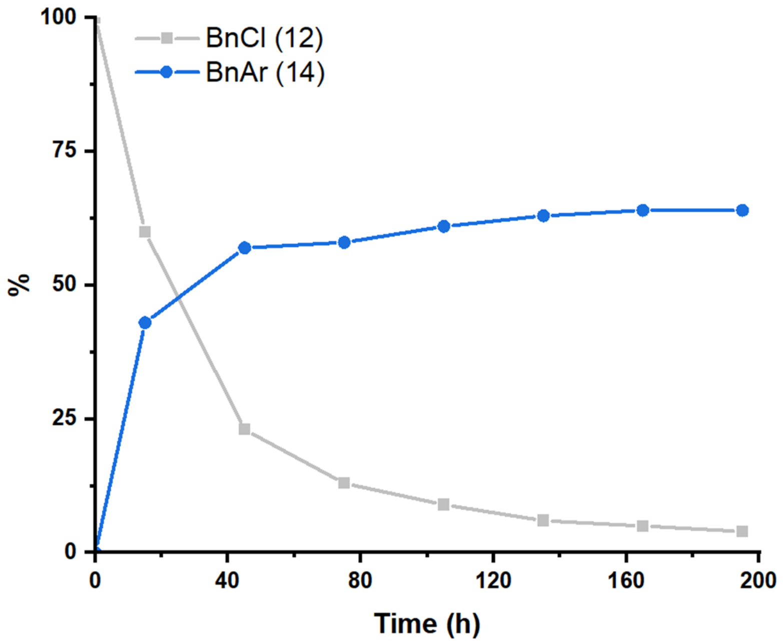

As a comparison, the reaction between 1,3,5-trimethoxybenzene and benzyl chloride catalyzed by *C11*-resorcin[4]arene capsule **I** (30.0 µmol, 33.2 mg) was performed. The results are summarized in the following table and graphic:

Time (h)	0	2	4	8	12	16	24	36	48	72	96
Bn-Cl (%)	100	56	37	24	17	16	12	9	7	6	6
Product **14** (%)	0	28	41	44	46	47	49	49	52	51	51

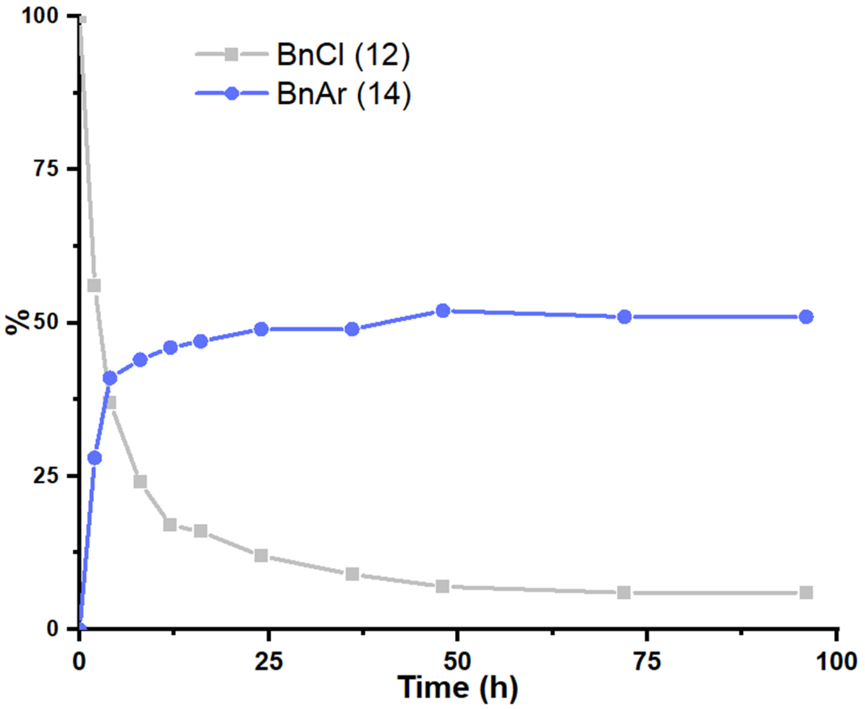

Note: *F-C product **14** is a known compound. Its ^{1}H and ^{13}C NMR spectra were in good agreement with the reported one.*[18]

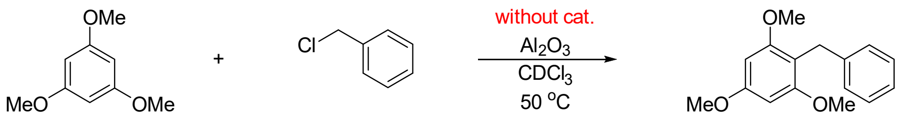

1.5 equiv. **1.0 equiv.** **14**

The background reaction between 1,3,5-trimethoxybenzene and benzyl chloride was performed without adding any catalyst, but otherwise under identical reaction conditions. No background reaction was observed in the absence of the capsule/cage. The results are summarized in the following table and graphic:

Time (h)	0	24	48	72	96	120	144	168	192
BnCl (%)	100	92	90	86	84	71	67	63	61
Product **14** (%)	0	0	0	0	0	0	0	0	0

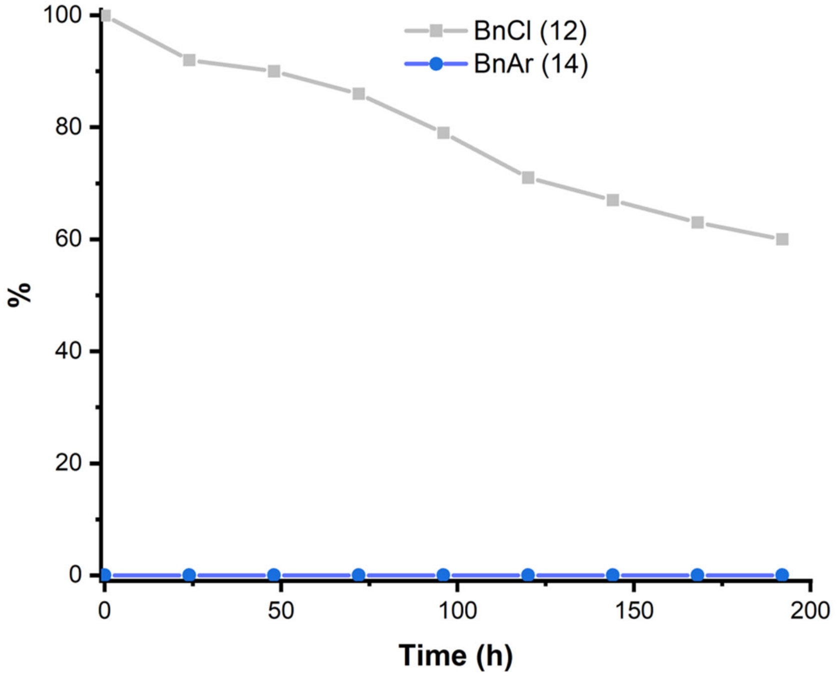

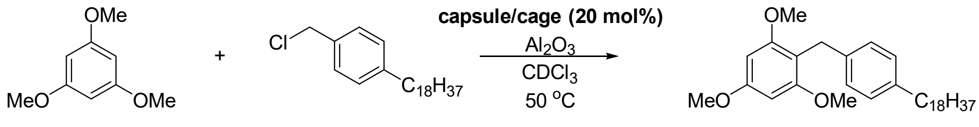

1.5 equiv. **13** 1.0 equiv. **15**

Procedure: ***trans*-2b** (30.0 µmol, 22.4 mg, equal to 5.00 µmol hexameric cage) and Al_2O_3 basic (50.0 mg) were weighed into a 2.5 mL screw cap glass vial and 500 µL $CDCl_3$ was added, the mixture was homogenized by careful sonicating and shaking. 1,3,5-Trimethoxybenzene (37.5 µmol, 6.31 mg) was added followed by 4-octadecyl benzyl chloride (25.0 µmol, 10.6 mg). After adding TES (10.0 µmol as the internal standard), the reaction mixture was placed in a 50 °C heating block and monitored with NMR. The results are summarized in the following table and graphic:

Time (h)	0	24	48	72	96	120	144	168
Substrate **13** (%)	100	49	23	16	10	9	9	9
Product **15** (%)	0	43	53	56	59	63	67	68

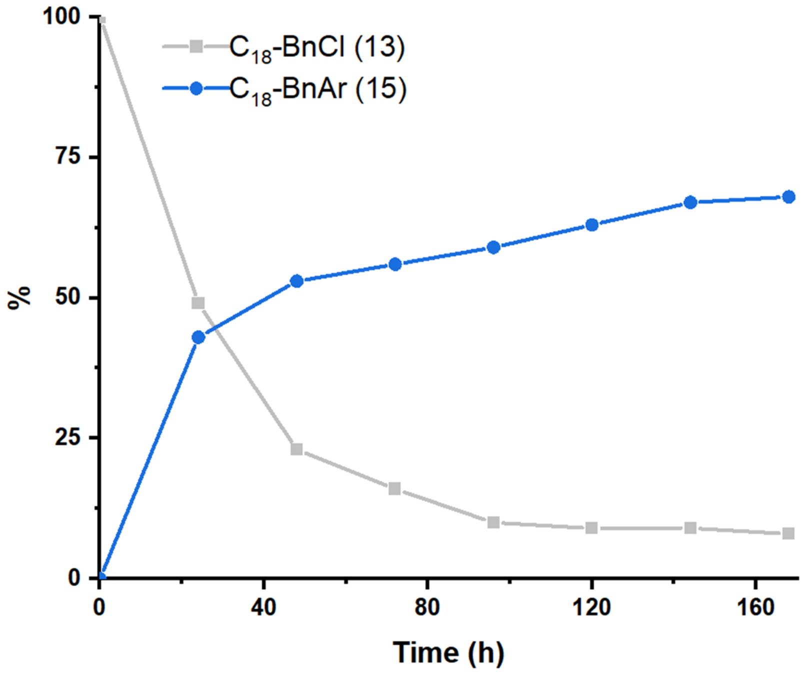

To isolate product **15** for characterization, a new reaction was set up at a 50.0 µmol scale. The product was isolated as a white solid (16.8 mg, 65% yield) after 7 days.

1,3,5-trimethoxy-2-(4-octadecylbenzyl)benzene (15)

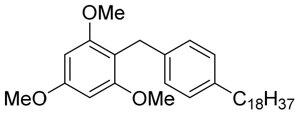

Chemical Formula: $C_{34}H_{54}O_3$
Molecular Weight: 510.80

White solid, 65% yield;

^{1}H NMR (600 MHz, $CDCl_3$) δ 7.13 (d, J = 7.8 Hz, 2H), 7.02 (d, J = 7.9 Hz, 2H), 6.15 (s, 2H), 3.89 (s, 2H), 3.81 (s, 3H), 3.78 (s, 6H), 2.54 – 2.50 (m, 2H), 1.55 (d, J = 4.3 Hz, 2H), 1.25 (d, J = 6.2 Hz, 30H), 0.88 (t, J = 7.0 Hz, 3H).

^{13}C NMR (150 MHz, $CDCl_3$) δ (ppm) 159.5, 158.8, 139.7, 139.3, 128.2, 128.0, 110.4, 90.5, 55.7, 55.3, 35.6, 31.9, 31.6, 29.7, 29.7, 29.7, 29.6, 29.5, 29.4, 29.4, 27.8, 22.7, 14.1.

ESI-MS for: $C_{34}H_{55}O_3$ [M+H]$^+$: calcd 511.41, found 511.30;

HRMS (ESI) for: $C_{34}H_{54}O_3Na$ [M+Na]$^+$: calcd 533.3965, found 533.3960.

As a comparison, the reaction between 1,3,5-trimethoxybenzene and 4-octadecyl benzyl chloride catalysed by C*11*-resorcin[4]arene (30.0 µmol, 33.2 mg) was performed. The results are summarized in the following table and graphic:

Time (h)	0	24	48	72	96	120	144	168
Substrate **13** (%)	100	70	59	49	43	33	26	20
Product **15** (%)	0	1	1	3	5	7	9	11

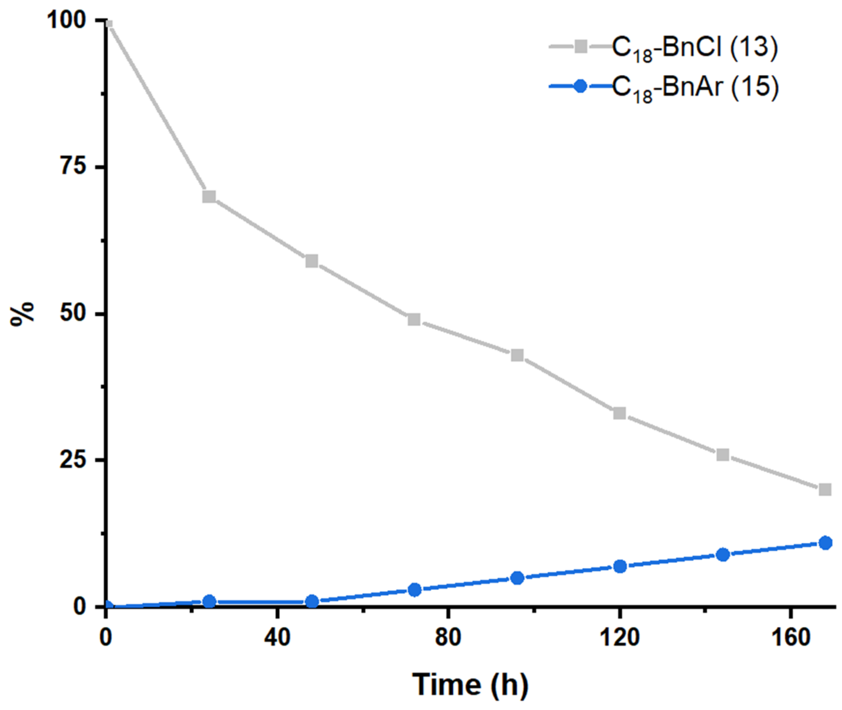

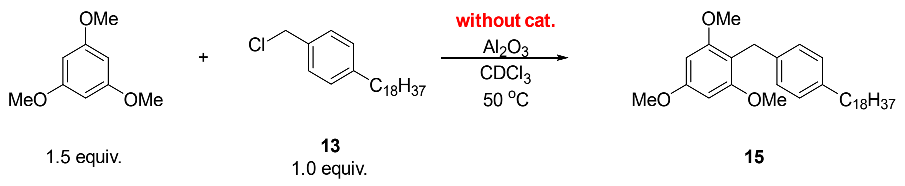

The background reaction between 1,3,5-trimethoxybenzene and 4-octadecyl benzyl chloride was performed without adding any catalyst, but otherwise under identical reaction conditions. The results are summarized in the following table and graphic:

Time (h)	0	24	48	72	96	120	144	168
Substrate **13** (%)	100	59	42	33	28	25	24	23
Product **15** (%)	0	1	2	3	3	4	4	4

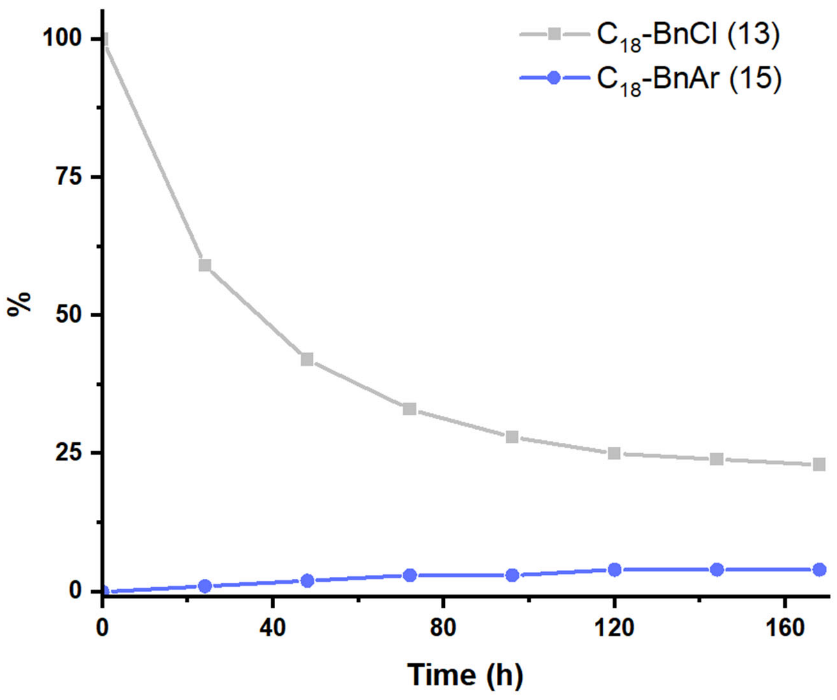

7.3 Control experiments

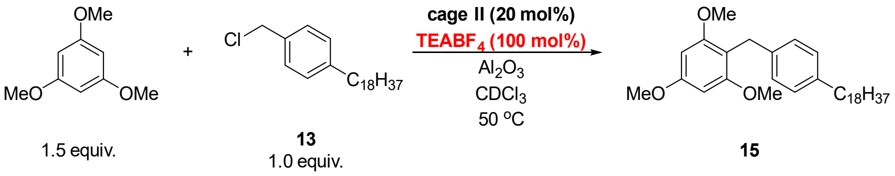

A control experiment with the addition of 1.0 equivalent of tetraethylammonium tetrafluoroborate (TEABF4) as the inhibitor for molecular capsules was performed, with 1,3,5-trimethoxybenzene and 4-octadecyl benzyl chloride **13** as reaction partners, and capsule **I** as the catalyst. The results are summarized in the following table and graphic:

Time (h)	0	24	48	72	96	120	144	168
Substrate **13** (%)	100	86	86	85	86	86	84	83
Product **15** (%)	0	0	0	0	0	0	0	0

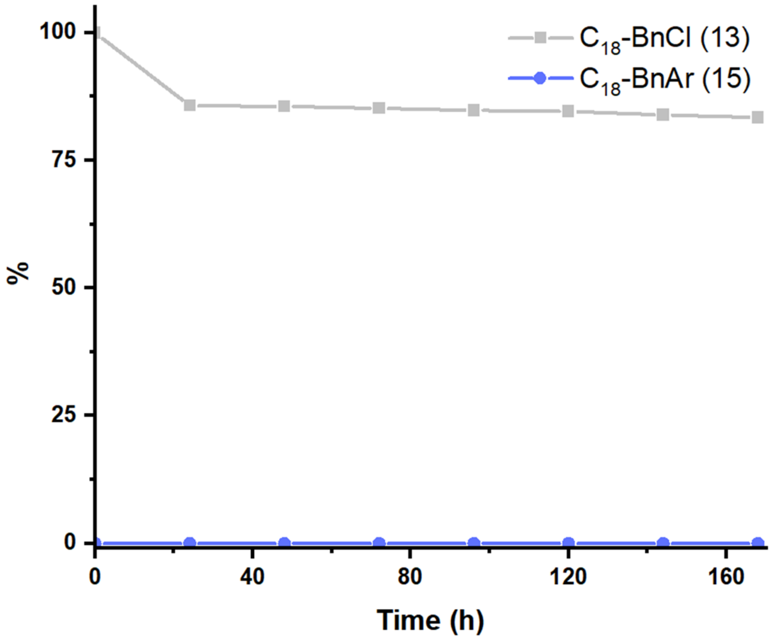

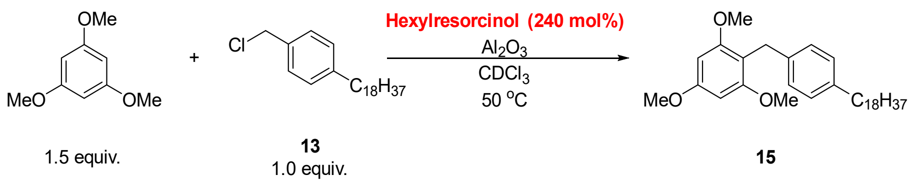

A control experiment by using 2.4 equivalent hexylresorcinol as the subunit of cage **II**, instead of cage **II** was performed, with 1,3,5-trimethoxybenzene and 4-octadecyl benzyl chloride as the reaction partners. Although the majority of 4-octadecyl benzyl chloride **13** was converted in 8 days, presumably consumed by hexylresorcinol, no F-C product **15** was detected.

The results are summarized in the following table and graphic:

Time (h)	0	24	48	72	96	120	144	168	192
Substrate **13** (%)	100	53	32	22	19	18	18	18	17
Product **15** (%)	0	0	0	0	0	0	0	0	0

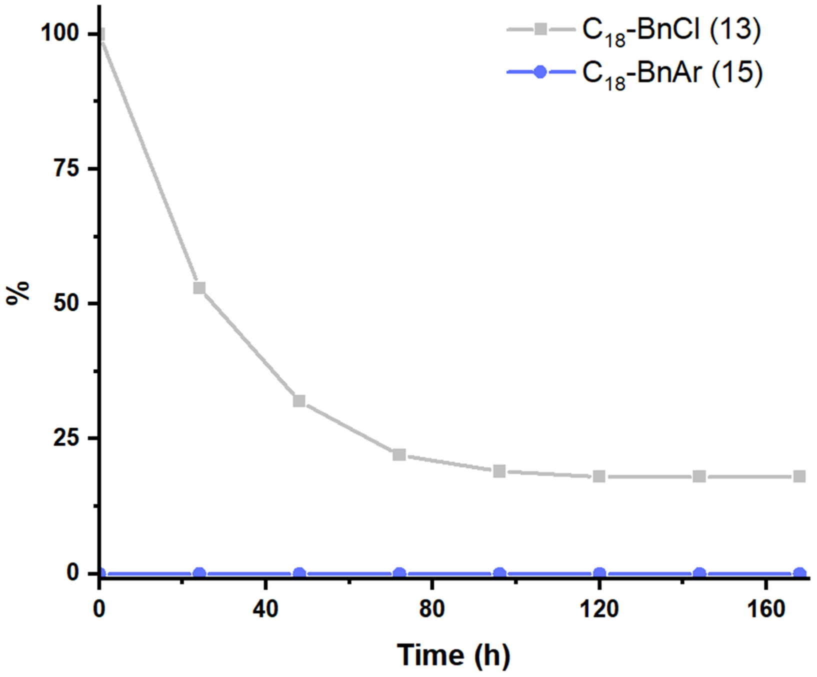

8. References

[1] Wu, D. H., Chen, A. D., Johnson, C. S. *Journal of Magnetic Resonance, Series A* **1995**, *115*, 260-264.

[2] Ran, L.; Li, H.; Chao, G.; Kang, X.; Lei, T.; Li. W. *Synlett* **2020**, *31*, 1809-1812.

[3] Tsui, E. Y.; Muller, P.; Sadighi, J. P. *Angew. Chem. Int. Ed.* **2008**, *47*, 8937-8940.

[4] Wørmer, G. J.; Villadsen, N. L.; Nørby, P.; Poulsen, T. B. *Angew. Chem. Int. Ed.* **2021**, *60*, 10521-10525.

[5] Chen, H. C.; Chen, S. H. *J. Phys. Chem.* **1984**, *88*, 51185121.

[6] Beaudoin, D.; Rominger, F.; Mastalerz, M. *Angew. Chem. Int. Ed.* **2016**, *55*, 15599-15603.

[7] Laio, A.; Parrinello, M.; *Proc. Natl. Acad. Sci. U. S. A.* **2002**, *99*, 12562–12566.

[8] Barducci, A.; Bussi, G.; Parrinello, M. *Phys. Rev. Lett.* **2008**, *100,* 020603.

[9] Berendsen, H. J. C.; Spoel, D.; Drunen, R. *Comput. Phys. Commun.* **1995**, *91*, 43–56.

[10] Tribello, G. A.; Bonomi, M.; Branduardi, D.; Camilloni, C.; Bussi, G. *Comput. Phys. Commun.* **2014**, *185*, 604–613.

[11] Bonomi, M.; Bussi, G.; Camilloni, C.; Tribello, G. A.; Banáš, P.; Barducci, A.; Bernetti, M.; Bolhuis, P. G.; Bottaro, S.; Branduardi, D.; Capelli, R.; Carloni, P.; Ceriotti, M.; Cesari, A.; Chen, H.; Chen, W.; Colizzi, F.; De, S.; Pierre, M.; Donadio, D.; Drobot, V.; Ensing, B.; Ferguson, A. L.; Filizola, M.; Fraser, J. S.; Fu, H.; Gasparotto, P.; Gervasio, F. L.; Giberti, F.; Gil-Ley, A.; Giorgino, T.; Heller, G. T.; Hocky, G. M.; Iannuzzi, M.; Invernizzi, M.; Jelfs, K. E.; Jussupow, A.; Kirilin, E.; Laio, A.; Limongelli, V.; Lindorff-Larsen, K.; Löhr, T.; Marinelli, F.; Martin-Samos, L.; Masetti, M.; Meyer, R.; Michaelides, A.; Molteni, C.; Morishita, T.; Nava, M.; Paissoni, C.; Papaleo, E.; Parrinello, M.; Pfaendtner, J.; Piaggi, P.; Piccini, G. M.; Pietropaolo, A.; Pietrucci, F.; Pipolo, S.; Provasi, D.; Quigley, D.; Raiteri, P.; Raniolo, S.; Rydzewski, J.; Salvalaglio, M.; Sosso, G. C.; Spiwok, V.; Šponer, J.; Swenson, D. W. H.; Tiwary, P.; Valsson, O.; Vendruscolo, M.; Voth, G. A.; White, A. *Nat. Methods* **2019**, **16**, 670–673.

[12] Wang, J.; Wolf, R. M.; Caldwell, J. W.; Kollman, P. A.; Case, D. A. *J. Comput. Chem.* **2004**, *25*, 1157–1174.

[13] Woods, R. J.; Chappelle, R. *J. Mol. Struc-THEOCHEM* **2000**, *527*, 149–156.

[14] Bussi, G.; Donadio, D.; Parrinello, M. *J. Chem. Phys.* **2007**, *126*, 14101-14107.

[15] Parrinello, M.; Rahman, A.; Parrinello, M.; Rahman, A. *J. Appl. Phys.* **1981**, *52*, 7182–7190.

[16] Humphrey, W.; Dalke, A.; Schulten, K. *J. Mol. Graph.* **1996**, *14*, 33–38.

[17] Feng, W.; Liu, T.; Wang, D.; Wang, P.; Wu, Y. *Angew. Chem. Int. Ed.*, **2020**, *59*, 20399-20404.

[18] Parnes, R.; Pappo, D. *Org. Lett.* **2015**, *17*, 2924-2927.

9. NMR-Spectra of New Compounds

1,5-bis(2,4-bis(benzyloxy)phenyl)penta-1,4-dien-3-one **(7)**

^{1}H NMR ($CDCl_3$, 600 MHz, 25 ºC)

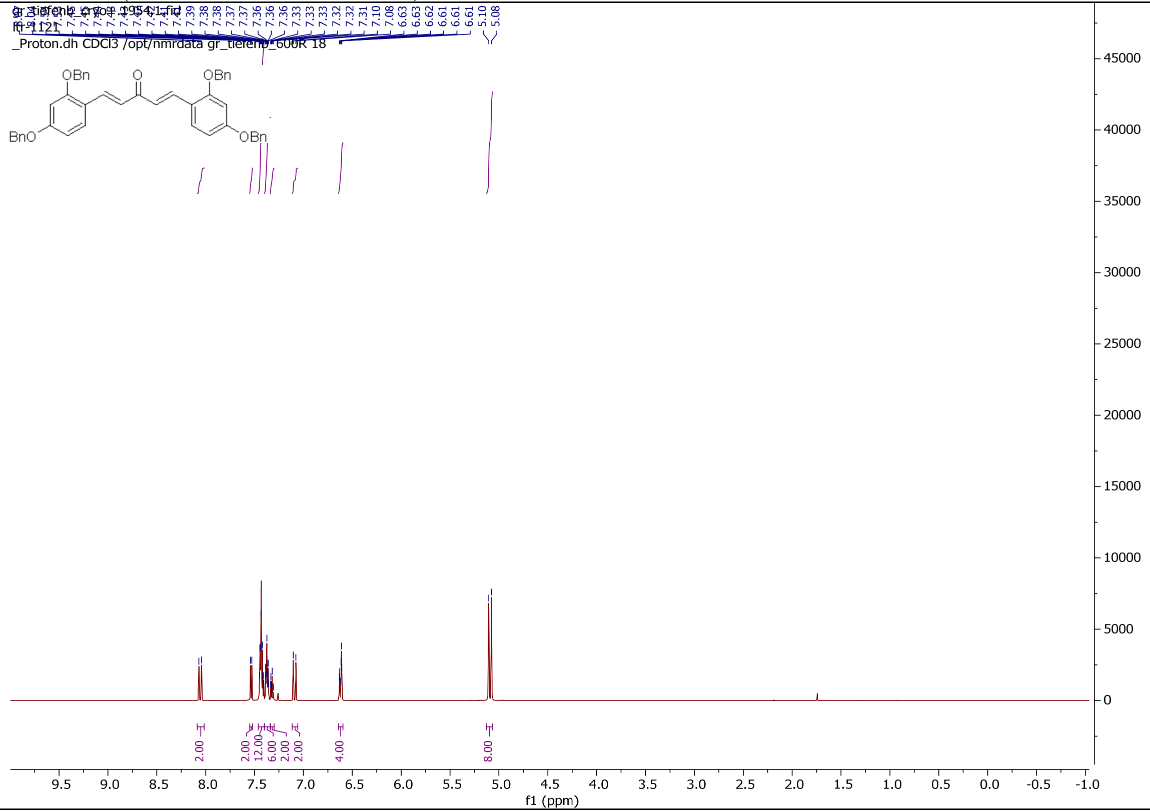

^{13}C NMR ($CDCl_3$, 150 MHz, 25 ºC)

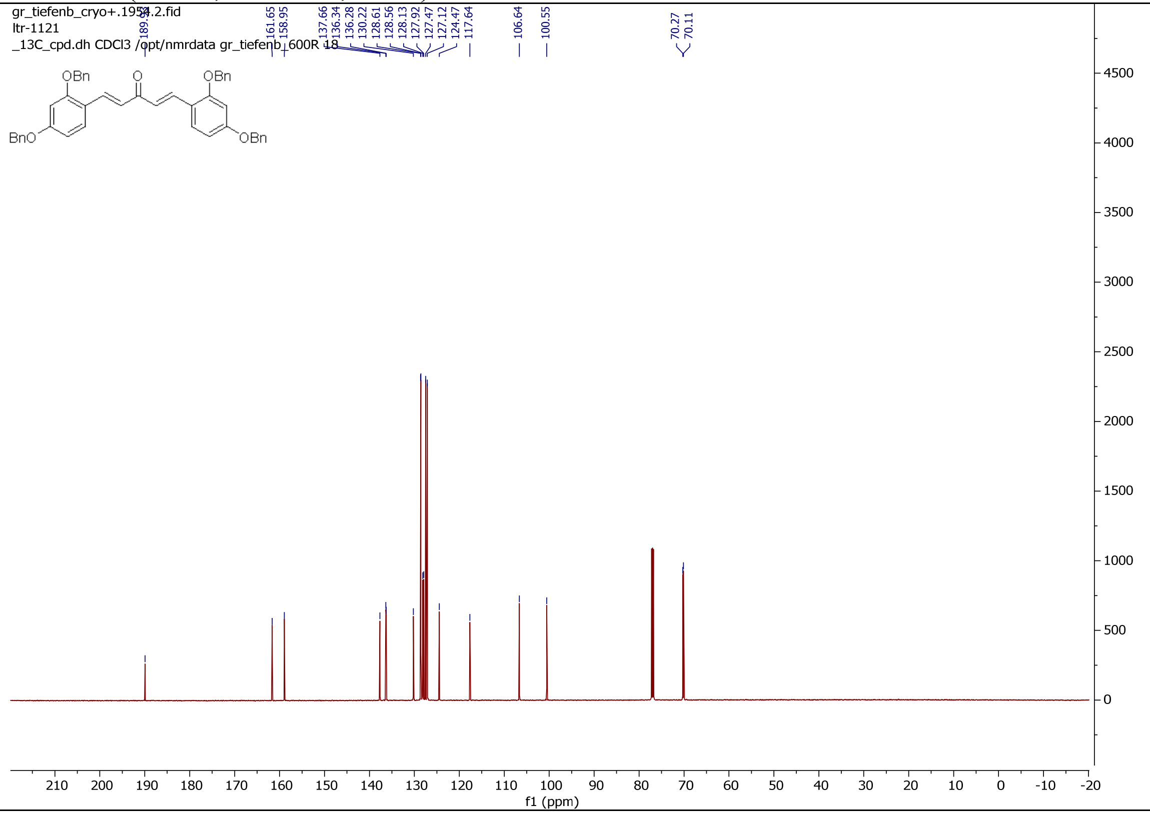

1,5-Bis(2,4-bis(benzyloxy)phenyl)pentan-3-one **(3)**

^{1}H NMR ($CDCl_3$, 500 MHz, 25 °C)

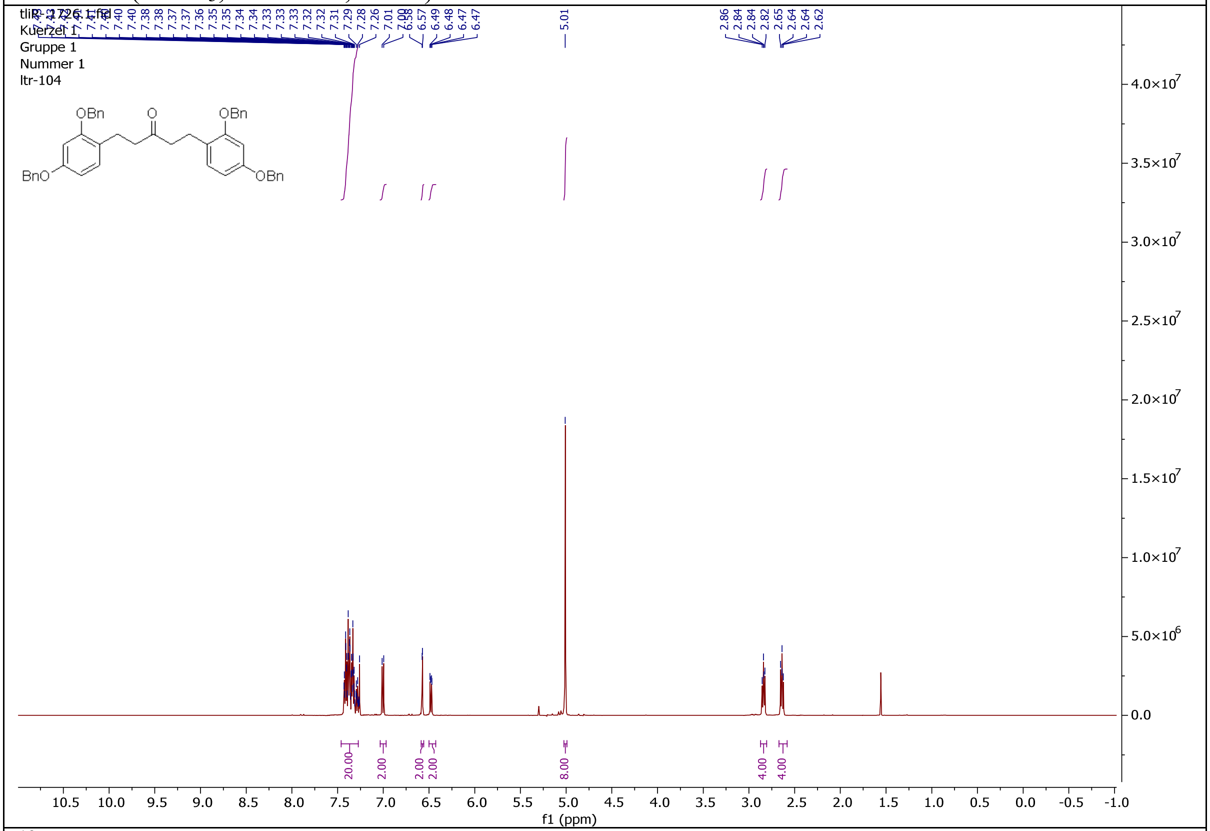

^{13}C NMR ($CDCl_3$, 125 MHz, 25 °C)

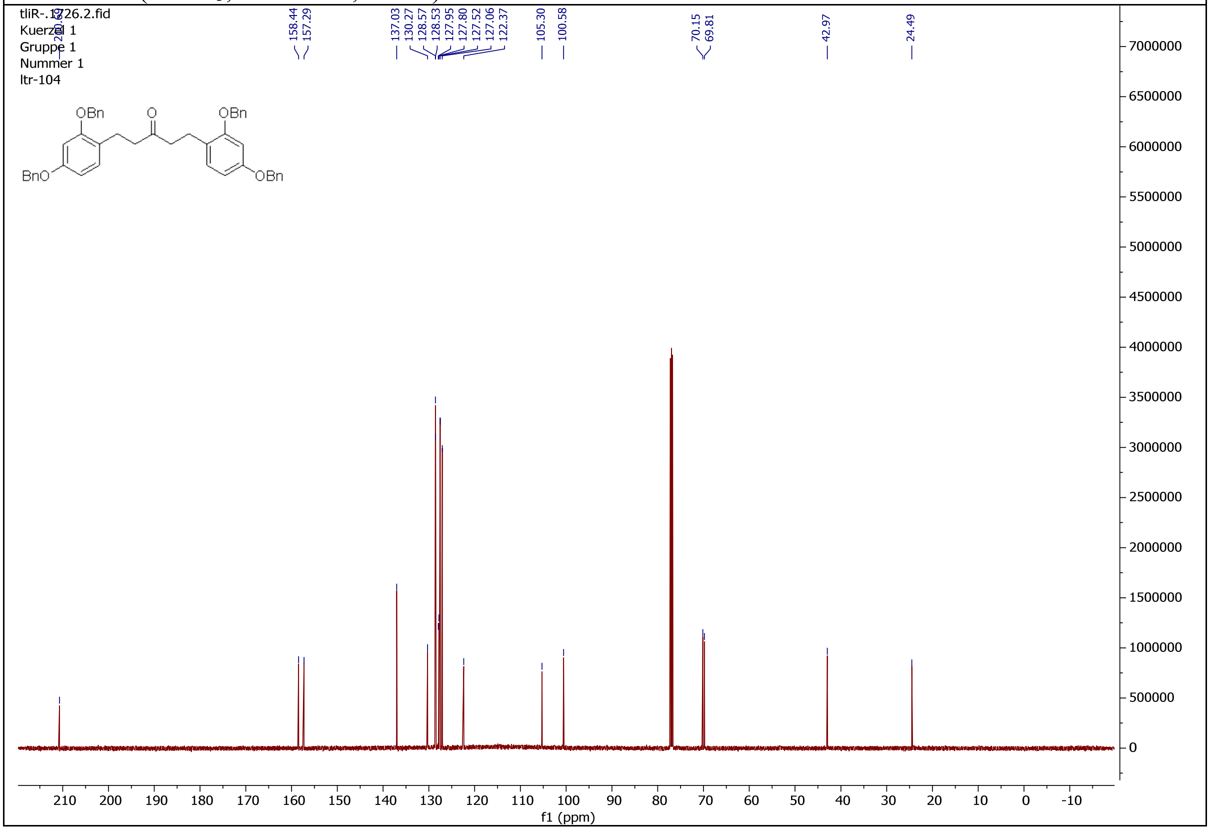

1,1'-(4,6-dihydroxy-1,3-phenylene)bis(3-methylbutan-1-one) **(9a)**

^{1}H NMR ($CDCl_3$, 500 MHz, 25 ºC)

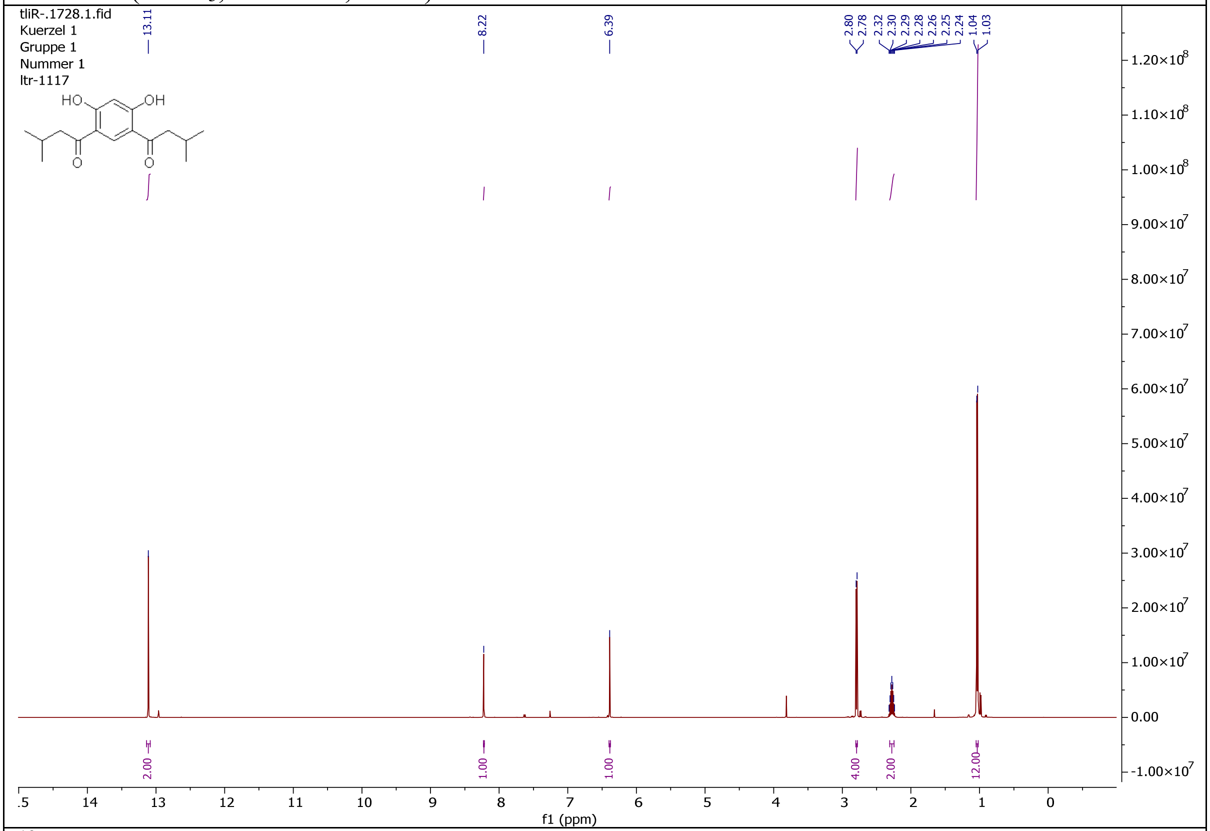

^{13}C NMR ($CDCl_3$, 125 MHz, 25 ºC)

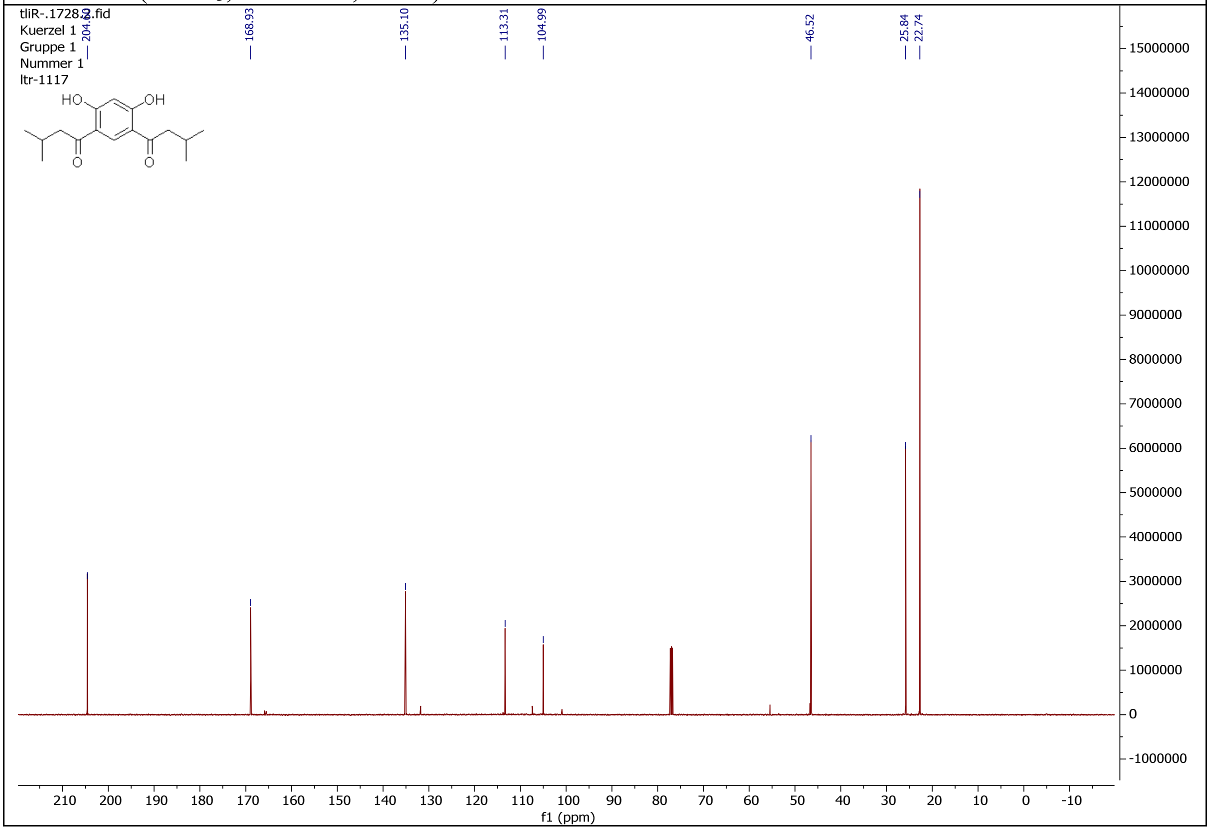

1,1'-(4,6-dihydroxy-1,3-phenylene)bis(dodecan-1-one) **(9b)**

^{1}H NMR ($CDCl_3$, 600 MHz, 25 °C)

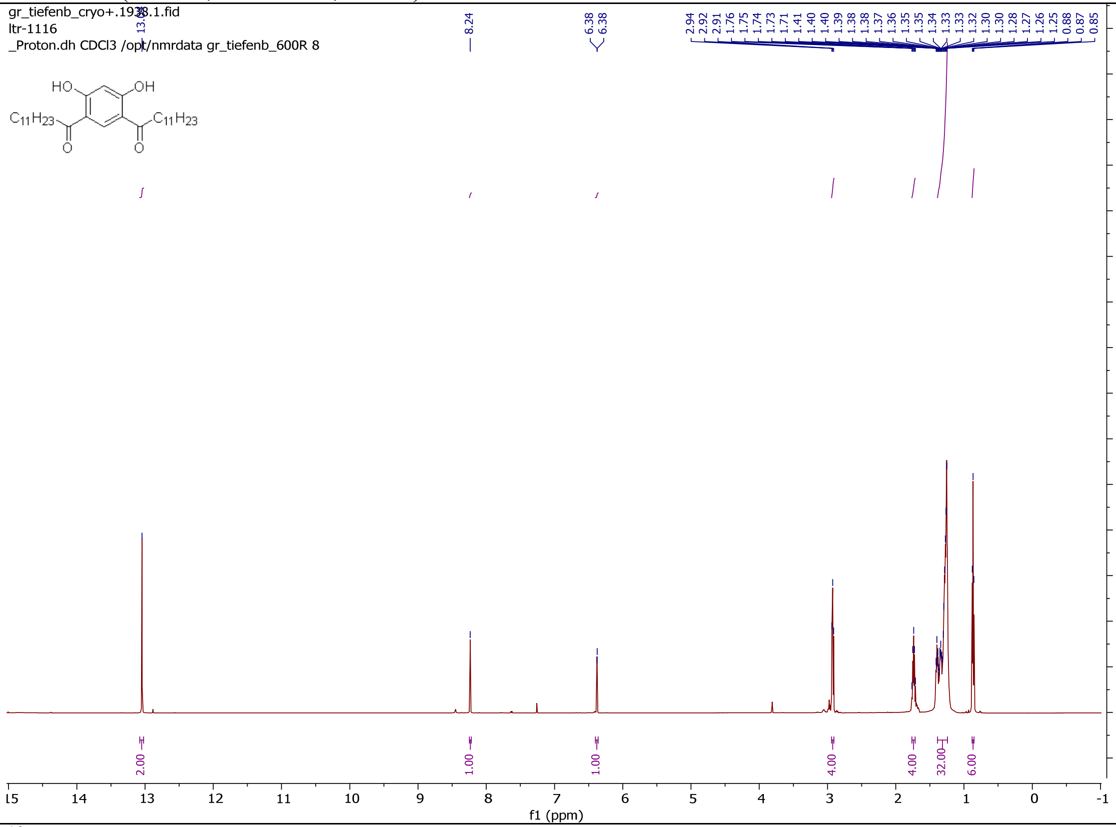

^{13}C NMR ($CDCl_3$, 150 MHz, 25 °C)

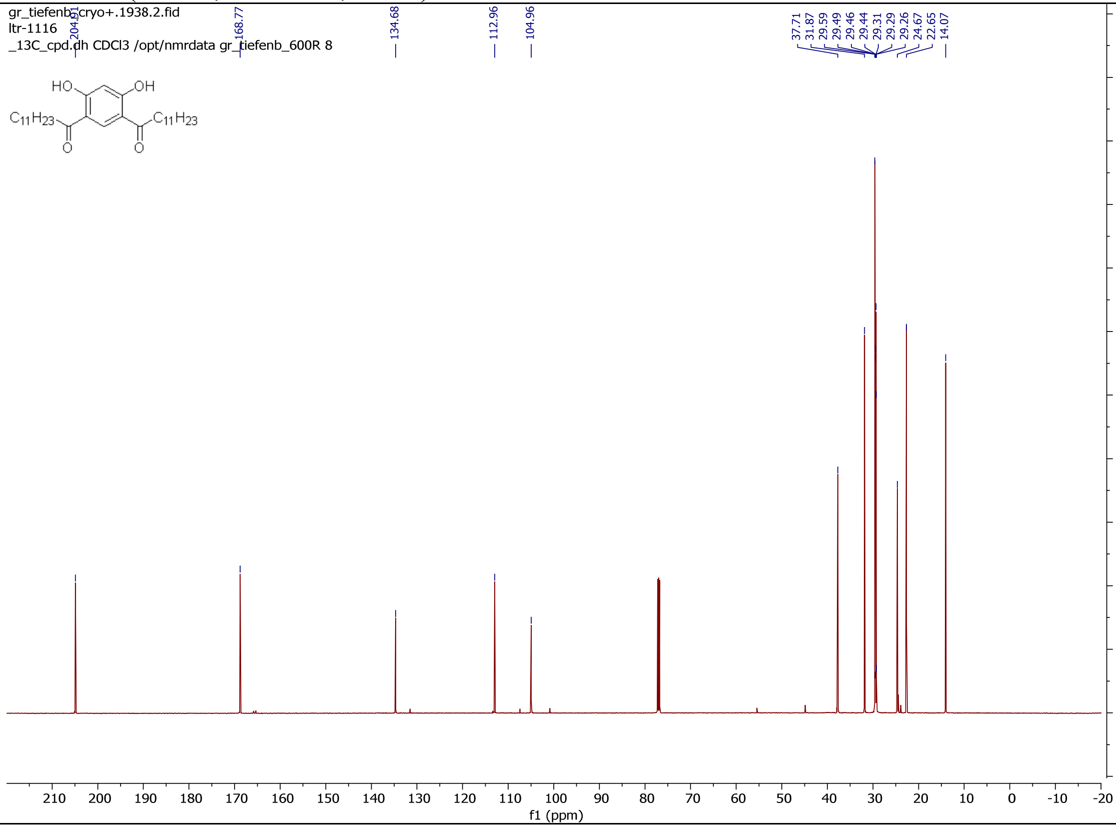

1,1'-(4,6-bis(benzyloxy)-1,3-phenylene)bis(3-methylbutan-1-one) **(10a)**

^{1}H NMR ($CDCl_3$, 600 MHz, 25 °C)

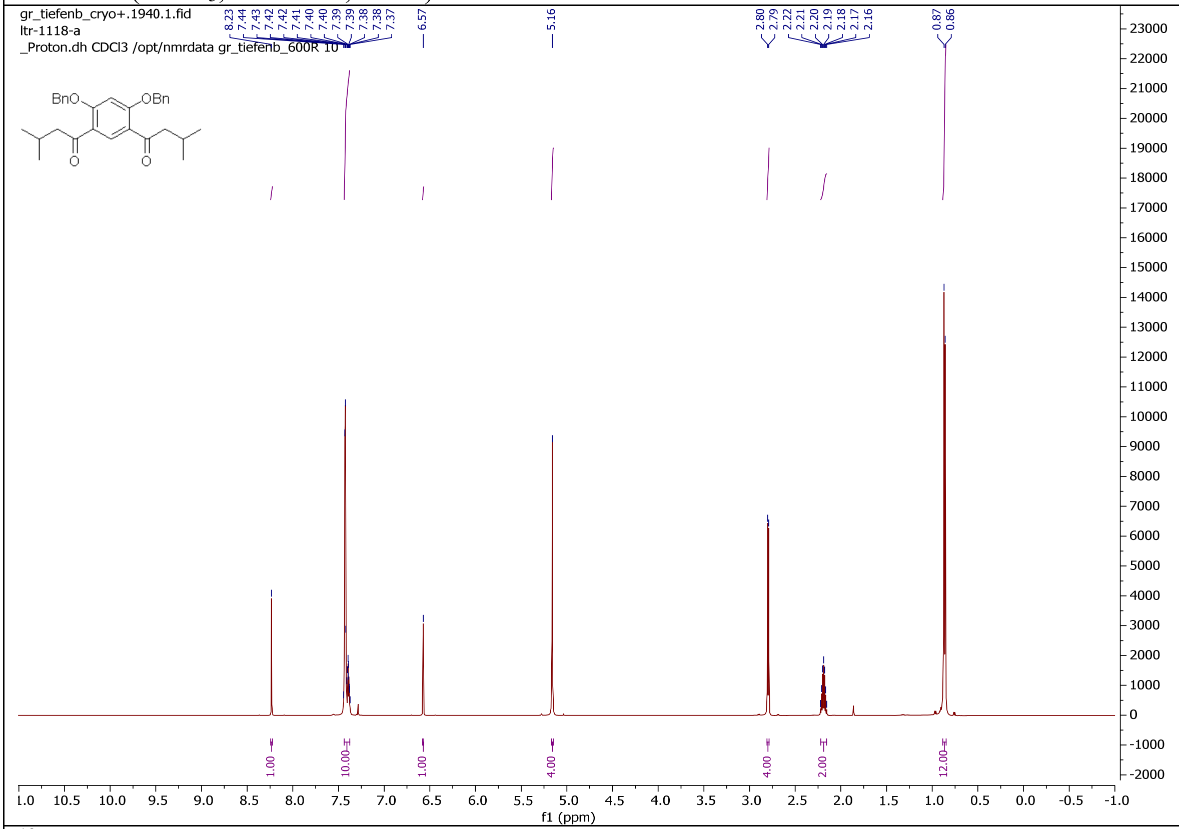

^{13}C NMR ($CDCl_3$, 150 MHz, 25 °C)

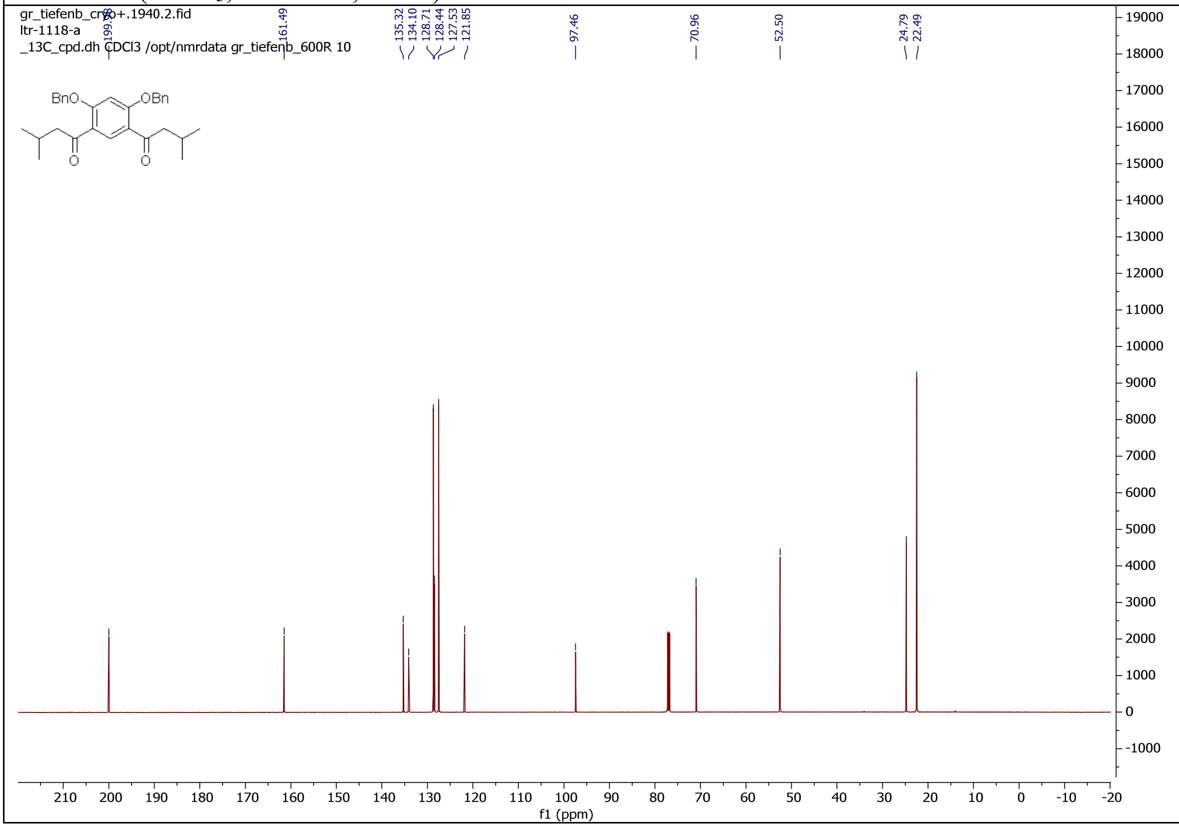

1,1'-(4,6-bis(benzyloxy)-1,3-phenylene)bis(dodecan-1-one) **(10b)**

^{1}H NMR ($CDCl_3$, 600 MHz, 25 °C)

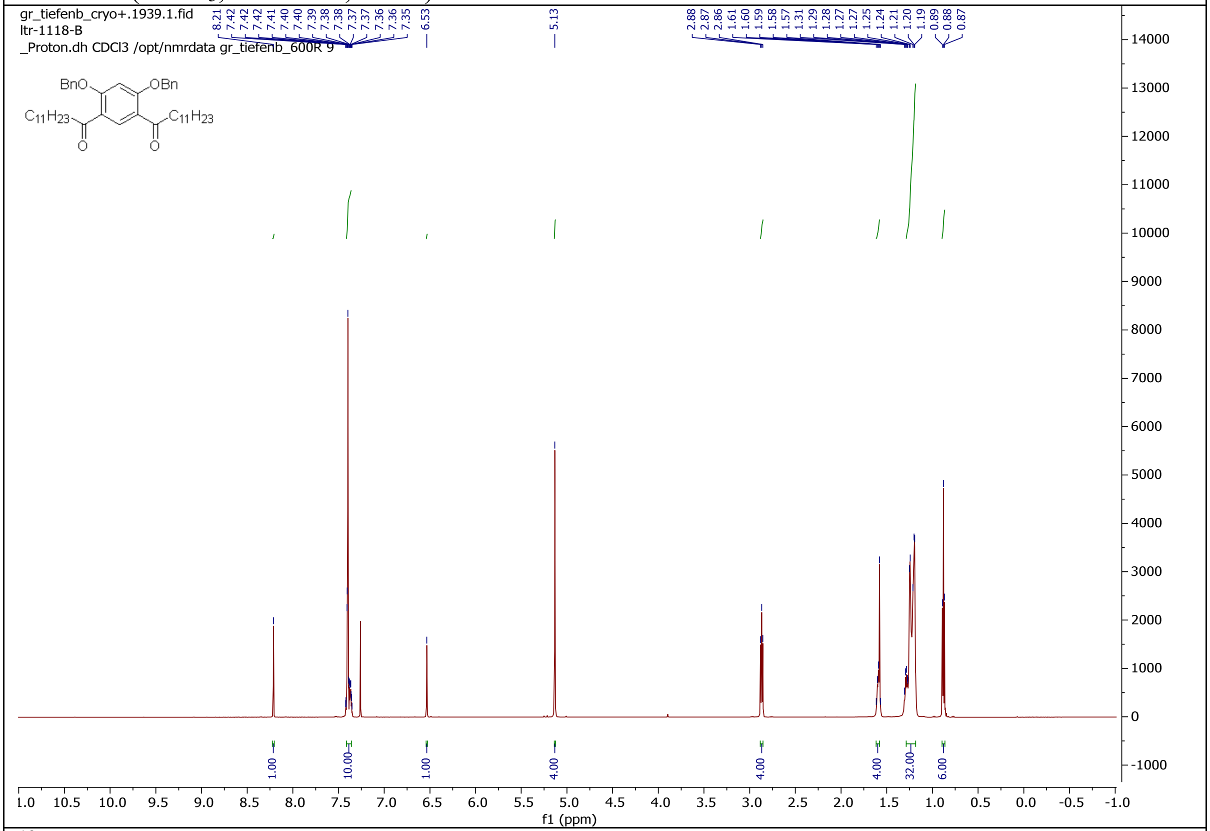

^{13}C NMR ($CDCl_3$, 150 MHz, 25 °C)

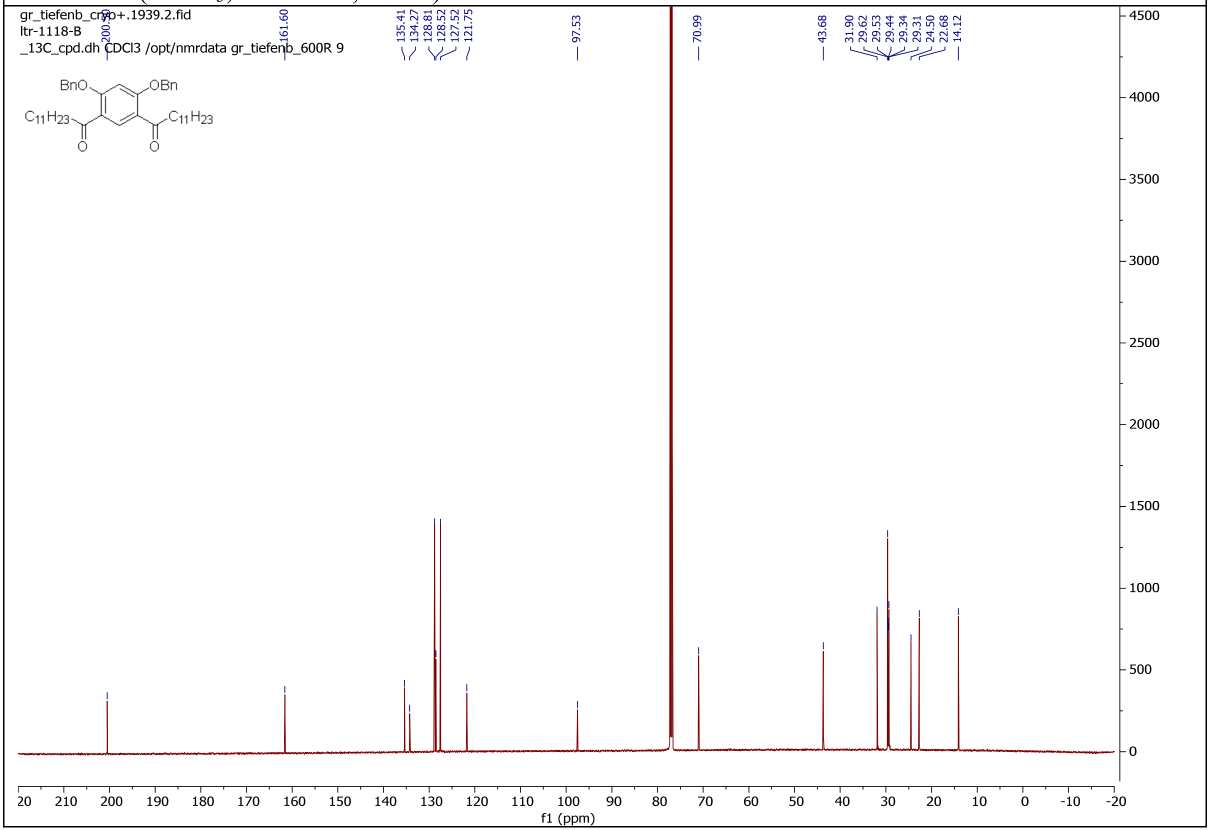

1,1'-(4,6-bis(benzyloxy)-1,3-phenylene)bis(3-methylbutan-1-ol) **(4a)**

^{1}H NMR ($CDCl_3$, 500 MHz, 25 °C)

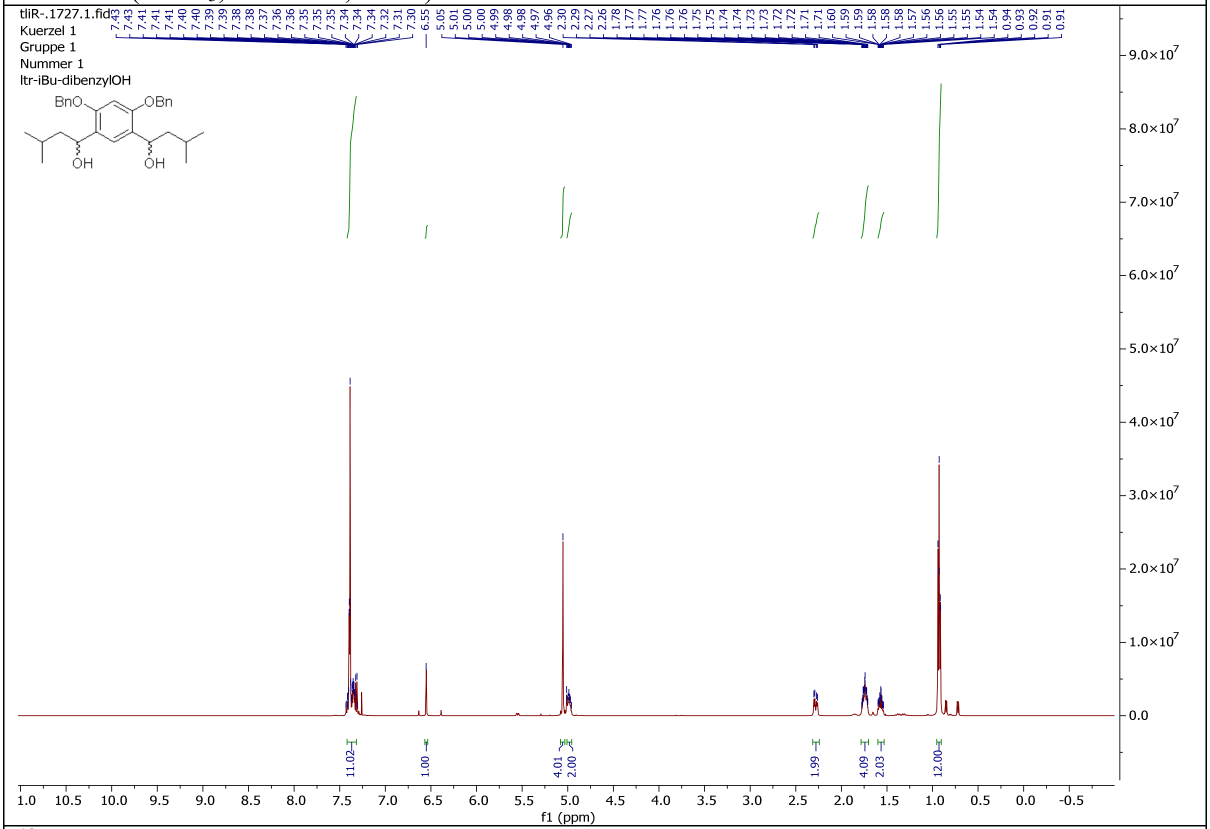

^{13}C NMR ($CDCl_3$, 125 MHz, 25 °C)

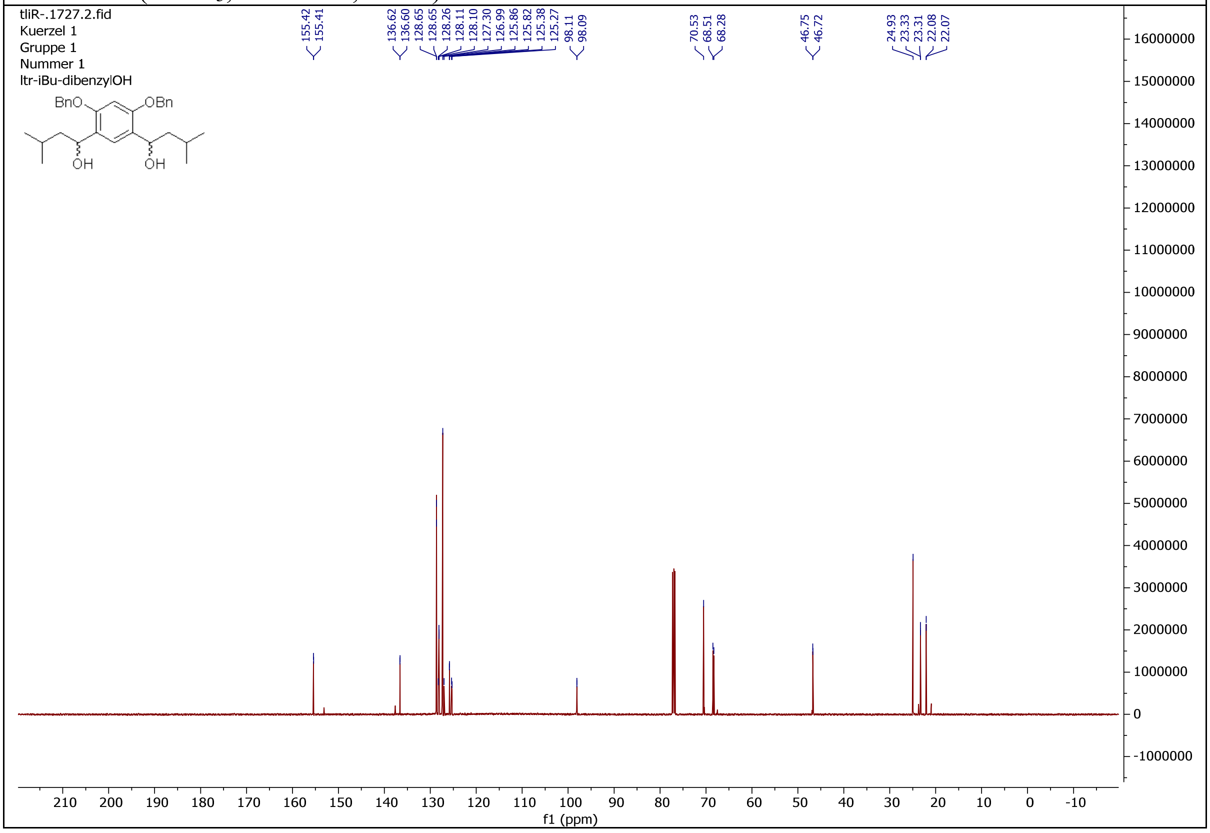

1,1'-(4,6-bis(benzyloxy)-1,3-phenylene)bis(dodecan-1-one) **(4b)**

^{1}H NMR ($CDCl_3$, 600 MHz, 25 ºC)

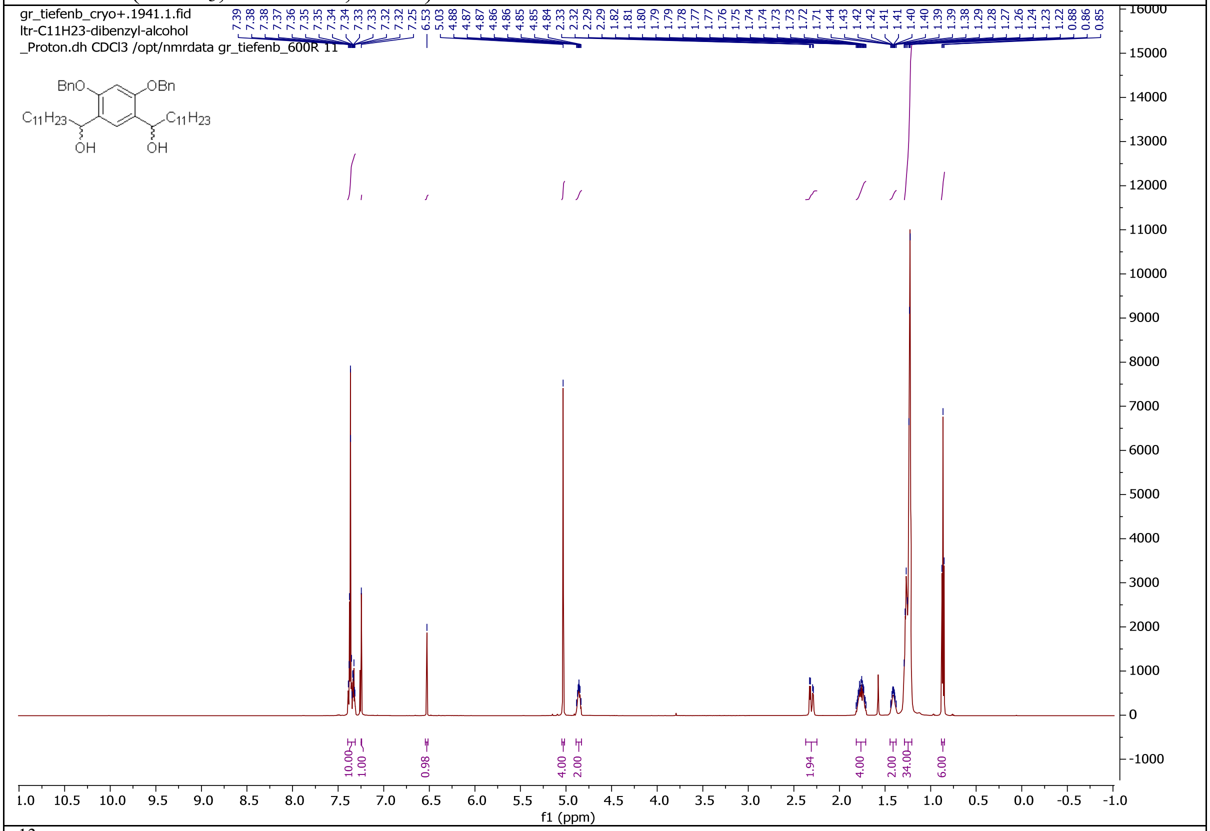

^{13}C NMR ($CDCl_3$, 150 MHz, 25 ºC)

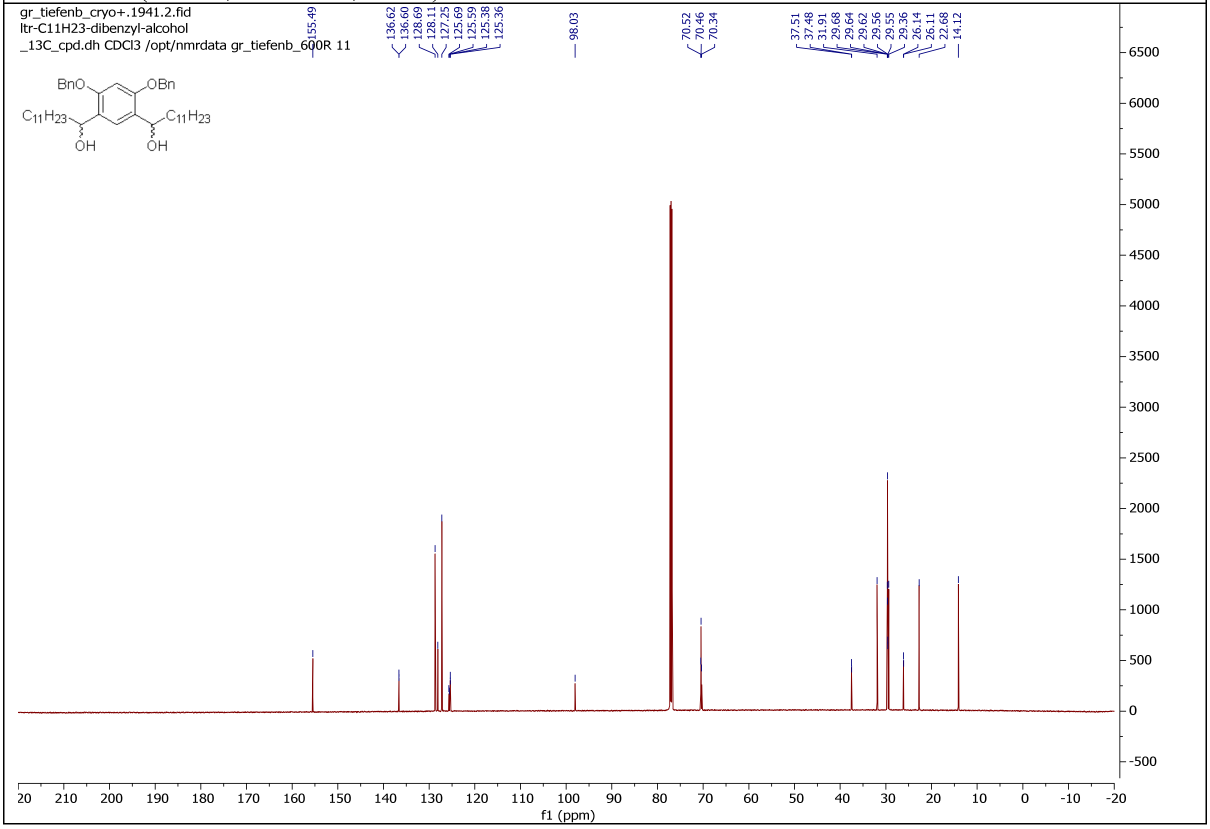

trans-1⁴,1⁶,3⁴,3⁶,5⁴,5⁶-hexakis(benzyloxy)-2,4-diisobutyl-1,3,5(1,3)-tribenzenacyclodecaphan-8-one **(11a)**

^{1}H NMR ($CDCl_3$, 600 MHz, 25 °C)

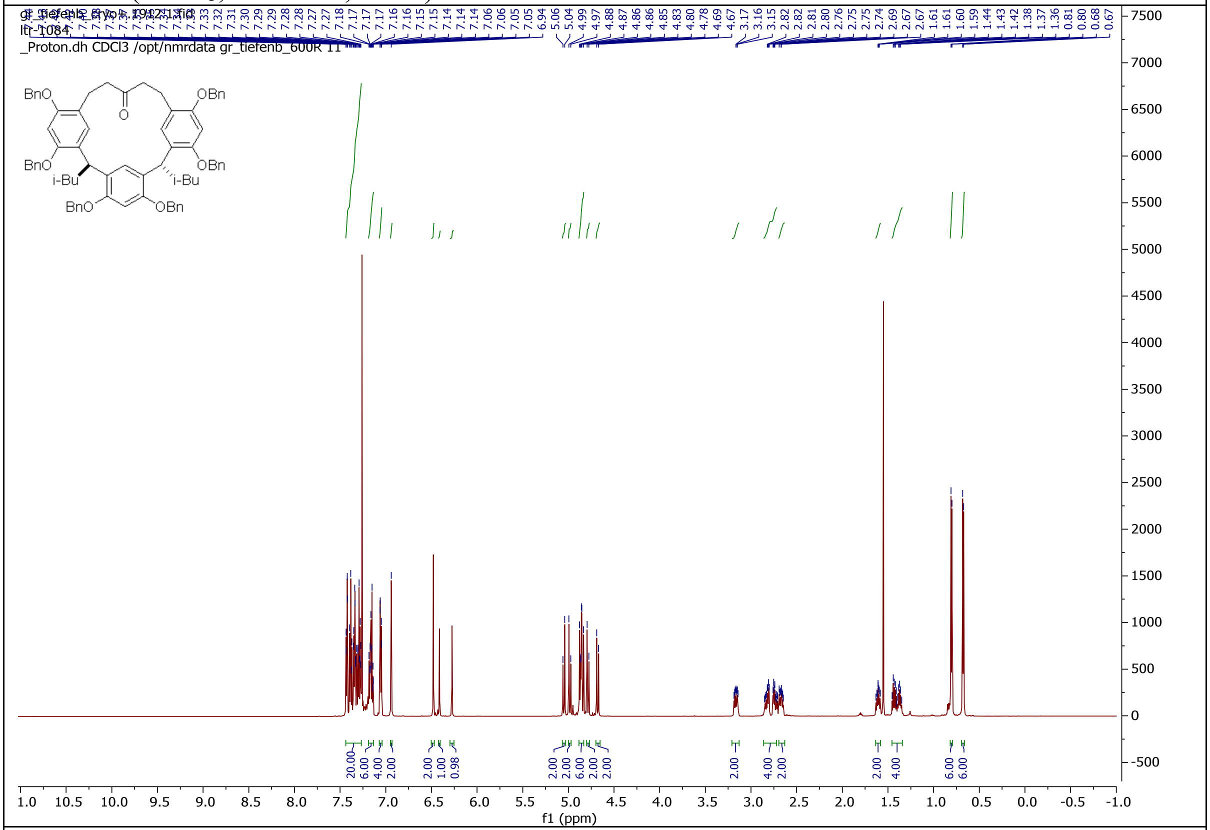

^{13}C NMR ($CDCl_3$, 150 MHz, 25 °C)

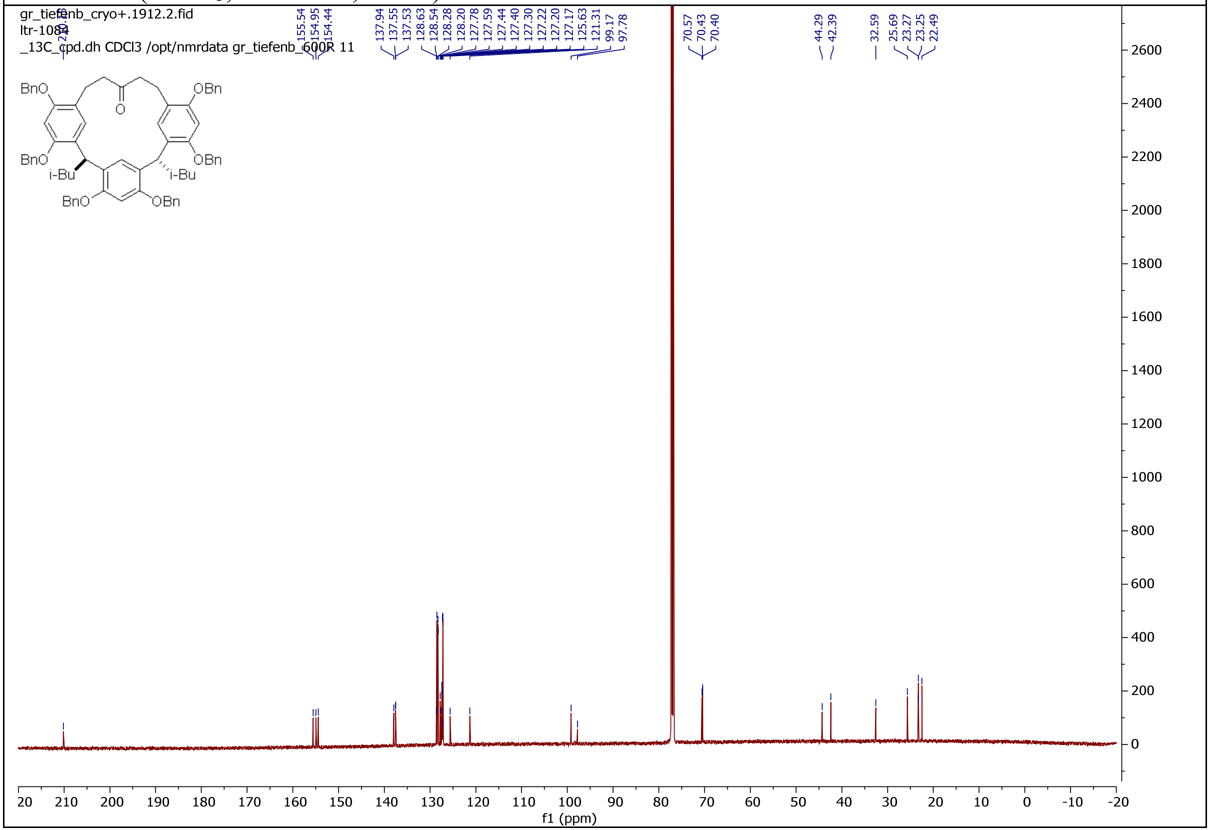

trans-$1^4,1^6,3^4,3^6,5^4,5^6$-hexakis(benzyloxy)-2,4-diundecyl-1,3,5(1,3)-tribenzenacyclodecaphan-8-one **(11b)**

^{1}H NMR (CDCl$_3$, 500 MHz, 25 °C)

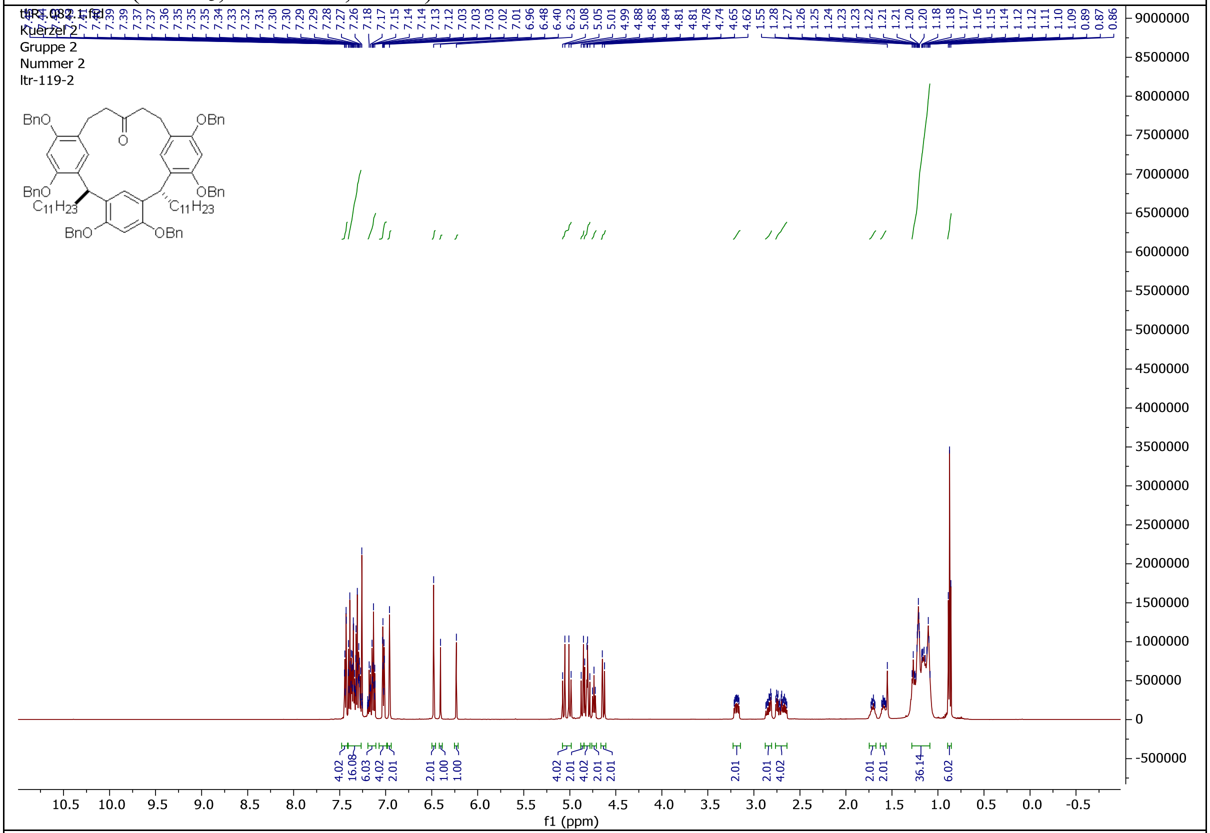

^{13}C NMR (CDCl$_3$, 125 MHz, 25 °C)

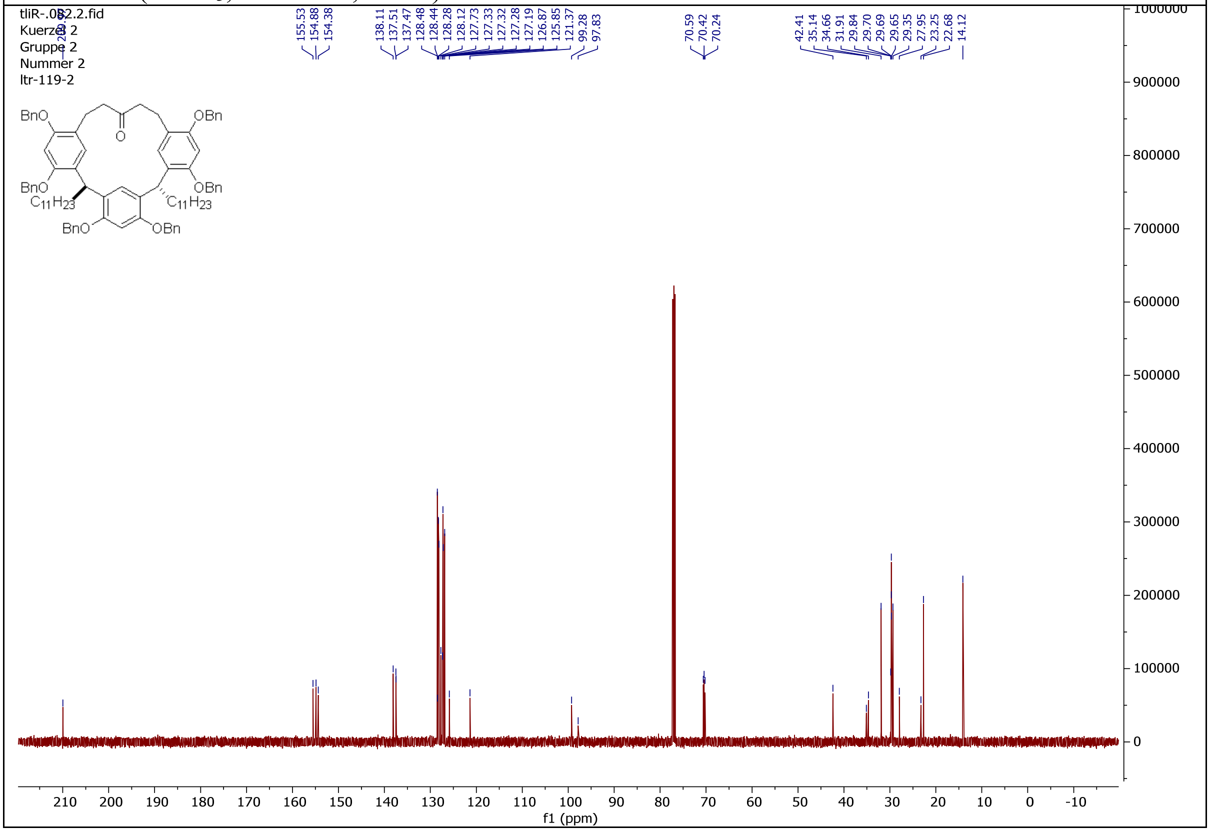

trans-1[4],1[6],3[4],3[6],5[4],5[6]-hexahydroxy-2,4-diisobutyl-1,3,5(1,3)-tribenzenacyclodecaphan-8-one (***trans*-2a**)

^{1}H NMR (acetone-D6, 600 MHz, 25 ºC)

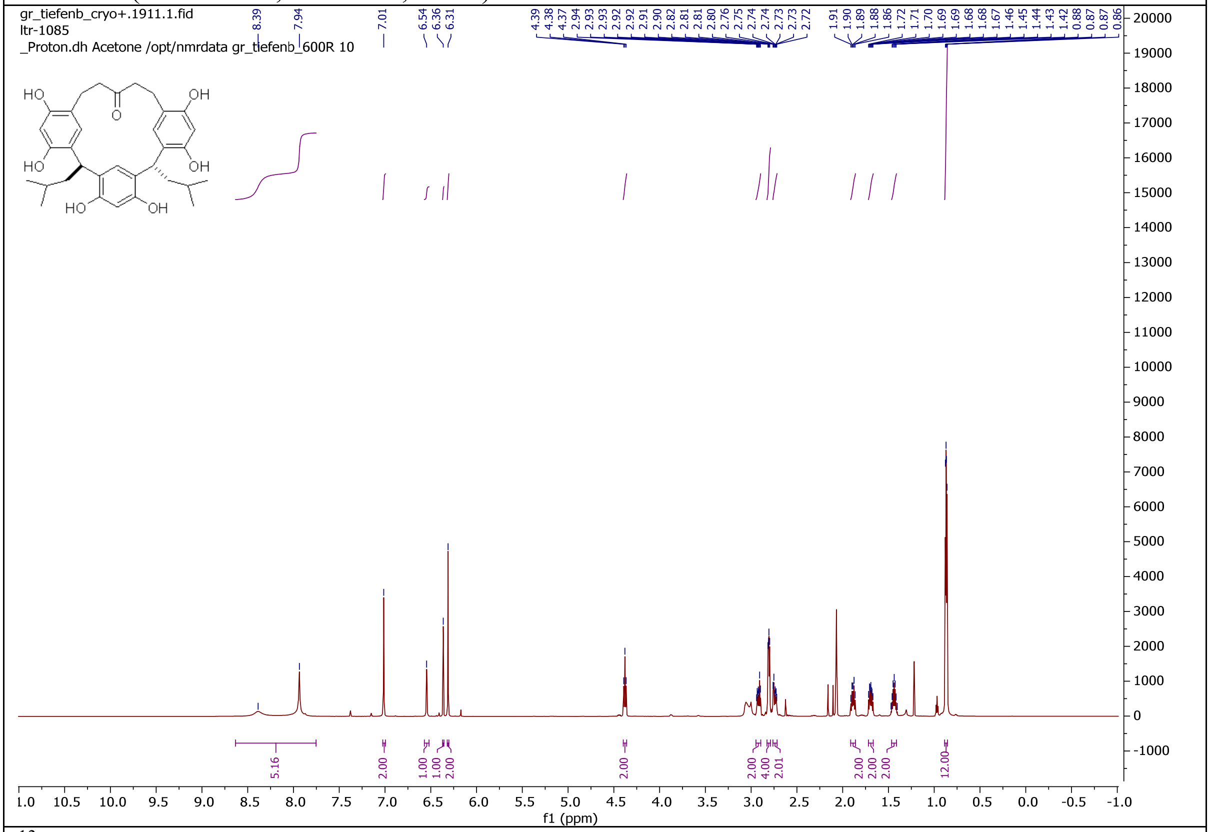

^{13}C NMR (acetone-D6, 150 MHz, 25 ºC)

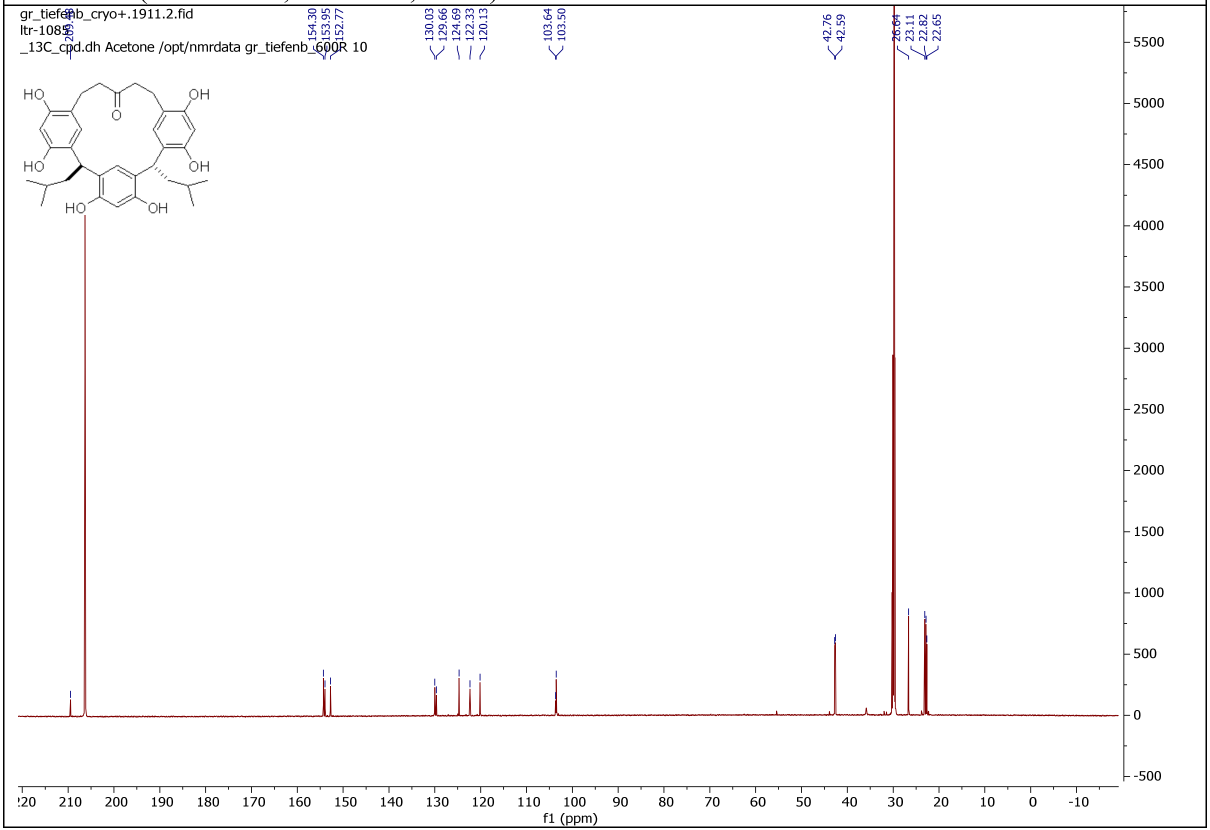

trans-$1^4,1^6,3^4,3^6,5^4,5^6$-hexahydroxy-2,4-diundecyl-1,3,5(1,3)-tribenzenacyclodecaphan-8-one (***trans*-2b**)

^{1}H NMR (acetone-D6, 500 MHz, 25 °C)

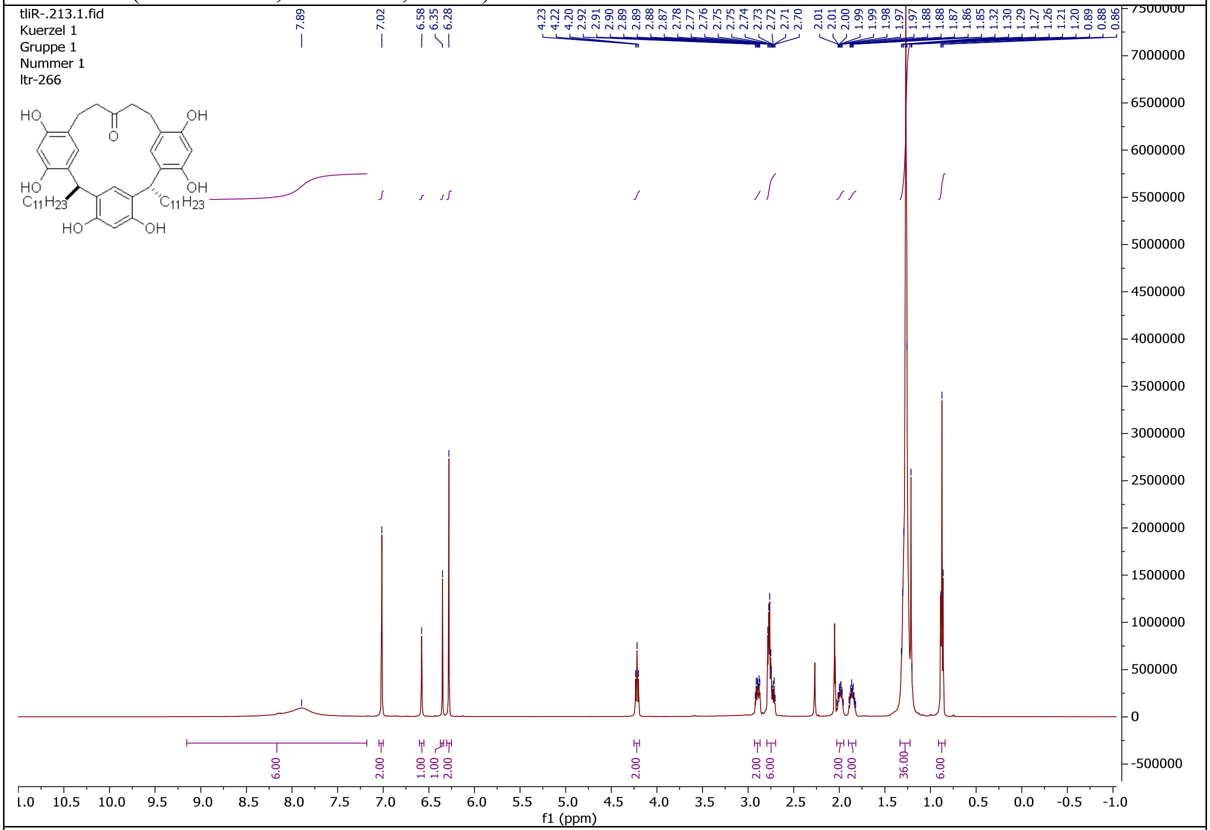

^{13}C NMR (acetone-D6, 125 MHz, 25 °C)

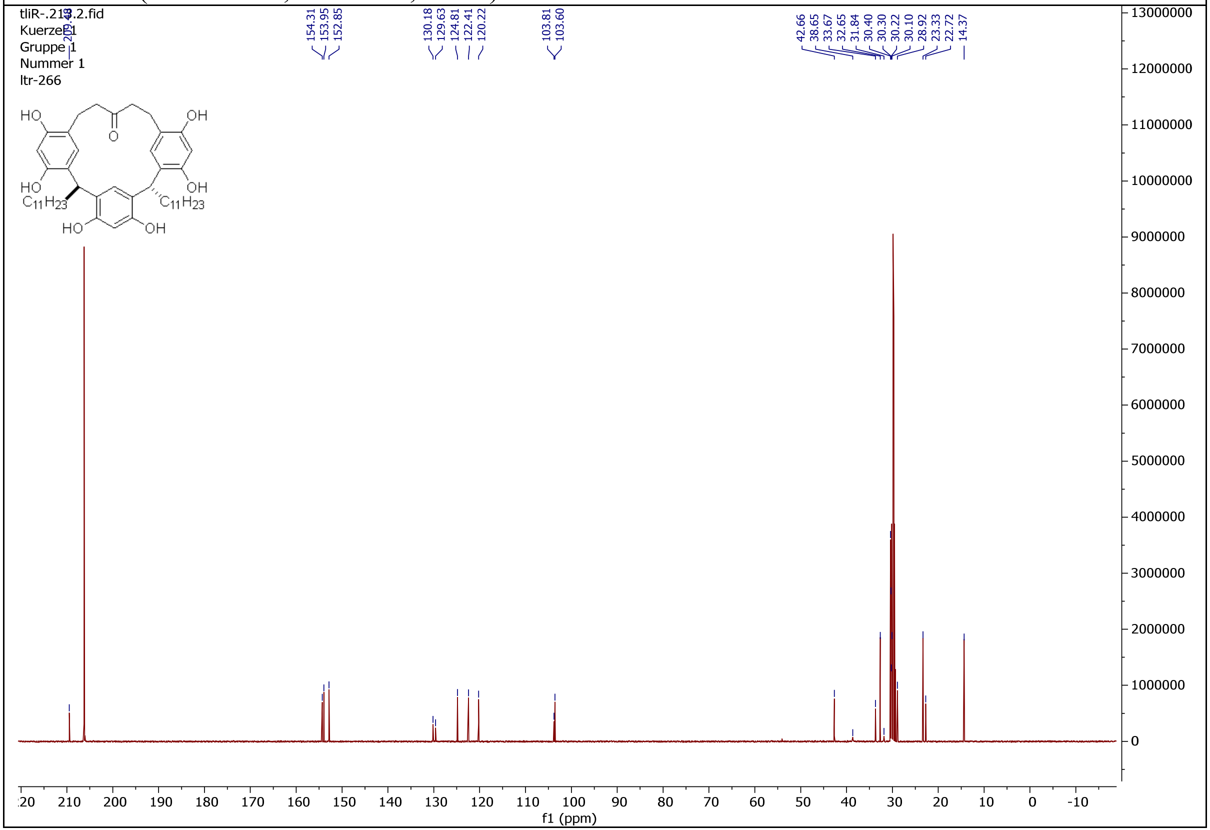

cis-$1^4,1^6,3^4,3^6,5^4,5^6$-hexahydroxy-2,4-diisobutyl-1,3,5(1,3)-tribenzenacyclodecaphan-8-one (***cis*-2a**)

^{1}H NMR (acetone-D6, 500 MHz, 25 °C)

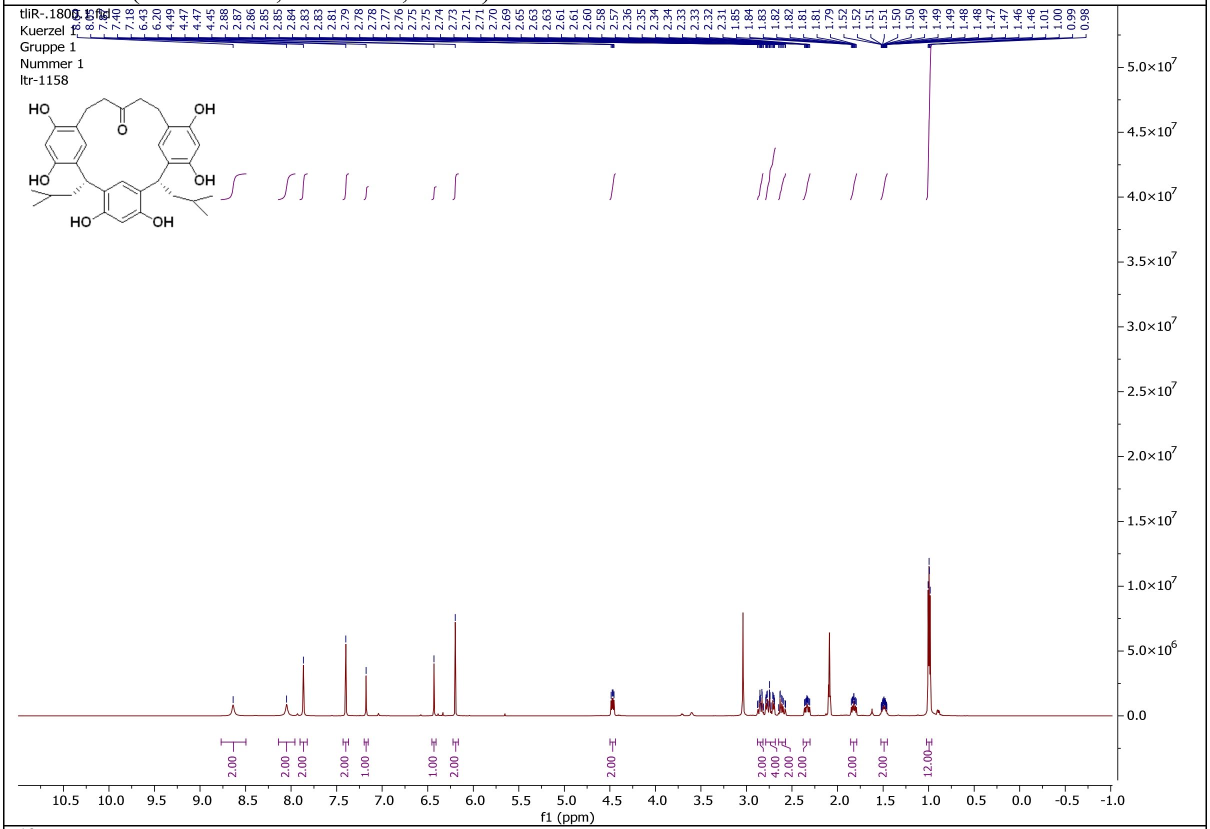

^{13}C NMR (acetone-D6, 125 MHz, 25 °C)

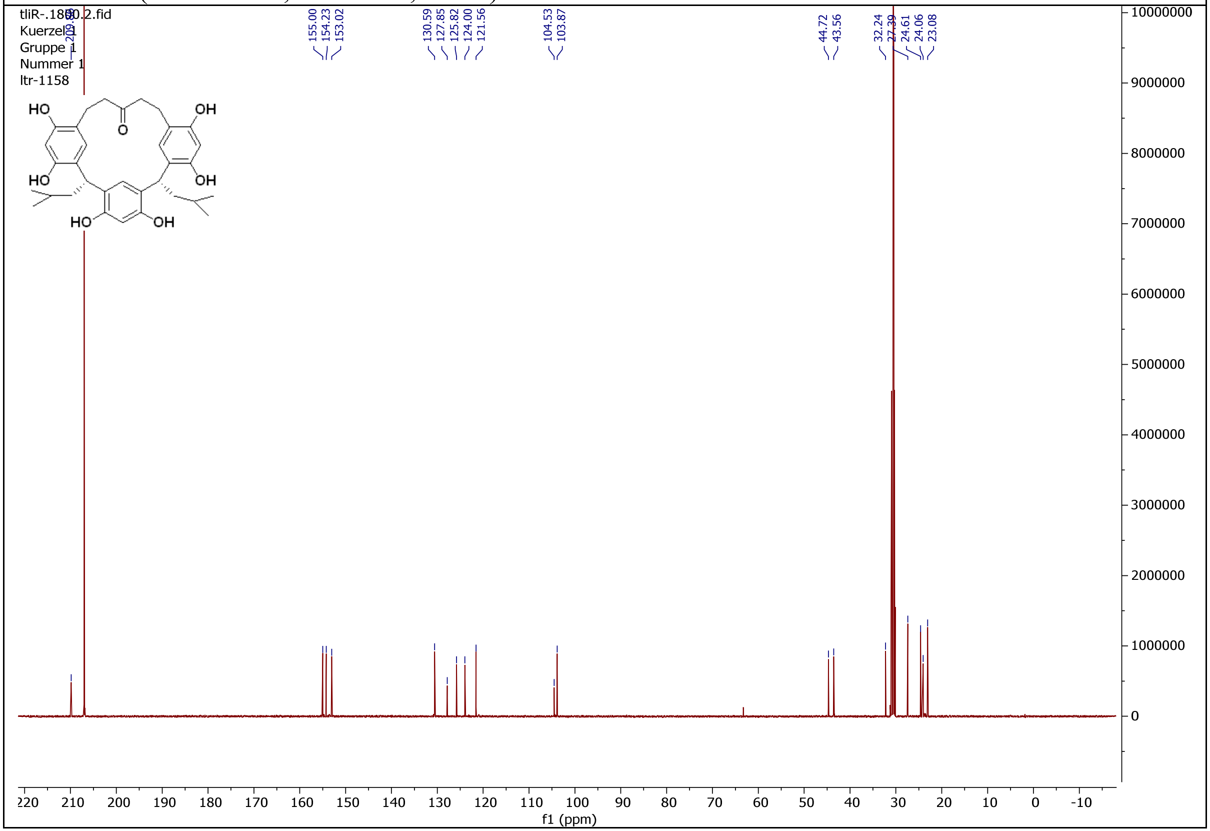

cis-$1^4,1^6,3^4,3^6,5^4,5^6$-hexahydroxy-2,4-diundecyl-1,3,5(1,3)-tribenzenacyclodecaphan-8-one **(*cis*-2b)**

^{1}H NMR (acetone-D6, 500 MHz, 25 °C)

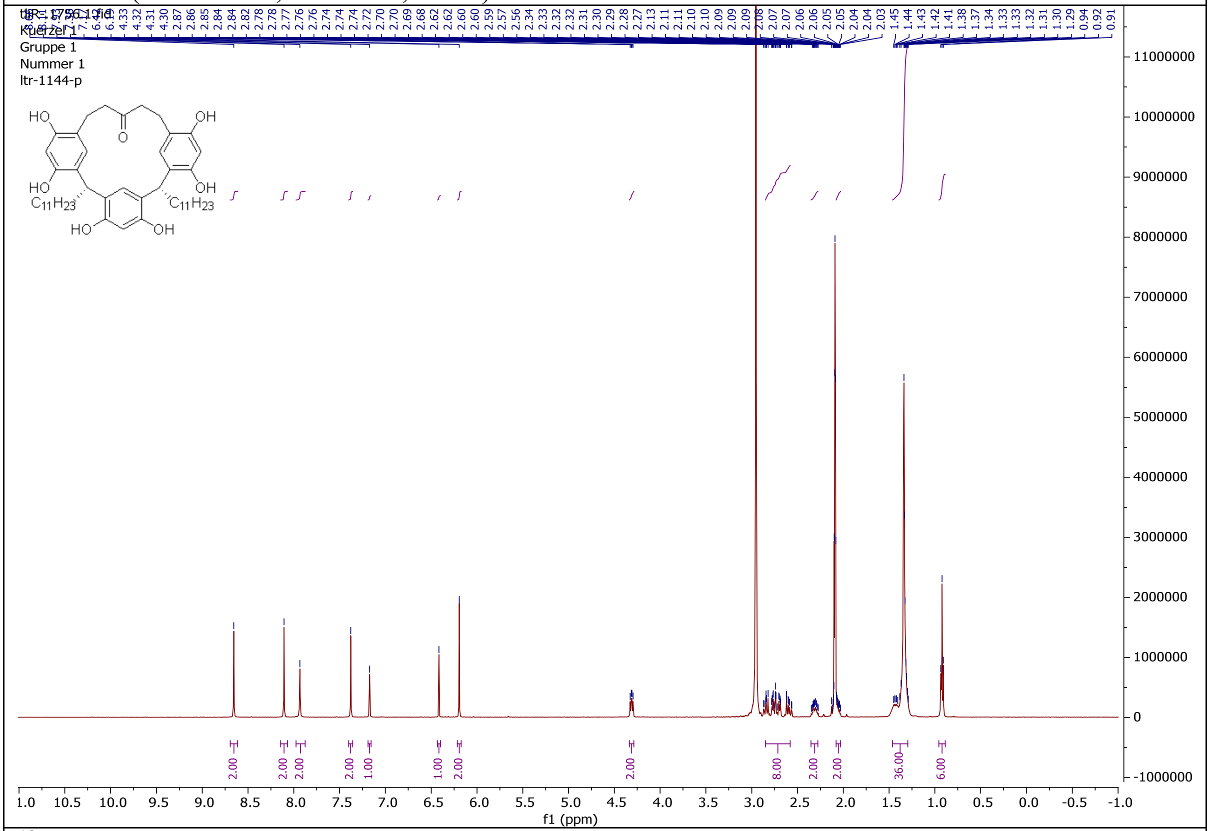

^{13}C NMR (acetone-D6, 125 MHz, 25 °C)

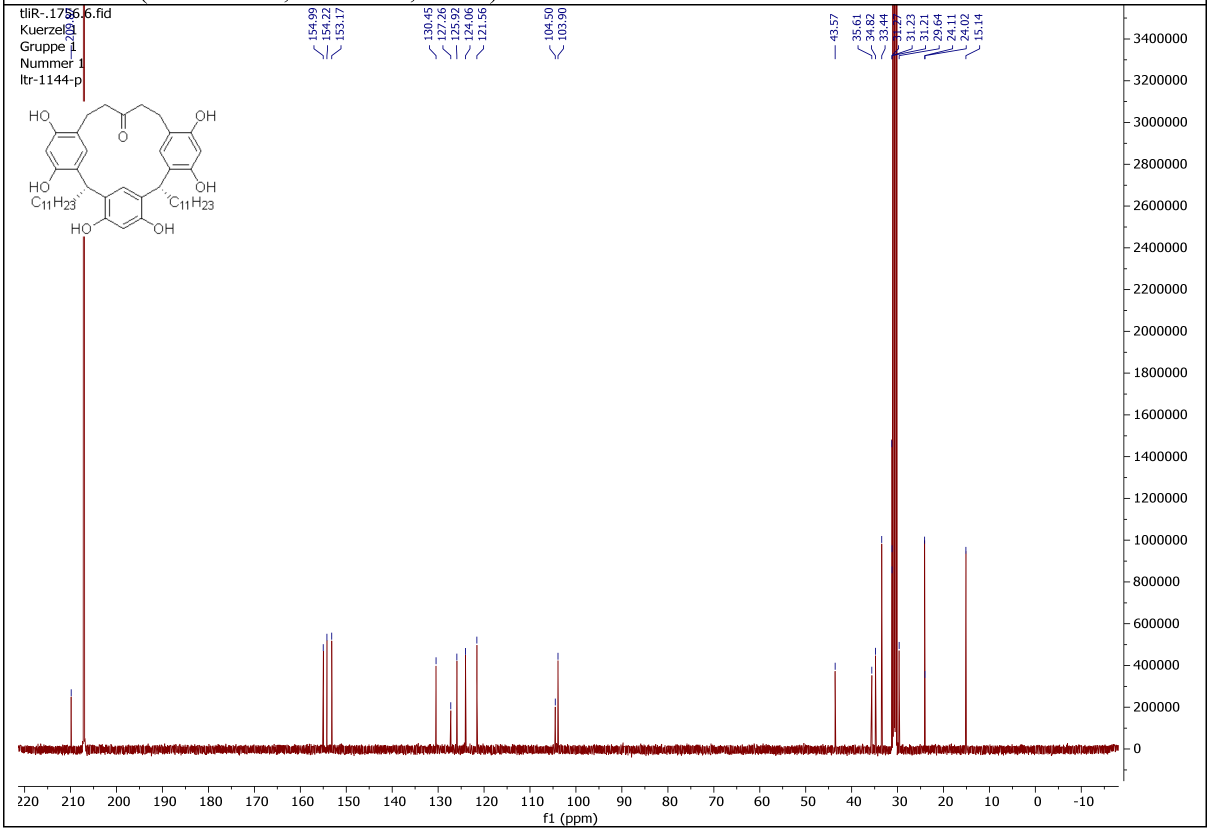

cis-2,4-diisobutyl-8-oxo-1,3,5(1,3)-tribenzenacyclodecaphane-$1^4,1^6,3^4,3^6,5^4,5^6$-hexayl hexaferrocenecarboxylate **(S1)**

^1H NMR ($CDCl_3$, 500 MHz, 25 °C)

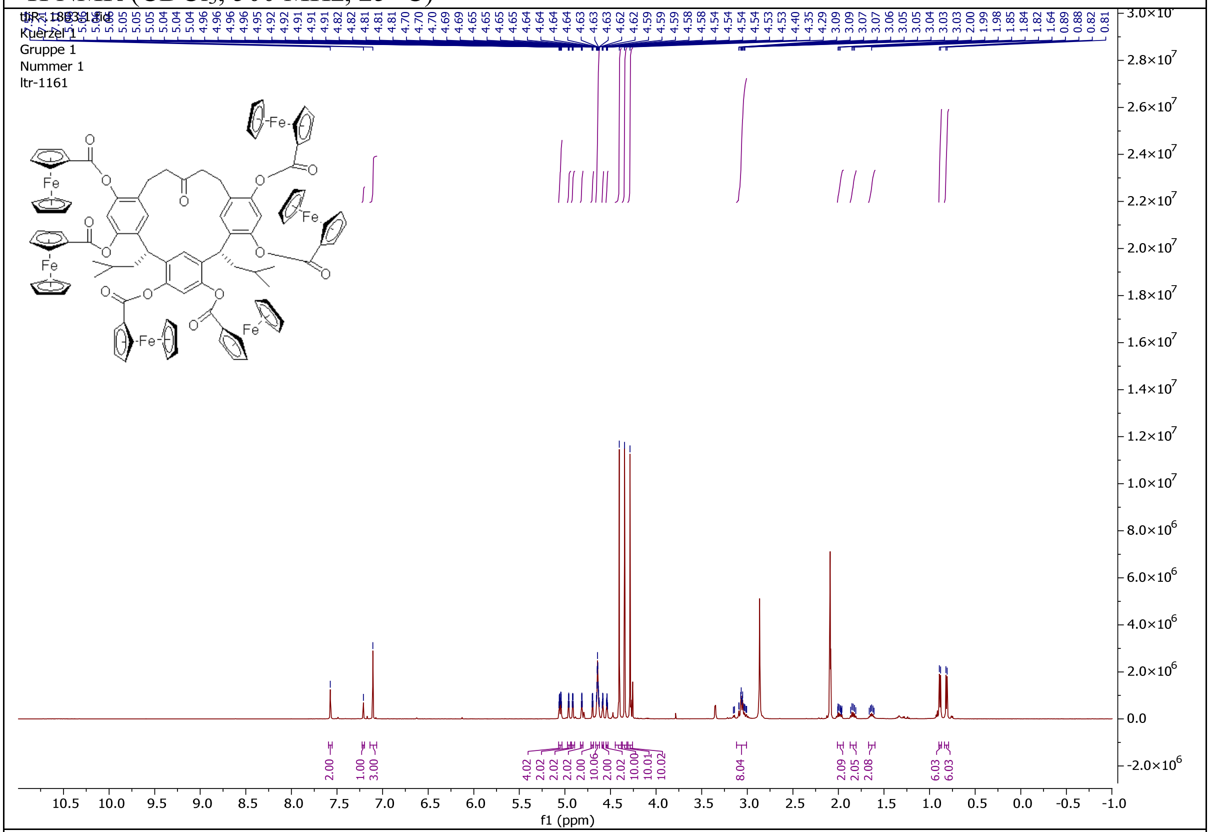

^{13}C NMR ($CDCl_3$, 125 MHz, 25 °C)

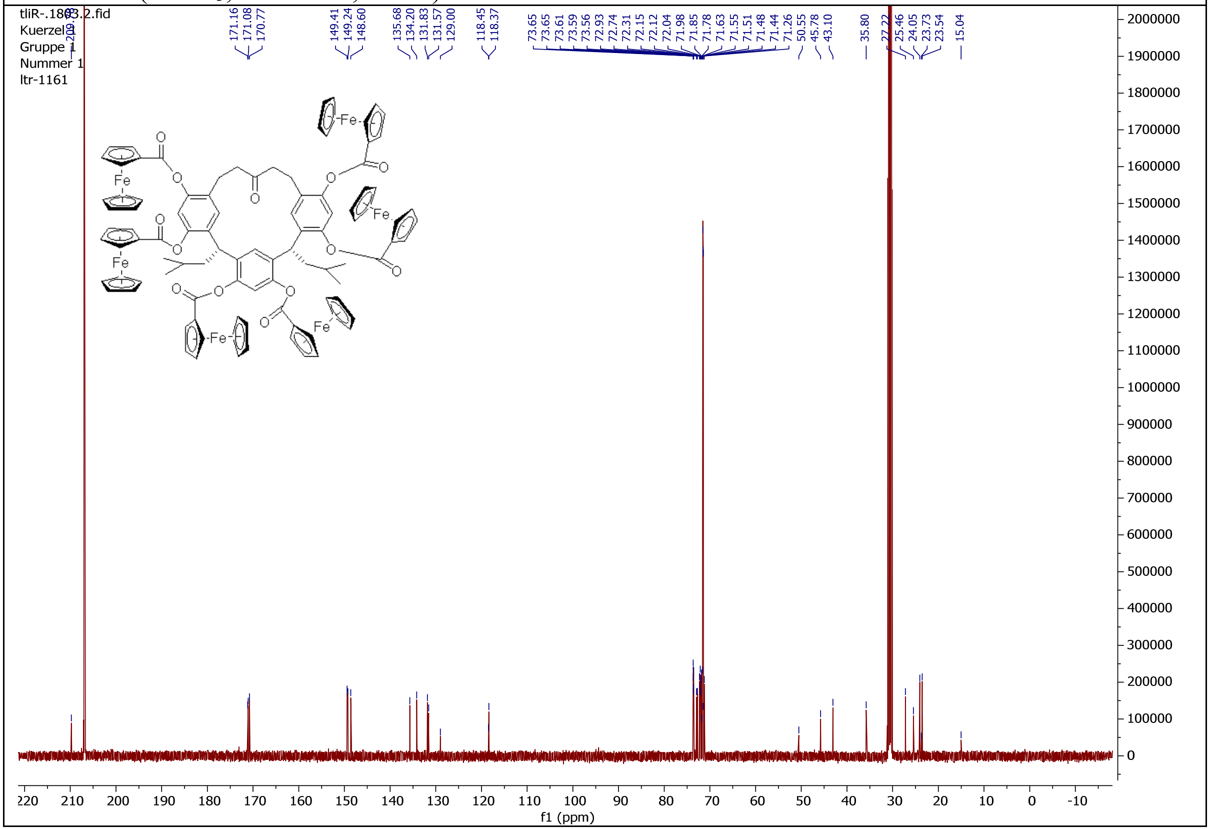

(4-octadecylphenyl)methanol **(S4)**

^{1}H NMR ($CDCl_3$, 600 MHz, 25 ºC)

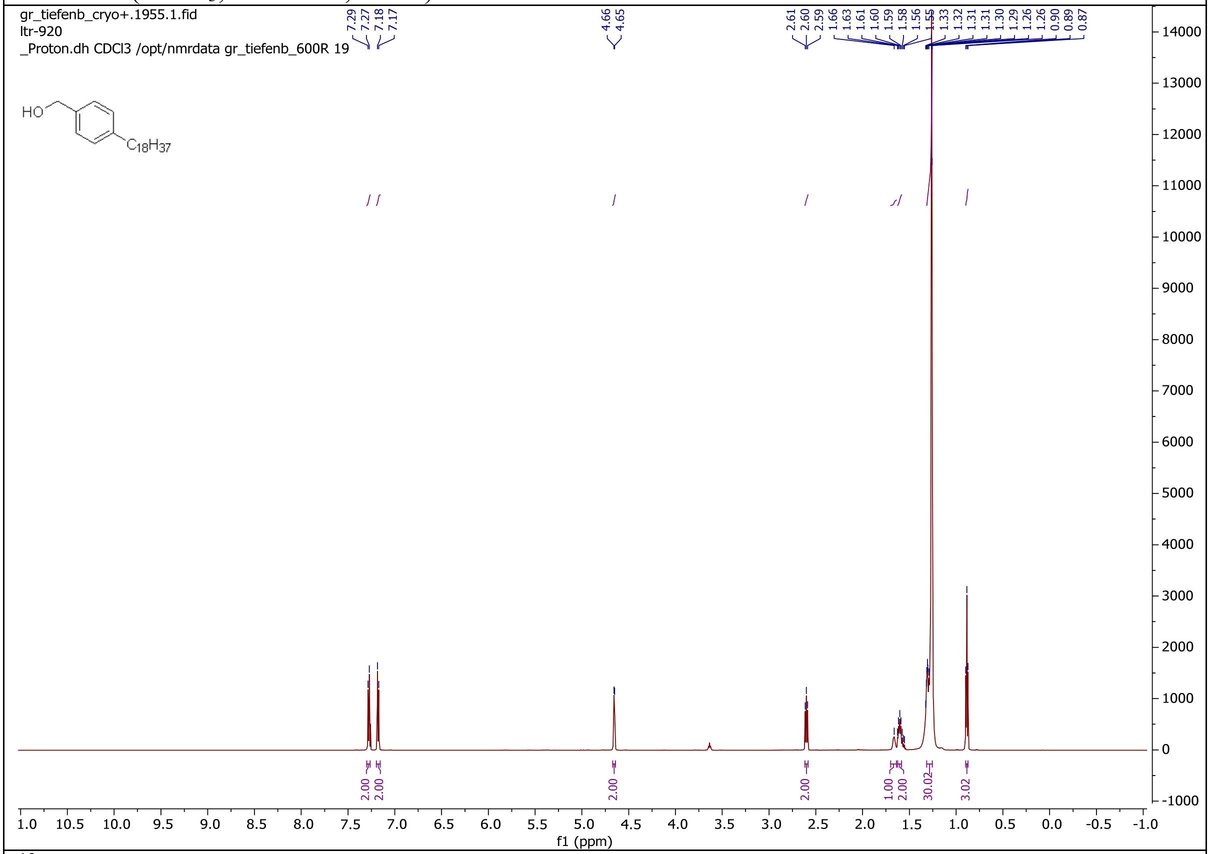

^{13}C NMR ($CDCl_3$, 150 MHz, 25 ºC)

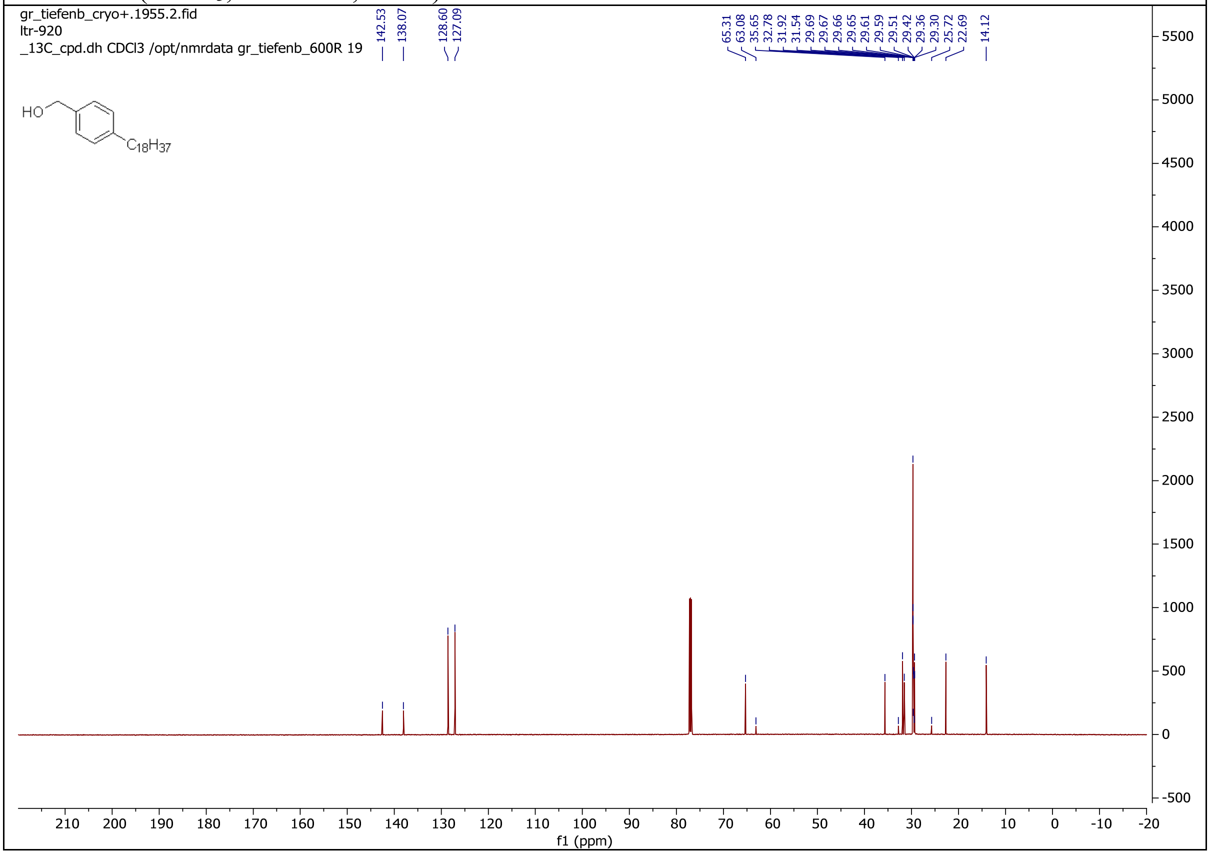

1-(chloromethyl)-4-octadecylbenzene **(13)**

^{1}H NMR ($CDCl_3$, 600 MHz, 25 °C)

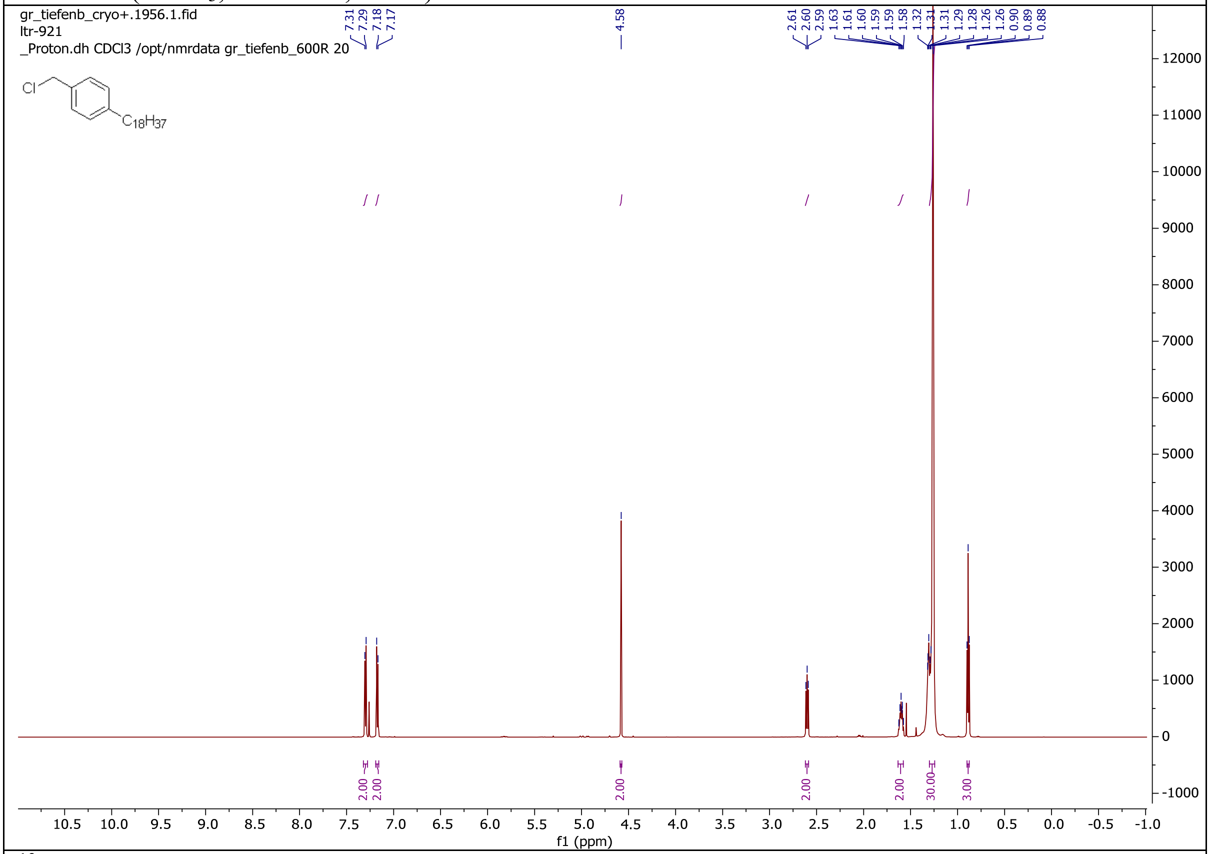

^{13}C NMR ($CDCl_3$, 150 MHz, 25 °C)

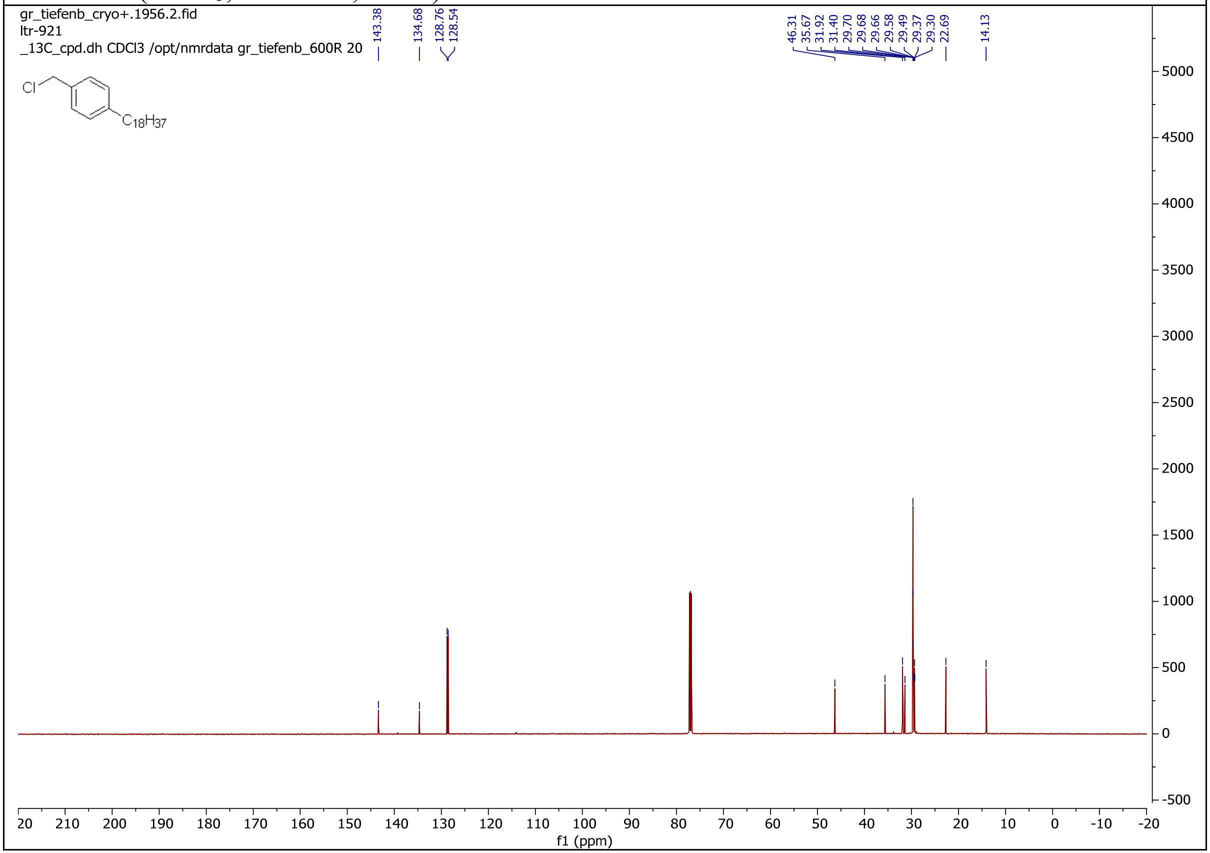

1,3,5-trimethoxy-2-(4-octadecylbenzyl)benzene **(15)**

^{1}H NMR ($CDCl_3$, 600 MHz, 25 °C)

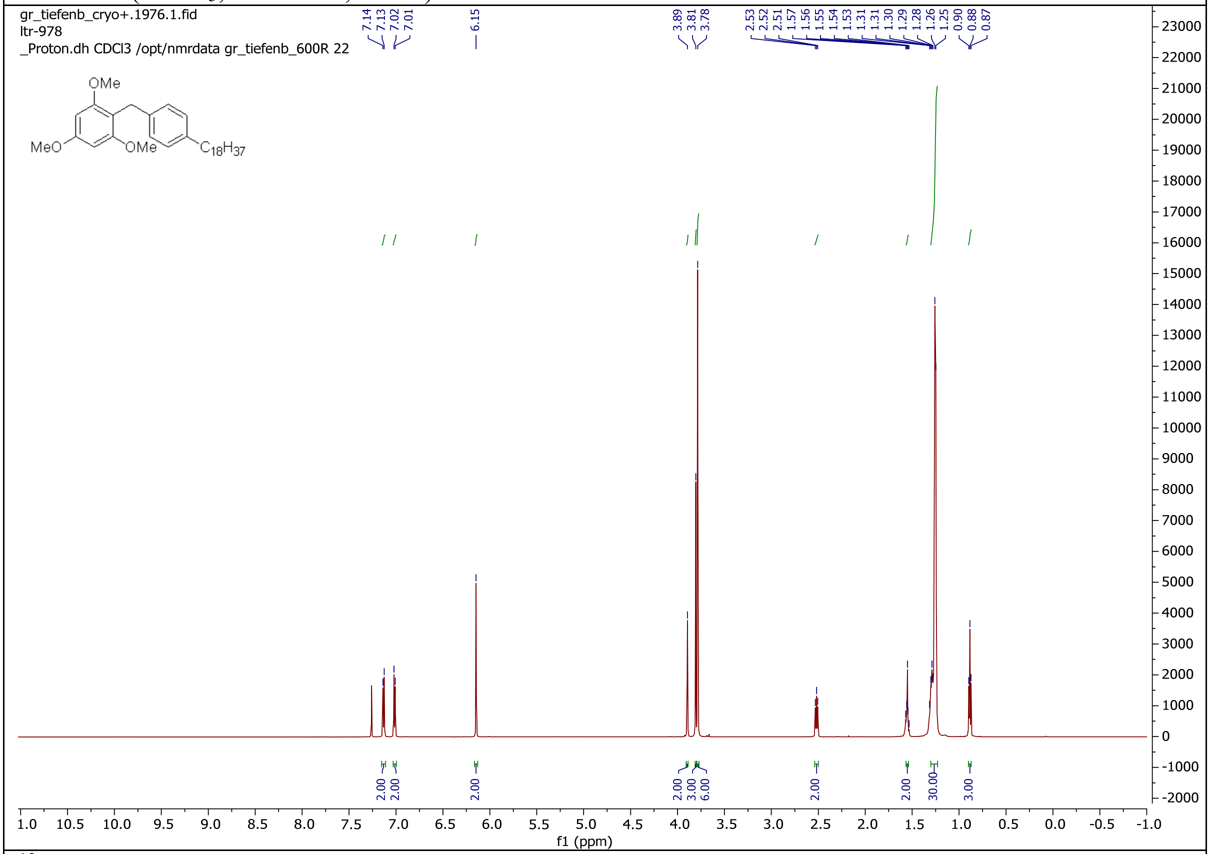

^{13}C NMR ($CDCl_3$, 150 MHz, 25 °C)

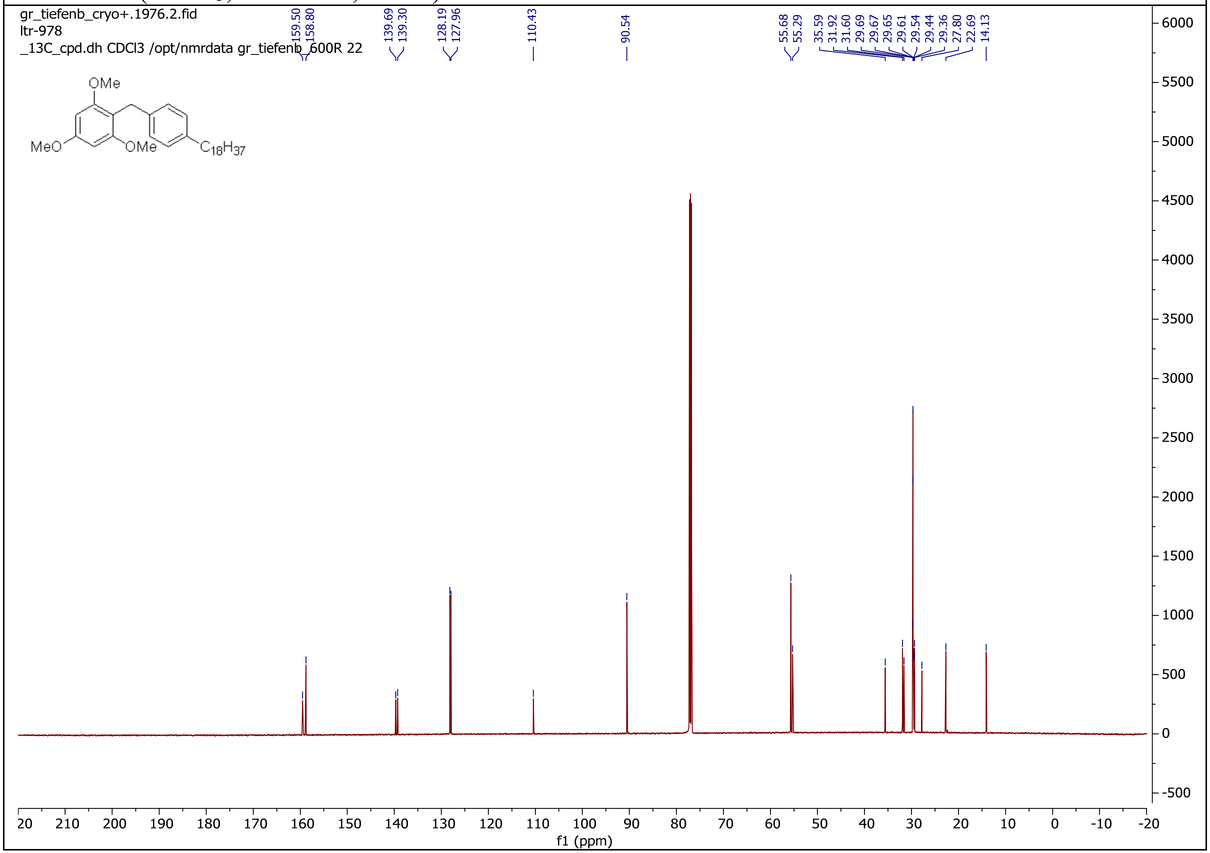

Supplement: Supplementary file 1 — au4c00097_si_001.pdf [file au4c00097_si_001.pdf]
